# Impfungen bei Immunsuppression, Immundefekten und immunmodulierenden Therapien: Empfehlungen – Update 2026

**DOI:** 10.1007/s00508-026-02754-4

**Published:** 2026-07-03

**Authors:** Ursula Wiedermann, Angelika Wagner, Alexander Bartuschka, Anna Sophie Berghoff, Alexandra Donschacher, Alexander Eser, Elisabeth Förster-Waldl, Lisa Göschl, Katharina Grabmeier-Pfistershammer, Felix Keil, Elisabeth König, Sabine Koppelstätter, Barbara Kornek, Robert Krause, Winfried F. Pickl, Clemens Scheinecker, Barbara Waas, Günter Weiss, Lukas Wisgrill, ISP-Arbeitsgruppe, Ursula Wiedermann, Angelika Wagner, Lisa Göschl, Clemens Scheinecker, Katharina Grabmeier-Pfistershammer, Alexander Eser, Barbara Kornek, Anna Sophie Berghoff, Alexandra Donschacher, Felix Keil, Elisabeth Förster-Waldl, Lukas Wisgrill, Winfried F. Pickl, Alexander Bartuschka, Barbara Waas, Alexander Bartuschka, Andrea Wessely, Sabine Koppelstätter, Angelika Wagner, Ursula Wiedermann, Christina Duftner, Robert Krause, Elisabeth König, Günter Weiss, Heinz Burgmann, Barbara Tucek, Daniela Philadelphy, Marcus Köller, Heidemarie Holzmann

**Affiliations:** 1 Zentrum für Pathophysiologie, Infektiologie, Immunologie, Institut für Spezifische Prophylaxe und Tropenmedizin, Medizinische Universität Wien, Kinderspitalgasse 15, 1090 Wien, Österreich; 2 Comprehensive Center for Inflammation and Immunity (CCII) und Comprehensive Center for Infection Medicine (CCIM), Medizinische Universität Wien, Universitätsklinikum AKH Wien, Wien, Österreich; 3 Zentrum für Pathophysiologie, Infektiologie, Immunologie, Institut für Spezifische Prophylaxe und Tropenmedizin, Medizinische Universität Wien, Wien, Österreich; 4 Rheumazentrum Oberlaa, Wien, Österreich; 5 Universitätsklinik für Innere Medizin I, Klinische Abteilung für Onkologie, Medizinische Universität Wien, Wien, Österreich; 6 3. Medizinische Abteilung – Hämatologie/Onkologie, Hanusch-Krankenhaus Wien, Wien, Österreich; 7 Abteilung für Innere Medizin I, Krankenhaus der Barmherzigen Brüder Wien, Wien, Österreich; 8 Universitätsklinik für Kinder- und Jugendheilkunde, Klinische Abteilung für Pädiatrische & Neonatale Immunologie, Pädiatrische Intensivmedizin und Neuropädiatrie, Medizinische Universität Wien, Wien, Österreich; 9 Comprehensive Center for Pediatrics (CCP) und Comprehensive Center for Inflammation and Immunity (CCII), Medizinische Universität Wien, Universitätsklinikum AKH Wien, Wien, Österreich; 10 Universitätsklinik für Innere Medizin III, Abteilung für Rheumatologie, Medizinische Universität Wien, Wien, Österreich; 11 Comprehensive Center for Inflammation and Immunity (CCII), Medizinische Universität Wien, Universitätsklinikum AKH Wien, Wien, Österreich; 12 Universitätsklinik für Dermatologie, Medizinische Universität Wien, Wien, Österreich; 13 Universitätsklinik für Innere Medizin, Klinische Abteilung für Infektiologie, Medizinische Universität Graz, Graz, Österreich; 14 Universitätsklinik für Innere Medizin II, Klinische Abteilung für Infektiologie, Immunologie, Tropenmedizin, Rheumatologie und Pneumologie, Medizinische Universität Innsbruck, Innsbruck, Österreich; 15 Universitätsklinik für Neurologie, Medizinische Universität Wien, Wien, Österreich; 16 Comprehensive Center for Clinical Neurosciences and Mental Health, Medizinische Universität Wien, Universitätsklinikum AKH Wien, Wien, Österreich; 17 Zentrum für Pathophysiologie, Infektiologie, Immunologie, Institut für Immunologie, Abteilung für Zelluläre Immunologie und Immunhämatologie, Medizinische Universität Wien, Wien, Österreich

**Keywords:** Angeborene und erworbene Immundefizienz, Immunsuppressionsgrad, Impferfolg, Impfversagen, Impfindikation bei Risikopopulationen, Biologika, Gezielte Therapien, CAR-T-Zell-Therapie, Inborn and acquired immunodeficiency, Degree of immunosuppression, Responsiveness/non-responsiveness to vaccination, Vaccine indication in risk populations, Biologics, Targeted therapies, CAR-T cell therapy

## Abstract

Eine Immunsuppression unterschiedlicher Genese geht mit einem erhöhten Risiko diverser Infektionen einher. Die Impfprävention besitzt daher bei immunsupprimierten Patientinnen und Patienten einen hohen Stellenwert.

Auf Grundlage der verfügbaren Evidenz sowie immunologischer und theoretischer Überlegungen wurden 2016 erstmals Empfehlungen zur Impfversorgung immunsupprimierter Personen formuliert. Diese Empfehlungen wurden nun umfassend aktualisiert und erweitert. Ziel ist die Bereitstellung einer praxisorientierten, indikations- und therapiebezogenen Handlungsanleitung für die klinische Versorgung. Eine Anleitung für die vorliegenden Impfempfehlungen, die gleichzeitig als Entscheidungsbaum für das praktische Vorgehen dienen kann, erleichtert die Orientierung.

Die vorliegenden Empfehlungen umfassen einen einführenden Abschnitt zu grundlegenden Aspekten der Immunsuppression sowie zu praxisrelevanten immunologischen und organisatorischen Fragestellungen im Zusammenhang mit Impfplanung, Ermittlung der Impffähigkeit und Überprüfung des Impferfolgs. Darüber hinaus werden aktuelle Entwicklungen zu relevanten Impfstoffen berücksichtigt.

Im weiteren Verlauf erfolgt eine systematische Darstellung der in verschiedenen medizinischen Fachgebieten häufig eingesetzten immunsuppressiven Wirkstoffe. Diese werden hinsichtlich ihres Immunsuppressionsgrades und der damit verbundenen Infektionsrisiken eingeordnet. Ergänzend werden standardisierte Impfschemata bereitgestellt, die den jeweiligen Wirkstoffklassen zugeordnet sind und eine strukturierte Orientierung im klinischen Alltag ermöglichen. Die Evaluierung der Impffähigkeit kann immer nur im Zusammenspiel zwischen Grunderkrankung und geplanter oder bestehender Therapie getroffen werden.

Besonderen klinischen Situationen – einschließlich jenen, in denen Impfungen nicht durchgeführt werden können sowie Reiseimpfungen im Kontext der Immunsuppression – sind eigene Kapitel gewidmet. Zur Sicherstellung der längerfristigen Aktualität wird an geeigneten Stellen auf aktualisierte externe Quellen verwiesen, die gesammelt am Ende des Dokuments aufgelistet sind.

Dieses Dokument ist eine allgemeine Empfehlung, ohne Anspruch auf Vollständigkeit. Das spezifische Vorgehen in der klinischen Praxis kann an die Gegebenheiten in den einzelnen Fachgebieten angepasst werden und es wird auf die jeweiligen Fachgesellschaften verwiesen.

## Abstract (en)

Immunosuppression of various origins is associated with an increased risk of infections and infection-triggered morbidity and mortality. Vaccination, therefore, plays a central role in the care of immunosuppressed individuals by protecting against vaccine-preventable diseases.

Based on the available evidence as well as immunological and theoretical considerations, an expert statement on vaccination in immunocompromised individuals was first formulated in 2016. These recommendations have herewith been comprehensively updated and expanded. The aim is to provide a practice‐oriented, indication‐ and therapy-specific guidance document for clinical care. A practical chart accompanying the present vaccination recommendation, which also functions as a decision tree for implementation, facilitates orientation within the document.

The document includes an introductory section outlining fundamental aspects of immunosuppression and addressing immunological and organizational issues relevant to vaccination planning, application, and control of vaccination responsiveness. In addition, current developments regarding relevant vaccines are reviewed.

Subsequently, a systematic overview of immunosuppressive agents commonly used across medical specialties is provided. These agents are categorized according to their degree of immunosuppression and the associated infection risks. Standardized vaccination schedules are provided and assigned to the respective drug classes to facilitate a structured approach in clinical practice. The decision on vaccination eligibility of these patients can only be made based on a comprehensive assessment of the underlying disease and the planned or ongoing immunosuppressive therapy.

Special clinical situations—including circumstances in which vaccination cannot be performed, as well as travel‐related vaccinations in the context of immunosuppression—are addressed in separate chapters. To ensure long‐term relevance, the document refers to continuously updated external sources throughout, which are compiled at the end.

This document acts as general recommendation without a claim of completeness. The specific procedure in clinical practice can be adapted to the clinical context, and the reader is referred to the respective professional societies.

## Inhaltsverzeichnis

1: Einleitung
2: Theoretischer Hintergrund und Updates
2.1: Immunsuppression und Immunmodulation
2.2: Schweregrade der Immunsuppression
2.3: Impffähigkeit in Abhängigkeit des Schweregrads der Immunsuppression
2.4: Zeitliche Abstände zwischen Impfung und Immunsuppression
2.4.1: Vor Therapiebeginn
2.4.2: Während einer immunsuppressiven Therapie
2.4.3: Nach einer immunsuppressiven Therapie
2.4.4: Lebendimpfungen im Therapieintervall bei Grad-III-Immunsuppression
3: Immunstatus und Impferfolg
3.1: Erhebung des aktuellen Impfstatus/Immunschutzes bei Lebendimpfungen
3.2: Bestimmung der Impffähigkeit/Immunkompetenz von Personen mit Immunsuppression mittels diagnostischer Impfung
3.3: Immunoprofiling unter Therapie
3.4: Titerbestimmung zur Impferfolgskontrolle
3.5: Immunsystem, Impfung und therapieinduzierte Immunsuppression
4: Grundlagen zu Impfstoffen – Update
4.1: Arten von Impfstoffen
4.1.1: Lebendimpfstoffe
4.1.2: Inaktivierte/nicht-lebend-Impfstoffe
4.2: Adjuvanzien-Update
4.3: Impfstoff-Update
4.3.1: COVID-19
4.3.2: Respiratorisches Synzytialvirus (RSV)
4.3.3: Influenza
4.3.4: Herpes Zoster
4.3.5: Meningokokken
4.3.6: Pneumokokken
5: Therapie-Update spezifischer Fachgebiete, Infektionsrisiko und Impfindikation
5.1: Rheumatologie
5.2: Dermatologie
5.3: HIV
5.4: Gastroenterologie
5.5: Neurologie
5.6: Onkologie und Hämatoonkologie
5.6.1: Solide Tumoren
5.6.2: Hämatologische Neoplasien
5.7: Solide Organtransplantation
5.8: Asplenie, Splenektomie
5.9: Angeborene („primäre“) Immundefekte
5.10: Immunsupprimierte Kinder (sekundäre Immundefizienz)
5.11: Biologika in Schwangerschaft und Stillzeit – Impfen von Kindern behandelter Mütter
6: Praktisches Vorgehen bei Immunsuppression: konkrete Impfempfehlungen nach Substanzklassen/Therapiearten
6.1: Impfschema für lebend-attenuierte Impfstoffe
6.2: Impfschemata für inaktivierte Impfstoffe – nach Indikation
6.2.1: Impfschema A – Routineimpfungen
6.2.2: Impfschema B – saisonale Impfungen
6.2.3: Impfschema C – ergänzende Impfungen vor oder unter Therapie mit Immunsuppressiva Grad III
6.2.4: Impfschema D – Impfungen gegen bekapselte Bakterien
6.3: Anwendung der Impfschemata nach Indikation
6.3.1: Angeborene/primäre Immundefekte
6.3.2: Konventionelle Therapeutika/Immunsuppressiva (Grad I–III)
6.3.3: JAK-Inhibitoren (Grad IIIa)
6.3.4: Komplementinhibitoren (Grad II–IIIa)
6.3.5: T‑Zell-Inhibitoren (Grad IIIa)
6.3.6: B‑Zell-Inhibitoren/Depletoren (Grad IIIb)
6.3.7: Checkpoint-Inhibitoren
6.3.8: Zielgerichtete Therapien in der Onkologie/Hämatoonkologie
6.3.9: Ergänzende immunsuppressive Medikamente mit ISP-Grad III
6.3.10: Immunmodulatoren mit ISP-Grad 0–II
6.3.11: Nach Stammzelltransplantation (autolog, allogen)
6.3.12: Vor und nach CAR-T- und anderen Zell-basierten Immuntherapien
6.3.13: Impfungen bei solider Organtransplantation
6.3.14: Impfungen bei HIV
6.3.15: Impfen bei Asplenie/Hyposplenie
7: Wenn nicht geimpft werden kann
7.1: Immunglobuline
7.2: Antivirale Prophylaxe und Therapie
7.3: Umgebungsprophylaxe
8: Kurzer Überblick zu Reiseimpfungen bei Immunsuppression/Immundefekten
8.1: Gelbfieber
8.1.1: Anwendung der Gelbfieberimpfung in besonderen Fällen
8.2: Tollwut
8.3: Dengue
8.4: Chikungunya
8.5: Mpox
8.6: Ebola
9: Wichtige Quellen zu jeweils aktuellen Informationen

Anhang

10: Appendix 1 Abkürzungen
11: Appendix 2 Tabellen- und Abkürzungsverzeichnis

Literatur

## 1 Einleitung

Primäre und sekundäre Immundefekte, verschiedene erworbene Erkrankungen sowie Behandlungen und operative Eingriffe können mit einer temporären oder permanenten Immunsuppression (ISP) einhergehen. Personen mit einem supprimierten Immunsystem sind einem erhöhten Infektionsrisiko ausgesetzt. Aufgrund ihrer Immuninsuffizienz sind im Falle einer Infektion schwerere Verläufe zu erwarten [1, 2]. Das Erkennen einer möglichen Immuninsuffizienz in Verbindung mit entsprechenden präventiven Maßnahmen ist daher wichtig. Für impfpräventable Infektionen steht eine stetig wachsende Zahl an Impfungen zur Verfügung. Die Impfquote bei immunsupprimierten Personen ist allerdings oftmals noch gering [3, 4]. Dies liegt nicht zuletzt an fehlender Information zu Impfungen und den damit verbundenen Bedenken der Behandelten und ihrer Angehörigen wie auch des betreuenden Gesundheitspersonals gegenüber Impfungen in diesem Setting, aber auch daran, dass das Ausmaß der möglichen individuellen Immunkompetenz oft nicht bekannt ist [5].

Seit der „Coronavirus disease 2019“(COVID-19)-Pandemie hat sich allerdings das Bewusstsein für das verstärkte Infektionsrisiko und den potenziell hohen Erkrankungsschweregrad infolge einer chronischen Erkrankung und/oder Immunsuppression deutlich erhöht. In der Praxis zeigt sich jedoch, dass es immer schwieriger wird, mit der wachsenden Anzahl an neuen immunsuppressiven Therapien Schritt zu halten und dass die durch diese Therapien entstehende Immunsuppression und das sich daraus ergebende Infektionsrisiko oftmals unterschätzt werden. Es ist daher wichtig, im ärztlichen Gespräch gezielt nach Grundkrankheit und immunsuppressiver Therapie zu fragen.

Ziel des vorliegenden Dokuments ist es daher, praxisnahe und möglichst allgemeingültige Impfempfehlungen für betroffene Personengruppen auszusprechen. Insbesondere wird auf diverse Indikationen mit krankheits- oder medikationsbedingter Immunsuppression in den Bereichen Rheumatologie, Dermatologie, humanes Immundefizienzvirus (HIV), Gastroenterologie, Neurologie, Onkologie und Hämatoonkologie, hämatopoetische Stammzell- und solide Organtransplantation, Asplenie und Splenektomie, angeborene Immundefekte, Kindesalter und Schwangerschaft eingegangen. Dem Thema Reiseimpfungen bei Risikopersonen ist ein gesonderter, allerdings komplexitätsbedingt kurz gehaltener Abschnitt gewidmet.

**Abb. 1:**
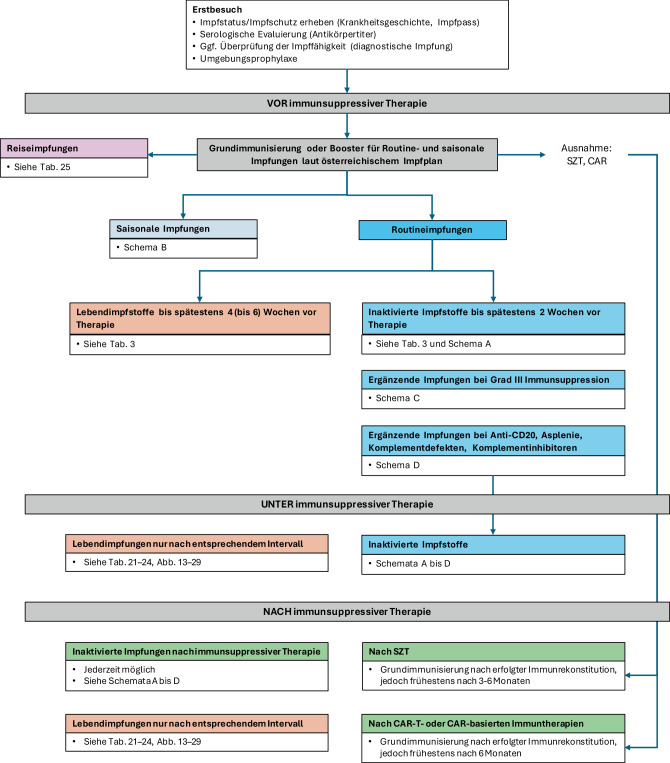
Allgemeines Vorgehen zu Impfungen bei Personen mit Immunsuppression. Farbschema: *rosa* Reiseimpfungen, *hellblau* saisonale Impfungen, *orange* Lebendimpfstoffe, *türkis* inaktivierte Impfstoffe, *grün* nach Immunsuppression (ausgenommen Lebendimpfstoffe). *CAR* chimärer Antigenrezeptor, *ggf.* gegebenenfalls, *SZT* Stammzelltransplantation

Abb. 1 stellt eine Anleitung für die vorliegende Impfempfehlung und gleichzeitig einen Entscheidungsbaum für Impfungen bei Personen mit Immunsuppression dar. Ein Tabellen- und ein Abbildungsverzeichnis finden sich im Anhang.

Im Bewusstsein der epidemiologischen Entwicklungen bei Infektionserkrankungen und der Entwicklung neuer Impfstoffe einerseits und neuer Biologika und zielgerichteter Therapien andererseits soll jedoch auch ein lebendes Dokument vorgelegt werden, das durch Verlinkung auf regelmäßig aktualisierte Quellen, wie z. B. den österreichischen Impfplan, das Österreichische Bundesamt für Sicherheit im Gesundheitswesen (BASG), die European Medicines Agency (EMA), das Robert-Koch-Institut (RKI) oder das Paul-Ehrlich-Institut (PEI), seine Gültigkeit auch längerfristig behält. Im Fließtext sowie in einem eigenen Abschnitt 9 am Ende des Dokuments wird auf relevante Quellen mittels QR-Code auf die jeweilige Website verlinkt.

Dieses Dokument ist eine allgemeine Empfehlung, ohne Anspruch auf Vollständigkeit. Die vorliegenden Impfempfehlungen beruhen auf der Synthese rezenter Literatur, bestehend aus internationalen Impfempfehlungen, Reviews, Metaanalysen und Originalarbeiten. Dort, wo keine spezifischen Empfehlungen und entsprechende Evidenz vorliegen, wurde versucht, Vorgehensweisen auf der Basis theoretischer Überlegungen zu formulieren. Das spezifische Vorgehen in der klinischen Praxis kann an die Gegebenheiten in den einzelnen Fachgebieten angepasst werden und es wird auch auf die jeweiligen Fachgesellschaften verwiesen.

## 2 Theoretischer Hintergrund und Updates

### 2.1 Immunsuppression und Immunmodulation

Neben primären Immundefekten führen bestimmte Erkrankungen (z. B. hämatoonkologische Erkrankungen) per se oder die jeweiligen Therapien zu unterschiedlichen Schweregraden der Immunsuppression. Unter eine medikamentöse immunsuppressive Therapie fallen Medikamente, die eine breite Unterdrückung des Immunsystems zur Folge haben. Hierzu gehören beispielsweise Substanzen wie hoch dosierte Glukokortikoide, Ciclosporin, Tacrolimus oder Cyclophosphamid, aber auch eine systemische Polychemotherapie. Zu unterscheiden sind immunmodulierende/-supprimierende Substanzen, die zielgerichtet einzelne Zytokine, Signaltransduktionswege oder Zellfunktionen unterbinden, aber keine generalisierte Immunsuppression hervorrufen. Dazu gehören Biologika wie Tumornekrosefaktor(TNF)-, Interferon(IFN)- und Interleukin(IL)-Inhibitoren, Zellrezeptorblocker oder Januskinase(JAK)-Inhibitoren. Dennoch muss man bedenken, dass die Folgen einer gezielten Immunmodulation umfassender sein können, weil abhängige Aktivierungswege ebenso unterbunden werden können und/oder die Wirkung sehr lange anhalten kann (z. B. bei Anti-CD20-Antikörpern).

Im Folgenden wurde, je nach Substanzklasse, eine Einteilung der Grade der Immunsuppression vorgenommen, die eine Hilfe für die Einschätzung der verbleibenden Immunkapazität darstellen soll. Gegenüber der früheren Einteilung (Grad I–III) wurde Grad III nun in IIIa und IIIb unterteilt, weil besonders Grad IIIb auf langanhaltende (z. B. Anti-CD20-Antikörper) oder sehr schwere Immunsuppressionen hinweisen soll (z. B. unmittelbar nach Stammzelltransplantation [SZT] oder bis ca. 3 bis 6 Monate bei zellvermittelten Therapien – siehe Tab. 1).

**Tab. 1:** Schweregrade der Immunsuppression mit Beispielen. (Adaptiert nach [2])

**Grad-0- bis -I-Immunsuppression: Erkrankungen/Therapien ohne relevante Immunsuppression**	**Grad-0- bis -I-Immunsuppression unter folgenden Bedingungen**
*HIV-Infektion*	Personen mit CD4-T-Zellzahl ≥ 500/mm^3^ bzw. > 25 % bei Kindern
*Hämatoonkologische Erkrankungen*	> 3 Monate seit Ende der Immun‑/Chemotherapie (> 6 Monate bei Anti-CD20-Antikörpertherapie)
	Personen in Remission ohne Erhaltungstherapie
	SZT > 2 Jahre (ohne aktuelle immunsuppressive Therapie und ohne GvHD)
*Autoimmunerkrankungen (MS, RA, CED)*	Ohne immunsuppressive bzw. -modulierende Therapie
*Diabetes mellitus*	Bei guter Einstellung (< 6,5–7 % HbA1c)
*Kortisontherapie*	Kurzzeittherapie für < 2 Wochen; Prednisolon < 20 mg/d oder entsprechende Äquivalenzdosis
	Langzeittherapie: alternierende Tagestherapie (jeden 2. Tag < 20 mg), topische, inhalative, intraartikuläre oder intrabursale Applikation
*Immunmodulierende Therapie*	Mesalazin, Sulfasalazin, Hydroxychloroquin, Lenalidomid
**Grad-II-Immunsuppression: Erkrankungen/Therapien mit leichter bis mittelgradiger Immunsuppression**	**Grad-II-Immunsuppression unter folgenden Bedingungen**
*HIV-Infektion*	Asymptomatische Personen mit CD4-T-Zellzahl 200–499/mm^3^ bzw. 15–25 % bei Kindern
*Anatomische oder funktionelle Asplenie*	Siehe Tab. 16, anatomische Asplenie geringere Immunsuppression als funktionelle Asplenie mit Grunderkrankung
*Chronische Nierenerkrankung*	Allgemeine CKD-assoziierte Immundysfunktion durch chronisch-inflammatorische Immunsuppression; eGFR < 60 ml/min/1,73 m^2^ oder ein Nierenschaden > 3 Monate wird als chronischer Nierenschaden bezeichnet
*Chronische Lebererkrankung*	v. a. bei Zirrhose (Child-Pugh B und C) und anderen Formen der fortgeschrittenen Lebererkrankung/Fibrose
*Diabetes mellitus*	Bei fortgeschrittener Erkrankung (> 7–8 % HbA1c) und schlechtem Allgemeinzustand
*Komplementdefizienz (z. B. MBL-Defizienz)*	–
*Kortisontherapie*	Prednisolon < 20 mg/d oder entsprechende Äquivalenzdosis für > 2 Wochen bzw. regelmäßige Tagesdosis von < 20 mg/d
	Prednisolon > 20 mg/d oder entsprechende Äquivalenzdosis für < 2 Wochen; Verabreichung von Depotkortison
*Andere immunsuppressive Therapien*	Methotrexat ≤ 25 mg/Woche
	Azathioprin ≤ 150 mg/d
	6‑Mercaptopurin < 1,5 mg/kg/d
	Interferon‑α
	Integrin-Antagonisten (α4β7)
**Grad-IIIa-Immunsuppression: Erkrankungen/Therapien mit schwerer Immunsuppression**	**Grad-IIIa-Immunsuppression unter folgenden Bedingungen**
*Hämatoonkologische Erkrankungen*	Hämatoonkologische Neoplasien unter Chemotherapie (bis 3 Monate nach Therapieende und vor Remission)
	Chronisch-lymphatische Leukämie
*HIV-Infektion*	CD4-T-Zellzahl < 200/mm^3^ bzw. < 15 % bei Kindern
*Aplastische Anämie, MDS, Polyzytämie*	–
*Primäre Immundefekte*	(Siehe Kapitel 5.9)
*Solide Organtransplantation (moderat immunsupprimiert, z. B. Leber)*	Transplantation > 1 Jahr
*Stammzelltransplantation*	> 3 Monate nach autologer bzw. > 6 Monate nach allogener SZT bis ≤ 2 Jahre oder unter immunsuppressiver Therapie auch > 2 Jahre
*CAR-T-Zell-Therapie*	> 6 Monate nach CAR-T-Zell-Therapie bis Immunrekonstitution
*Rezente Strahlentherapie*	Bis max. 6 Wochen nach Therapieende bzw. bei Lymphopenie (< unterer Grenzwert)
*Chemotherapien*	Während Therapie und (3–)6 Monate nach Beendigung der Therapie
*Immunsuppressive Therapien; Wirkstoffe mit Interaktion mit Immunzellen*	Biologika, „small molecules“, zielgerichtete Therapien, Immuntherapien; z. B. hoch dosierte Glukokortikoide, IL-Inhibitoren, TKI, JAK-Inhibitoren
**Grad-IIIb-Immunsuppression: Erkrankungen/Therapien mit sehr schwerer/dauerhafter Immunsuppression**	
*Solide Organtransplantation (stark immunsupprimiert, z. B. Niere)*	Transplantation ≤ 1 Jahr
	Abstoßungstherapie bei GvHD
*B‑Zell-gerichtete/depletierende Therapien*	z. B. Anti-CD20- oder Anti-CD19-Antikörpertherapie
	Unter Therapie und bis zu 6–12 Monate nach Therapieende
*CAR-T-Zell-Therapie*	Bis 6 Monate nach Zelltherapie bzw. vor Immunrekonstitution
*Stammzelltransplantation*	< 3 Monate nach autologer bis < 6 Monate nach allogener SZT ± immunsuppressive Therapie ± GvHD

*CAR* „chimeric antigen receptor“, *CED* chronisch-entzündliche Darmerkrankung, *CKD* chronische Nierenerkrankung, *d* Tag, *GvHD* Graft-versus-host-Erkrankung, *HIV* humanes Immundefizienzvirus, *IL* Interleukin, *JAK* Januskinase, *MBL* mannosebindendes Lektin, *MDS* Myelodysplastisches Syndrom, *MS* Multiple Sklerose, *RA* rheumatoide Arthritis, *SZT* Stammzelltransplantation, *TKI* Tyrosinkinaseinhibitor, *v. a.* vor allem

### 2.2 Schweregrade der Immunsuppression

Allgemein betrachtet ergibt sich der zu erwartende Schweregrad einer Immunsuppression aus unterschiedlichen Parametern, wie der Indexerkrankung, der Art und Dauer der laufenden Behandlung sowie Vorbehandlung, dem Allgemeinzustand und dem Alter der betroffenen Person sowie dem Vorhandensein und der Art weiterer Erkrankungen [2]. Manche dieser Parameter sind nicht präzise bestimmbar oder ändern sich im Laufe der Zeit. So können vormals stark immunsuppressive Biologika durch Neu- oder Weiterentwicklungen deutlich günstigere Eigenschaften erwerben. Deshalb sollen die unten angeführten Schweregrade eher der Orientierung dienen, als validierte prädiktive Kategorien darstellen.

Es können drei Schweregrade der Immunsuppression unterschieden werden:

- **Grad 0–I** – keine Einschränkung: keine bzw. sehr geringe Immunsuppression oder Grunderkrankung mit potenzieller Immunmodulation;
- **Grad II** – limitierte Einschränkung: Erkrankungen/Therapien mit leichter bis mittelgradiger Immunsuppression;
- **Grad IIIa und IIIb** – schwere bis sehr schwere Einschränkung: Erkrankungen/Therapien mit hochgradiger (IIIa) bis dauerhafter oder vorübergehend sehr schwerer (IIIb) Immunsuppression.

Eine Einschätzung des zu erwartenden Schweregrads der Immunsuppression bei verschiedenen Erkrankungen bzw. Therapien bietet Tab. 1. Ihre konkrete Anwendung wird bei den jeweiligen Personengruppen weiter unten besprochen. In Tab. 7 sind unterschiedliche Infektionsrisikokategorien qualitativ beschrieben. Im Abschnitt 5 finden sich detaillierte Tabellen zu den einzelnen Substanzklassen.

### 2.3 Impffähigkeit in Abhängigkeit des Schweregrads der Immunsuppression

Eine grobe Übersicht über die Impffähigkeit in Abhängigkeit des Schweregrads der Immunsuppression gibt Tab. 2. Grundsätzlich können inaktivierte Impfstoffe bei allen Schweregraden der Immunsuppression eingesetzt werden. In Abschnitt 2.4 wird auf den optimalen Zeitpunkt der Impfungen in Relation zur Therapie eingegangen. Die Verabreichung von Lebendimpfstoffen ist auf die Grade I und II beschränkt. Das Impfansprechen kann ab Grad II jedoch reduziert sein und eine Impferfolgskontrolle ist empfohlen (Tab. 5).

**Tab. 2:** Impffähigkeit in Abhängigkeit des Schweregrads der Immunsuppression

**Grad 0–I**	**Grad II**	**Grad IIIa/IIIb**
*Keine Einschränkung*	*Limitierte Einschränkung*	*Schwere bis sehr schwere Einschränkung*
*Keine bzw. sehr geringe Immunsuppression oder Grunderkrankung mit potenzieller Immunmodulation*	*Erkrankungen/Therapien mit leichter bis mittelgradiger Immunsuppression*	*Erkrankungen/Therapien mit hochgradiger (IIIa) bis dauerhafter oder vorübergehend sehr schwerer (IIIb) Immunsuppression*
Inaktivierte Impfstoffe: **JA**	Inaktivierte Impfstoffe: **JA**	Inaktivierte Impfstoffe: **möglich**
Lebendimpfstoffe: **JA**	Lebendimpfstoffe: **möglich** (Nutzen-Risiko-Abschätzung)	Lebendimpfstoffe: **NEIN**^a^
Das Impfansprechen ist nicht reduziert	Das Impfansprechen kann reduziert sein und eine Impferfolgskontrolle ist möglich^b^	Das Impfansprechen ist meist reduziert und eine Impferfolgskontrolle ist empfohlen^b^

^a^ Wenn ein Lebendimpfstoff während einer Therapieunterbrechung oder nach Ende einer immunsuppressiven Therapie verabreicht werden soll, sind in Abhängigkeit von der Art der Therapie gewisse Intervalle zwischen Therapieende und Impfung zu beachten (siehe Abb. 3 und Abschnitt 6)

^b^ Serologische Impferfolgskontrollen/Titerkontrollen können nicht bei allen Impfungen routinemäßig durchgeführt werden (siehe Tab. 4 und 5)

Bei der Planung einer Behandlung mit zielgerichteten Therapien können eventuell auch die Websites des BASG und der EMA (siehe Quellen im Abschnitt 9) helfen, um die aktuellen Fachinformationen abzurufen und somit den Grad der Immunsuppression eines bestimmten Wirkstoffs abzuschätzen. Im Abschnitt 6 wird das praktische Vorgehen bei Immunsuppression erörtert.

### 2.4 Zeitliche Abstände zwischen Impfung und Immunsuppression

Prinzipiell können inaktivierte Impfstoffe zu allen Zeitpunkten unbedenklich appliziert werden, jedoch ist der Impferfolg maßgeblich abhängig von der Immunkompetenz der betroffenen Person. Um dies zu überprüfen, stehen für viele – aber nicht für alle Impfungen – serologische Impferfolgskontrollen („Titerkontrollen“) zur Verfügung (siehe Tab. 5), die frühestens 4 Wochen nach Impfung durchgeführt werden können/sollen.

**Abb. 2:**
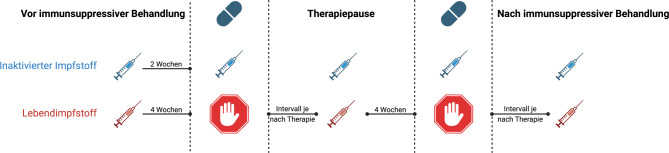
Überblick: zeitliches Impfvorgehen in Abhängigkeit von Impfstoffart und immunsuppressiver Therapie. *Tablette* immunsuppressive Therapie, *Spritze* Impfung. (Für Intervalle bei Lebendimpfstoffen bei Stammzelltransplantation siehe Abb. 3 bzw. Abschnitt 6)

Prinzipiell wird immer eine möglichst frühzeitige Impfversorgung – am besten vor Therapiebeginn – angestrebt. Bei Grad-III-Immunsuppression sind vor allem bei Lebendimpfstoffen unterschiedliche Abstände in Bezug auf die immunsuppressive Behandlung vor und nach Impfung einzuhalten (Abb. 2). Auf die Möglichkeiten für Impfungen vor, während und nach einer Therapie sowie während der Therapiepausen soll in diesem Abschnitt eingegangen werden.

#### 2.4.1 Vor Therapiebeginn

Idealerweise sollten alle Personen, die eine immunsuppressive Therapie erhalten, vor Beginn der Behandlung einen vollständigen Impfschutz entsprechend des österreichischen Impfplans [6] (siehe Abschnitt 9) aufweisen und fehlende Impfungen nachgeholt werden. Dabei sollen alle inaktivierten Impfstoffe bis spätestens 2 Wochen vor Therapiebeginn verabreicht werden, um ein optimales Impfansprechen sicherzustellen. Dennoch können im Einzelfall inaktivierte Impfstoffe auch bis kurz vor Therapiebeginn gegeben werden, wenn dieser rasch erfolgen muss (Tab. 3).

**Tab. 3:** Impfungen vor Therapiebeginn

	**Für optimales Impfansprechen – inaktivierte Impfstoffe bis 2 Wochen vor Therapiebeginn**	**Aus Sicherheitsgründen – Lebendimpfstoffe bis spätestens 4 Wochen vor Therapiebeginn**^**a**^
*WICHTIG: Umgebungsprophylaxe*	COVID-19	Masern-Mumps-Röteln
	Diphtherie-Tetanus-Pertussis, Poliomyelitis (DTPP)	Varizellen → Alternative: inaktivierter Herpes-Zoster-Impfstoff (besonders bei geplanter Organtransplantation)
	FSME bei Exposition	Influenzalebendimpfstoff → Alternative: inaktivierter Impfstoff
	*Haemophilus influenzae B *(HiB) bei Indikation	Für Reiseimpfungen siehe Abschnitt 8
	Hepatitis A bei Indikation	
	Hepatitis B	
	Herpes Zoster	
	HPV bei Indikation	
	Influenza (jährlich, saisonal)	
	Meningokokken bei Indikation	
	Pneumokokken	
	RSV	

*COVID-19* Coronavirus disease 2019, *FSME* Frühsommer-Meningoenzephalitis, *HPV* humanes Papillomavirus, *RSV* respiratorisches Synzytialvirus

^a^ In Einzelfällen, z. B. Ocrelizumab, bis zu 6 Wochen vor Therapiebeginn

Lebendimpfstoffe müssen bis spätestens 4 Wochen (in Einzelfällen, z. B. Ocrelizumab, bis 6 Wochen) vor Therapiebeginn verabreicht werden. Kurzfristiger vor Therapiebeginn bzw. bei bereits bestehender Immunsuppression ist bei Lebendimpfstoffen nicht mehr mit ausreichender Sicherheit zu rechnen. Unter Immunsuppression vermehren sich lebend-attenuierte Viren bzw. Bakterien stärker und können einen krankheitsähnlichen Verlauf induzieren. Es kann vermehrt zu Nebenwirkungen kommen und es gibt ein Risiko für eine Verschlechterung der zugrunde liegenden Krankheit sowie dass die Impfung keinen ausreichenden Schutz entfaltet ([7]; Tab. 3).

Wichtig ist in jedem Fall die altersgemäße Impfung aller Kontaktpersonen, der An- und Zugehörigen sowie Personen, die in medizinische oder soziale Betreuung involviert sind (Umgebungsprophylaxe), die im Wesentlichen den Empfehlungen des österreichischen Impfplans ([6]; siehe Abschnitt 9) folgen soll.

#### 2.4.2 Während einer immunsuppressiven Therapie

Während der immunsuppressiven Therapie (Grad III) sind **nur inaktivierte Impfstoffe möglich**. Während die Applikation des inaktivierten Impfstoffs unbedenklich ist, kann aber das Ansprechen in Anhängigkeit von der Therapie reduziert sein. Deshalb ist eine Impferfolgskontrolle (allerdings nicht bei allen Impfungen routinemäßig durchführbar) empfohlen (siehe Tab. 4 und 5).

**Tab. 4:** Inaktivierte Impfungen während einer immunsuppressiven Therapie und Impferfolgskontrolle

**Impfstoff**	**Impfapplikation**	**Impferfolgskontrolle (siehe Tab.** 5)
*COVID-19*	Möglich	Möglich
*DTPP*	Möglich	Möglich
*FSME*	Möglich	Möglich
*Hepatitis B/A*	Möglich	Möglich
*Herpes Zoster*	Möglich	Nicht bzw. eventuell möglich (VZV-Antikörper)^a^
*HiB*	Möglich	Möglich
*HPV*	Möglich	Nicht möglich
*Influenza (inaktiv)*	Möglich	Nicht möglich
*Meningokokken*	Möglich	Möglich
*Pneumokokken*	Möglich	Möglich
*RSV*	Möglich	Nicht möglich

*COVID-19* Coronavirus disease 2019, *DTPP* Diphtherie, Tetanus, Pertussis, Polio, *FSME* Frühsommer-Meningoenzephalitis, *HiB Haemophilus influenzae B, HPV* humanes Papillomavirus, *RSV* respiratorisches Synzytialvirus, *VZV* Varicella-Zoster-Virus

^a^ Kontrolle bei vorheriger bekannter Seronegativität

**Tab. 5:** Antikörperkontrollen zur Überprüfung des Impferfolgs. (Adaptiert nach [6])

**Impfung gegen**	**Methode**	**Akzeptierte Grenzwerte für positive Impfantwort**	**Kommentar**
COVID-19/SARS-CoV‑2^a^	NT oder NT-Korrelate	Keine international anerkannten Titer definiert	In Einzelfällen (z. B. bei unklarer immunologischer Reaktionsfähigkeit auf eine Impfung), frühestens 4 Wochen nach der COVID-19-Impfung zur Klärung, ob eine Immunantwort ausgelöst wurde
Diphtherie^a^	Liganden-Assay, wie z. B. ELISA	IgG ≥ 0,1 IE/ml	Schutzkorrelat definiert, Schutz und Schutzdauer interpretierbar
FSME^a^	Liganden-Assay, wie z. B. ELISA; NT	NT > 1:10	FSME-IgG-Antikörper nur aussagekräftig, wenn die FSME-Impfung der einzige Flaviviruskontakt war. Bei Impfung oder Kontakt mit anderen Flaviviren (z. B. Gelbfieber, Japan. Enzephalitis, Dengue) ist als Spezialdiagnostik ein FSME-NT zur Messung der funktionell aktiven Antikörper notwendig (Interpretation abhängig vom verwendeten NT-System)
Hepatitis B^a^ Hepatitis A^a^	Liganden-Assay, wie z. B. ELISA	Anti-HBs-Antikörper, Serokonversion: > 10 mIE/ml	Hepatitis B: Langzeitschutz bei Anti-HBs-Antikörper > 100 mIE/ml Hepatitis A: qualitative Bewertung laut verwendetem Testsystem
HiB	ELISA	IgG ≥ 0,15 µg/ml Anti-PRP	Vor und nach Impfung: Impfansprechen nachweisbar
Masern^a^	Liganden-Assay, wie z. B. ELISA; NT	IgG positiv (n. d.)	Bewertung abhängig vom verwendeten Testsystem
Meningokokken	Liganden-Assay, wie z. B. ELISA	n. d.	Schutz ist für jeden Serotyp vom Vorhandensein von Antikörpern gegen die jeweiligen spezifischen Kapselantigene abhängig. Für serumbakterizide Antikörper (hSBA) gilt 1:4 als Schutzgrenze (kommerziell nicht erhältlich) ELISA: vor/nach Impfung: Impfansprechen nachweisbar
Mumps^a^	Liganden-Assay, wie z. B. ELISA, NT	IgG-positiv (n. d.)	Bewertung abhängig vom verwendeten Testsystem
Pertussis^a^	Liganden-Assay, wie z. B. ELISA	n. d.	Beurteilbar ist nur Seronegativität (< Detektionslimit) bzw. Anstieg von Pertussis-spezifischen Antikörper vor/nach Impfung
Pneumokokken (Konjugatimpfstoff)	Liganden-Assay, wie z. B. ELISA	Positiv	Kommerzielle Kits weisen Antikörper gegen ein Gemisch unterschiedlicher Pneumokokkenserotypen nach – keine Aussage über einzelne Serotypen möglich; es kann ein Ansprechen auf eine Pneumokokkenimpfung mit Impferfolgskontrolle vor und 4 Wochen nach Impfung erfolgen. (OPA-spezifische Assays sind derzeit kommerziell nicht erhältlich)
Polio	NT	Positiv	Bewertung abhängig vom verwendeten Testsystem
Röteln^a^	Liganden-Assay, wie z. B. ELISA	Positiv > 10–15 IE/ml	Bewertung abhängig vom verwendeten Testsystem
Tetanus^a^	ELISA	IgG ≥ 0,1 IE/ml	Schutz und Schutzdauer interpretierbar
Tollwut	RFFIT, NT	> 0,5 IE/ml	Schutz bei über > 0,5 IE/ml nachweisbar
Varizellen^a^	Liganden-Assay, wie z. B. ELISA; NT	IgG positiv	Bewertung abhängig vom verwendeten Testsystem

*COVID-19* Coronavirus disease 2019, *ELISA* „enzyme-linked immuno sorbent assay“, *FSME* Frühsommer-Meningoenzephalitis, *HBs* „Hepatitis B surface antigen“, *HiB Haemophilus influenzae B, hSBA* bakterizider Antikörpertest mit Humanserum, *IE* internationale Einheiten, *IgG* Immunglobulin G, *n. d.* nicht definiert, *NT* Neutralisationstest, *OPA* Opsonophagozytose-Antikörper, *PRP* Polyribosylribitolphosphat, *RFFIT* „rapid fluorescent focus inhibition test“, *SARS-CoV‑2* „severe acute respiratory syndrome Coronavirus 2“

^a^ Impferfolgskontrollen für die gekennzeichneten Impfungen möglich

**Lebendimpfstoffe sind während einer immunsuppressiven Therapie prinzipiell kontraindiziert, Ausnahmen sind möglich **(siehe Abschnitt 2.4.4)**.**

#### 2.4.3 Nach einer immunsuppressiven Therapie

**Abb. 3:**
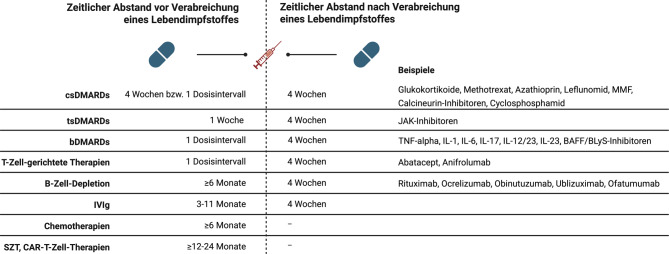
Intervalle zwischen Grad-III-immunsuppressiven Therapien und Lebendimpfstoffen. Die *Tablette* symbolisiert die immunsuppressive Therapie, die *Spritze* symbolisiert die Impfung. Für Substanzen, bei denen unterschiedliche Dosierungsintervalle zugelassen sind (wie bei bDMARDs oder T‑Zell-gerichteten Therapien), sollte das verabreichte Dosierungsintervall für das Pausieren vor Lebendimpfungen herangezogen werden. Bei Kindern mit autoinflammatorischen Erkrankungen oder systemischer juveniler idiopathischer Arthritis, bei denen das Infektionsrisiko bei Biologikabehandlung sehr hoch ist, können die Abstände zur Impfung ggf. verkürzt werden. *BAFF* B-Zell-aktivierender Faktor, *bDMARDs* biologische DMARDs, *BLyS* B-Lymphozytenstimulator, *CAR* „chimeric antigen receptor“, *csDMARDs* konventionelle synthetische DMARDs, *DMARDs* krankheitsmodifizierende Antirheumatika, *IL* Interleukin, *IVIg* intravenöses Immunglobulin, *JAK* Januskinase, *MMF* Mycophenolat mofetil, *SZT* Stammzelltransplantation, *TNF* Tumornekrosefaktor, *tsDMARDs* zielgerichtete synthetische DMARDs. *Asterisk* Bis zur Immunrekonstitution

Wenn nach einer immunsuppressiven Therapie geimpft wird, sollten für ein optimales Impfansprechen folgende Impfabstände eingehalten werden:

- bei inaktivierten Impfstoffen i. d. R. 2 bis 3 Monate nach Therapieende;
- für Lebendimpfstoffe gelten Abstände in Abhängigkeit vom Immunsuppressionsgrad der Therapie (siehe Abb. 3 bzw. Abschnitt 6).

Wurden während einer Therapie inaktivierte Impfstoffe verabreicht, empfiehlt sich, nach Therapieende eine Wiederholungsimpfung durchzuführen (siehe Abschnitt 6).

Eine Ausnahme zu dieser Regelung sind die saisonalen Impfungen (wie Influenza oder COVID-19), die unabhängig vom Therapieverlauf in der Saison gegeben werden sollten.

#### 2.4.4 Lebendimpfungen im Therapieintervall bei Grad-III-Immunsuppression

Lebendimpfstoffe können in einer stabilen Krankheitsphase bei möglichst geringer Immunsuppression bzw. zwischen den Therapiezyklen zwar verabreicht werden, allerdings ist dabei je nach Art der immunsuppressiven Therapie ein unterschiedliches Intervall bis zur Impfung sowie i. d. R. 4 Wochen zwischen Impfung und Wiederaufnahme der Behandlung einzuhalten (siehe beispielgebend Abb. 3; [8]; für Details siehe Abschnitt 6).

## 3 Immunstatus und Impferfolg

Personen mit Immunsuppression haben nicht nur ein erhöhtes Infektionsrisiko, sondern auch einen individuell sehr unterschiedlichen Impferfolg. Es gilt in jedem Fall, beim Erstgespräch mit den Betroffenen den aktuellen Impfstatus bzw. Immunschutz zu erheben, den Grad der Immunsuppression entsprechend der Krankheit/Therapie einzuschätzen oder ggf. zu bestimmen sowie bei den nötigen Impfungen einen Zeitplan zu erstellen (siehe Abschnitt 2.4). Für die individuelle Bestimmung der Immunsuppression bzw. zur Abschätzung und Kontrolle des individuellen Impfansprechens stehen folgende Methoden zur Verfügung:´

**Abb. 4:**
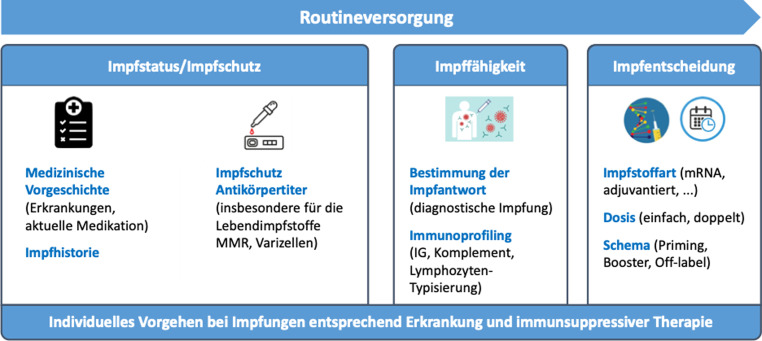
Fahrplan für Impfprogramme für Risikopatienten. *IG* Immunglobulin, *MMR* Masern-Mumps-Röteln, *mRNA* Messenger-Ribonukleinsäure

- Überprüfung des aktuellen Immun‑/Impfstatus (Antikörper‑/Titerbestimmung),
- Überprüfung auf Impfansprechen (diagnostische Impfung) und
- Erhebung von Qualität und Quantität der Immunzellen (Immunoprofiling; Abb. 4).

### 3.1 Erhebung des aktuellen Impfstatus/Immunschutzes bei Lebendimpfungen

Grundsätzlich sollte immer darauf geachtet werden, einen Impfschutz gemäß dem aktuellen österreichischen Impfplan [6] aufrecht zu erhalten (siehe Abschnitt 9). Ist geplant, einer Person eine immunsuppressive Therapie zu verabreichen, sollten fehlende Impfungen vor Beginn der immunsuppressiven Therapie vervollständigt werden.

Die Grundlage für die Erhebung des aktuellen Immunschutzes ist primär der Impfpass. Ist der Impfpass nicht auffindbar, sollten die Informationen zu früher durchgeführten Impfungen aus ärztlichen Unterlagen ermittelt werden. Bei fehlender oder unvollständiger Dokumentation von Impfungen empfiehlt es sich, von fehlenden Impfungen auszugehen, da anamnestische Angaben zu bisherigen Impfungen oder durchgemachten Krankheiten oft unzuverlässig sind [6, 9].

Alternativ können auch serologische Antikörperüberprüfungen durchgeführt werden. **Besonders wichtig ist es, den Immunschutz gegenüber Erkrankungen, gegen die mit Lebendimpfstoffen geimpft wird, zu überprüfen, da diese unter laufender immunsuppressiver Therapie kontraindiziert sind. Dies betrifft: Masern, Mumps, Röteln und Varizellen**. Für die Impferfolgskontrolle stehen unterschiedliche Testsysteme zur Verfügung (Liganden-Assay, „enzyme-linked immuno sorbent assay“ [ELISA] oder ein Neutralisationstest [NT]; siehe Tab. 5).

Alle durchgeführten Impfungen und ggf. zuletzt im Impfpass vermerkte und noch gültige Impfungen sollen in den **elektronischen Impfpass** eingetragen werden.

Beim Vorliegen einer Immunsuppression (wegen primären Immundefekts, immunmodulierender Erkrankung oder laufender immunsuppressiver Therapie) soll der Grad der Immunsuppression bestimmt bzw. abgeschätzt und die Möglichkeit von Impfungen überprüft werden.

#### Off-Label-Use

Im Rahmen der Impfungen immunsupprimierter Patienten können Anwendungen außerhalb der Fachinformation gegeben werden („Off-Label-Use“). Dieser ist prinzipiell immer möglich, sofern eine klare Indikation und eine strenge Nutzen-Risiko-Analyse vorangegangen ist bzw. – wenn vorhanden – beruhend auf publizierten Daten oder theoretischen Überlegungen. In diesen Fällen muss eine genaue Aufklärung erfolgen und eine Zustimmung des Patienten vorliegen und dokumentiert sein.

Weitere Details zum Off-Label-Use finden sich im österreichischen Impfplan (siehe Abschnitt 9; [6]).

### 3.2 Bestimmung der Impffähigkeit/Immunkompetenz von Personen mit Immunsuppression mittels diagnostischer Impfung

Zur Abschätzung des Grades des Immundefekts oder der Immunsuppression und deren Auswirkungen auf die Impfantwort können diagnostische/anamnestische Impfungen mit inaktivierten Impfstoffen durchgeführt werden [6]. Eine Impfung mit Diphtherie/Tetanus-Toxoid hat sich für diesen Zweck bewährt, da der Diphtherie/Tetanus-Impftiter als aussagekräftiger Surrogatmarker für die Beurteilung des Impfansprechens gegenüber Proteinantigenen gilt [5]. Die Kontrolle des Impftiters sollte vor und etwa 4 bis 8 Wochen nach erfolgter Impfung/Impfserie erfolgen, wobei ein mindestens 2‑facher bis idealerweise 4‑facher Anstieg des antigenspezifischen Immunglobulin-G(IgG)-Spiegels eine Immunkompetenz nahelegt.

**Wichtig:** Bei schweren Immundefekten, Agammaglobulinämie sowie direkt nach Stammzelltransplantation oder schweren B‑ und T‑Zell-supprimierenden Medikationen ist eine diagnostische Impfung jedoch i. d. R. nicht sinnvoll [2].

### 3.3 Immunoprofiling unter Therapie

Mithilfe von Immunoprofiling kann der Zustand des Immunsystems einer Person zu einem bestimmten Zeitpunkt bestimmt werden. Das Immunoprofiling umfasst den humoralen und zellulären Status des Immunsystems und kann möglicherweise eine Abschätzung des Impfansprechens erleichtern [10]. Dies fußt auf der Messung der Anzahl, Differenzierung und Funktion der Immunzellen (T- und B‑Lymphozyten, natürliche Killerzellen [NK-Zellen] und deren relevante Subsets, Granulozyten und Monozyten) wie auch der Zusammensetzung und Konzentration von Immunglobulinen, Faktoren und Aktivität des Komplementsystems sowie ggf. des Zytokinmilieus im Serum. Verringerungen in Quantität und Qualität von Immunzellpopulationen oder zirkulierenden Immunproteinen oder in Einzelfällen weiterführende Analysen der Immunkompetenz und Proliferations‑/Differenzierungsaktivität von Immunzellsubsets (Reduktion von Proliferationsaktivität nach unspezifischer und spezifischer Stimulation, Zytokinproduktion, ggf. Vorhandensein von Anti-Zytokin-Autoantikörpern etc.) können Hinweise über den Schweregrad der Immunsuppression liefern. Derartige Untersuchungen sind besonders hilfreich nach Behandlung mit B‑Zell-depletierenden Substanzen oder bei dauerhafter immunsuppressiver Therapie.

### 3.4 Titerbestimmung zur Impferfolgskontrolle

Serologische Antikörperbestimmungen („Titerkontrollen“) werden vor allem durchgeführt, um den Impferfolg zu überprüfen, besonders wenn unklar ist, ob eine ausreichende Immunkompetenz vorliegt.

Man muss bei der Überprüfung der Immunantwort zwischen Immunogenität (Höhe der Antikörperspiegel) und Impfschutz unterschieden. Ein Anstieg der erregerspezifischen IgG-Antikörper allein bedeutet nicht zwangsläufig einen tatsächlichen Schutz vor Infektion/Erkrankung. Die Antikörperspiegel zeigen, dass prinzipiell ein Kontakt (durch Infektion oder Impfung) mit einem Erreger stattgefunden hat. Ein tatsächlicher Schutz ist nur dann nachweisbar, wenn der Nachweis funktionell aktiver, neutralisierender Antikörper durch eine Korrelation der Antikörperantwort mit der tatsächlichen Schutzwirkung (durch In-vitro-Assays oder in klinischen Studien) bestätigt wurde [2]. Entsprechende Antikörpertiter können dann mithilfe spezifischer Neutralisationstests in zertifizierten Labors bestimmt werden. Als Beispiel: Frühsommer-Meningoenzephalitis(FSME)-IgG-Antikörper sagen nur eingeschränkt etwas über den Schutzzustand aus, denn nach Kontakt mit anderen Flaviviren ist der Test aufgrund kreuzreaktiver Antikörper nicht aussagekräftig. Für den Nachweis eines vorliegenden FSME-Schutzes muss ein Neutralisationstest durchgeführt werden. Das Ergebnis liefert den Nachweis eines aufrechten Schutzes, jedoch ist eine genaue Auskunft über die Schutzdauer (besonders bei immunsupprimierten Personen) schwer möglich.

Einen Überblick über die Impfungen, bei denen eine Impferfolgskontrolle im Sinne einer Überprüfung des Schutzes oder der Schutzdauer sinnvoll sein kann und welche Methode dafür geeignet ist, gibt Tab. 5. In Abschnitt 2.3 zur Impffähigkeit von Personen mit Immunsuppression sowie im Abschnitt 6 zu den konkreten Impfempfehlungen wird ebenfalls auf die Impferfolgskontrolle eingegangen.

### 3.5 Immunsystem, Impfung und therapieinduzierte Immunsuppression

**Abb. 5:**
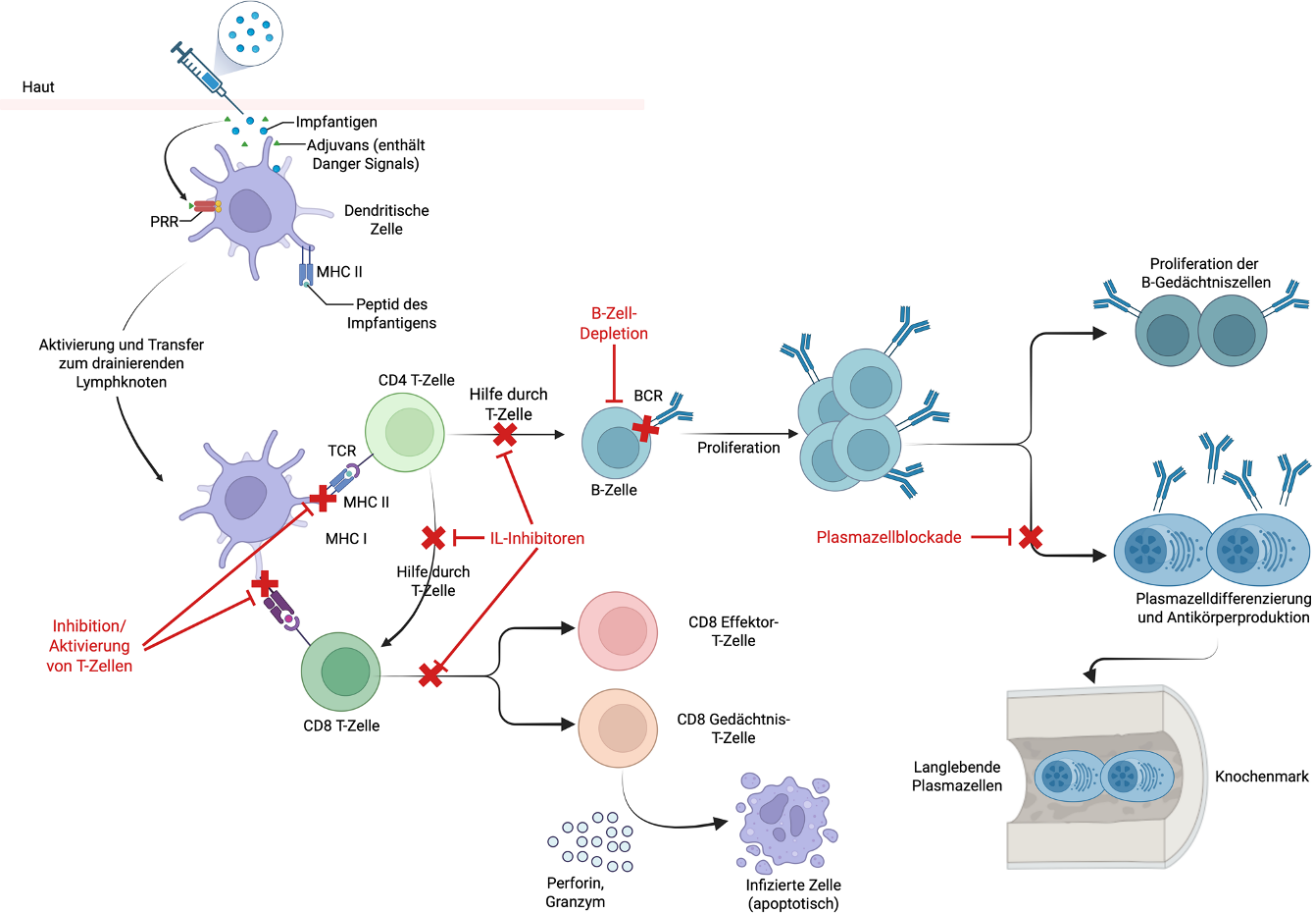
Ausbildung einer Immunantwort auf einen Impfstoff und deren Hemmung durch immunsuppressive/-modulierende Therapien. *BCR* B-Zell-Rezeptor, *MHC* Haupthistokompatibilitätskomplex, *PRR* Mustererkennungsrezeptor, *TCR* T-Zell-Rezeptor. (Abbildung adaptiert nach [11])

In Abb. 5 wird ein kurzer Überblick über die Induktion/Inhibition einer Impfantwort gegeben. Nach der Applikation (i. d. R. intramuskulär oder subkutan) wird das Impfantigen von den dendritischen Zellen an der Impfstelle aufgenommen, die durch die Gefahrensignale (Danger-Signale) im Adjuvans über Mustererkennungsrezeptoren (PRRs) aktiviert und dann zum drainierenden (ableitenden) Lymphknoten transportiert werden (Abb. 5). Dort werden die CD4- und CD8-T-Zellen über ihren T‑Zell-Rezeptor (TCR) durch die Präsentation von Peptiden des Impfantigens über die Haupthistokompatibilitätskomplex(MHC)-Moleküle I und II auf der dendritischen Zelle aktiviert. In Kombination mit der Signalübertragung (durch lösliches Antigen) über den B‑Zell-Rezeptor (BCR) treiben die T‑Zellen mittels Ausschüttung von Zytokinen die B‑Zell-Proliferation und Differenzierung im Lymphknoten voran. Hier führt die T‑Zell-abhängige B‑Zell-Entwicklung zu einer Reifung der Antikörperantwort, um die Antikörperaffinität zu erhöhen und verschiedene Antikörperisotypen auszubilden. Kurzlebige Plasmazellen generieren innerhalb von 2 Wochen einen Anstieg impfstoffspezifischer Antikörper. Das Immungedächtnis wird durch die Bildung von Gedächtniszellen ausgebildet, wobei hinsichtlich der durch die Impfung induzierten schützenden Antikörper besonders den B‑Gedächtniszellen eine wichtige Rolle zukommt. Diese sind durch wiederholten Antigenkontakt für die Formierung langlebiger Plasmazellen verantwortlich, die über Jahrzehnte hinweg Antikörper produzieren können und in Knochenmarknischen einwandern. CD8-Gedächtnis-T-Zellen können sich schnell vermehren, wenn sie erneut auf einen Krankheitserreger treffen, und CD8-Effektor-T-Zellen sind wichtig für die Eliminierung infizierter Zellen [11].

Je nach der Art des Wirkstoffs ist der Effekt auf das Immunsystem entweder sehr generalisiert oder zielgerichtet auf die Interaktion von Immunzellen, auf bestimmte Zellpopulationen oder auf die von ihnen produzierten Botenstoffe (Zytokine).

Zielgerichtete Therapien setzen auf molekularer Ebene an, um die Signaltransduktion zur Bildung bestimmter Zytokine zu verhindern. Im Rahmen von Krebstherapien werden aber auch Substanzen eingesetzt, die die Immunzellaktivität fördern, um Krebszellen zu eliminieren. Diese Checkpoint-Inhibitoren haben demnach keine immunsuppressive Wirkung, können aber in Kombination mit Impfungen eventuell zu verstärkten Reaktionen führen. Diese Reaktionen können von verstärkten immunassoziierten unerwünschten Wirkungen bis zu einem möglichen Überlebensvorteil bei Tumorpatienten reichen, sind aber noch nicht ausreichend untersucht [12, 13] und bedürfen weiterer Evaluierung [14]. Zur Influenza- und COVID-19-Impfung gibt es Daten, die zeigen, dass ihre Verabreichung während einer Behandlung mit Checkpoint-Inhibitoren wirksam und gut verträglich sind [15, 16].

Durch die jeweiligen immunsuppressiven Therapien wird aber auch das Risiko für bestimmte Infektionen erhöht. Dieses erhöhte Infektionsrisiko bewirkt die Notwendigkeit der jeweiligen Impfversorgung. Im Abschnitt 5 werden die gängigsten Therapien bei diversen Erkrankungen und die damit assoziierten Infektionsrisiken und Impfindikationen beschrieben.

## 4 Grundlagen zu Impfstoffen – Update

### 4.1 Arten von Impfstoffen

**Abb. 6:**
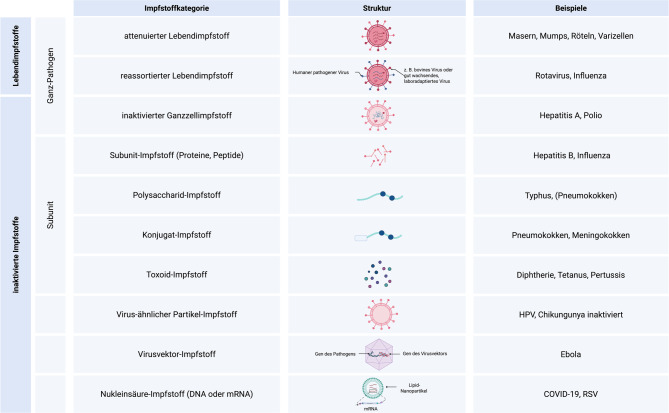
Impfstofftypen und Beispiele. (Adaptiert nach [11, 17].) Anmerkung: Alle Nichtlebendimpfstoffe werden zur Vereinfachung als inaktivierte Impfstoffe bezeichnet. Darunter fallen Impfstoffe, deren zugrunde liegender Erreger durch z. B. Formaldehyd inaktiviert wurde, sowie all jene, die in der Abbildung als Subunit-Impfstoffe gelistet sind. *COVID-19* „Coronavirus disease 2019“, *DNA* Desoxyribonukleinsäure, *HPV* humanes Papillomavirus, *mRNA* Messenger-Ribonukleinsäure, *RSV* humanes respiratorisches Synzytialvirus

Grundsätzlich gibt es zwei Kategorien von Impfstoffen: Lebendimpfstoffe und Nichtlebendimpfstoffe. Im vorliegenden Dokument wird aufgrund der gängigen Bezeichnung der Begriff „inaktivierte Impfstoffe“ stellvertretend für alle Nichtlebendimpfstoffe verwendet (Abb. 6). Aus historischen Gründen kommt auch der Begriff „Totimpfstoffe“ häufig in diesem Zusammenhang vor. In der Literatur [2, 5, 11, 17] gibt es umfassende Beschreibungen der verfügbaren Impfstofftypen und ihrer Wirkungsweisen. Mit Verweis auf diese Publikationen soll hier nur kurz das Wesentliche mit Fokus auf neue Impfstofftypen zusammengefasst werden.

#### 4.1.1 Lebendimpfstoffe

Sie enthalten vermehrungsfähige Pathogene (Viren oder Bakterien). Diese sind im Vergleich zu Wildtyp-Pathogenen allerdings abgeschwächt (attenuiert) und können bei gesunden Personen keine Krankheit hervorrufen. **Bei immunsupprimierten Personen kann eine Impfung mit einem Lebendimpfstoff allerdings sehr wohl zur Erkrankung führen.** Die Attenuierung eines Pathogens erfolgt, indem die Viren (Bakterien werden aufgrund ihrer genetischen Komplexität nur sehr selten attenuiert) über viele Zyklen in vitro in nichthumanen Zellen repliziert werden und dadurch die Vermehrungsfähigkeit im Menschen stark abgeschwächt wird. Sie verlieren durch diesen Prozess aber nicht ihre Antigenität für den Menschen. Viren können auch über viele Zyklen in vitro bei geringer Temperatur (z. B. 25 °C) repliziert werden und so die Fähigkeit verlieren, sich bei der üblichen menschlichen Köpertemperatur von 36/37 °C zu vermehren ([17]; siehe Abb. 6). Weiters gibt es reassortierte Lebendimpfstoffe, bei denen Viren gekreuzt („reassortiert“) werden. Beispiele sind Influenzaimpfstoffe, wo der aktuelle Virusstamm mit einem laboradaptierten, gut wachsenden Virusstamm gekreuzt wird [18], oder der Rotavirusimpfstoff, wo ein humanes und ein bovines Virus reassortiert werden [19]. Schließlich gibt es auch nichtreplikationsfähige Lebendimpfstoffe, die bedenkenlos unter Immunsuppression appliziert werden können, z. B. Mpox.

Die Immunantwort auf Lebendimpfstoffe ist der einer natürlichen Infektion am ähnlichsten, weil sie eine humorale und eine zelluläre Immunantwort induzieren, die auch beim Wildtyp-Virus/-Bakterium induziert wird [2, 5, 11, 17]. Beispiele für attenuierte Lebendimpfstoffe sind die Masern-Mumps-Röteln-, die Varizellen-, oder die Gelbfieberimpfung. Unter den Rotavirusimpfstoffen gibt es sowohl attenuierte wie auch reassortierte Lebendimpfstoffe. Häufig reichen eine, bis maximal zwei Applikationen für einen dauerhaften, fast lebenslangen, Impfschutz aus. Eine Ausnahme stellt der Influenzalebendimpfstoff dar, der aufgrund der jährlichen Antigenshifts der Influenzaviren keinen lebenslangen Schutz bietet.

Es ist wichtig zu beachten, dass Lebendimpfstoffe bei bestehender schwerer Immunsuppression nicht angewendet werden dürfen und ihre Applikation möglichst vor Eintreten der Immunsuppression, also vor Beginn einer immunsuppressiven Behandlung, erfolgen sollte. Diese Thematik wird in den einzelnen Abschnitten genauer besprochen.

#### 4.1.2 Inaktivierte/nicht-lebend-Impfstoffe

In der Literatur werden häufig die Begriffe „inaktivierte Impfstoffe“ und „Totimpfstoffe“ gleichbedeutend verwendet. Als inaktivierte Impfstoffe werden in diesem Dokument inaktivierte Erreger (Ganzkeimimpfstoffe) oder nur immunogene Bestandteile von Erregern (Subunit‑/Teilpartikelimpfstoffe, Proteinimpfstoffe, Polysaccharidimpfstoffe, Konjugatimpfstoffe, Toxoidimpfstoffe) oder deren genetische Bauinformationen, wie z. B. Messenger-Ribonukleinsäure(mRNA)-Impfstoffe mit und ohne Vakzinvektoren zusammengefasst [2, 5, 11, 17].

Inaktivierte Impfstoffe induzieren vorwiegend eine humorale, jedoch nur eine partielle zelluläre Immunantwort und müssen nach einer Grundimmunisierung meist regelmäßig aufgefrischt bzw. geboostert werden. Meist werden auch Adjuvanzien zugesetzt, um die immunogene (und zellvermittelte) Wirkung der Impfantigene zu verstärken [5]. Beispiele für inaktivierte Impfstoffe sind der inaktivierte Polio‑, FSME-, Hepatitis-A-Impfstoff, oder der rekombinante Teilantigenimpfstoff gegen Hepatitis B bzw. die Toxoidimpfstoffe gegen Tetanus und Diphtherie. Ebenfalls unter die inaktivierten Impfstoffe fallen die COVID-19-Impfstoffe.

Besonderes Augenmerk soll hier auf die mRNA-Impfstoffe, die Subunit-Impfstoffe und die Vektorimpfstoffe gelegt werden. Als weiterführende Literatur für Impfstofftypen im Allgemeinen, ihre Herstellung und Wirkungsweisen seien [11] und [17] empfohlen.

##### 4.1.2.1 mRNA-Impfstoffe

An der Technologie der mRNA-basierten Wirkstoffe wird seit über 30 Jahren geforscht [20, 21]. Im Zuge der COVID-19-Pandemie wurden jedoch durch die gemeinsame Anstrengung der Forschungsgemeinschaft, der Regierungen, der Zulassungsbehörden und der Pharmaindustrie innerhalb weniger Monate nach der erstmaligen Sequenzierung von SARS-CoV‑2 Impfstoffe auf Basis der mRNA-Technologie entwickelt, klinisch geprüft und zugelassen [20]. Mittlerweile gibt es weitere Impfstoffe, die auf Basis dieser Technologie entwickelt wurden und werden, z. B. gegen humanes respiratorisches Synzytialvirus (RSV) oder Influenza oder COVID-19-Influenza-Kombinationsimpfstoffe oder Ebola.

**Abb. 7:**
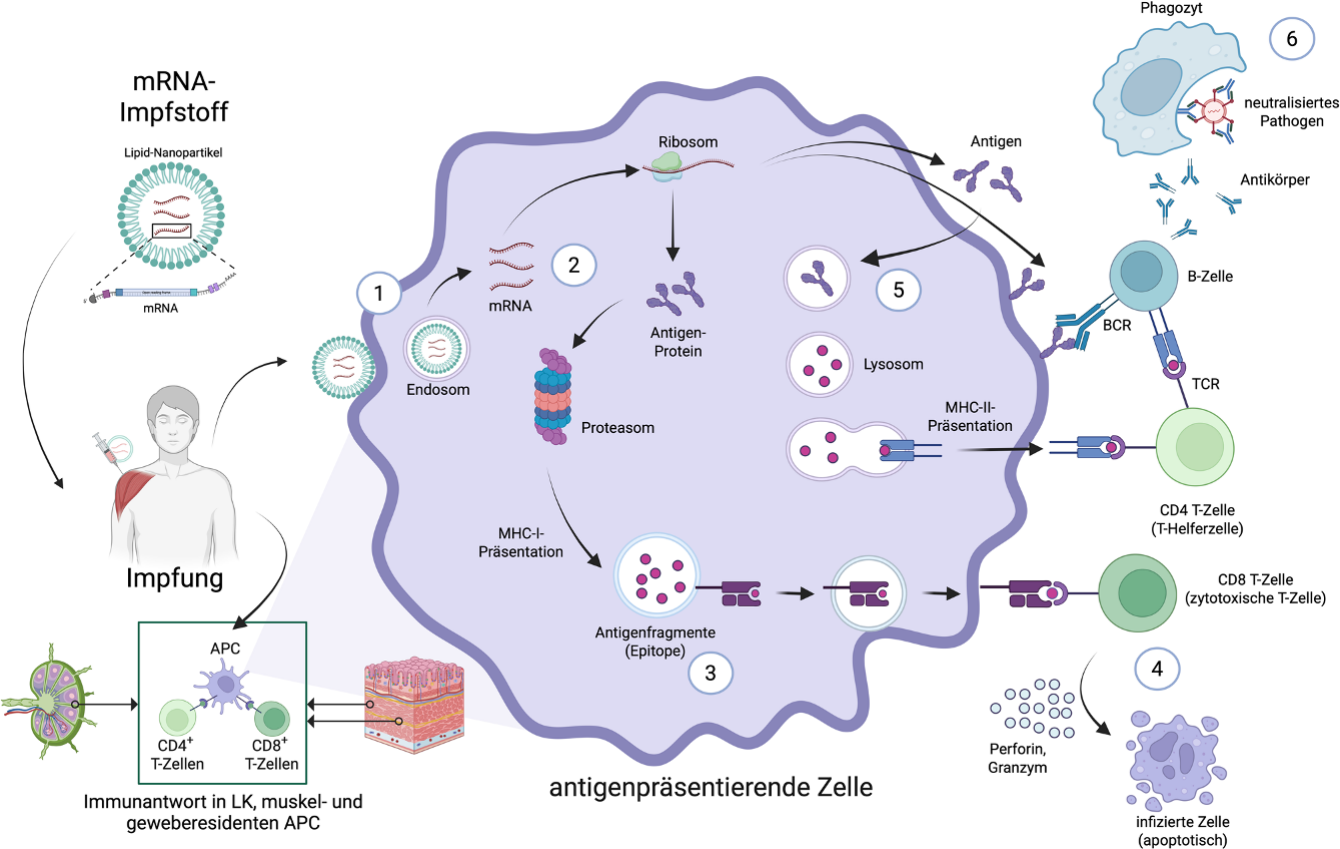
Wirkweise der mRNA-Impfstoffe. (Adaptiert nach [20, 22].) Die Schritte 1–6 werden im Text erklärt. *APC* antigenpräsentierende Zelle, *BCR* B-Zell-Rezeptor, *LK* Lymphknoten, *MHC* Haupthistokompatibilitätskomplex, *mRNA* Messenger-Ribonukleinsäure, *TCR* T-Zell-Rezeptor

Im Prinzip bestehen mRNA-Impfstoffe aus einer synthetischen mRNA und einem Vehikel, das der relativ großen, negativ geladenen mRNA erlaubt, die anionische Zellmembran zu passieren, ohne vorher durch Immunzellen aufgenommen und abgebaut zu werden. Grundsätzlich eignen sich lipidbasierte Nanopartikel (LNP), polymere Nanopartikel und kationische Nanoemulsionen als Vehikel, wobei COVID-19-Impfstoffe auf LNP zurückgreifen. Diese LNP bilden sich, indem Lipide mit der mRNA gemischt werden und diese in Vesikel einschließen [20, 21]. mRNA-Impfstoffe bilden eine Immunität gegen die von ihnen kodierten Virusbestandteile aus, indem nach Transfektion antigenpräsentierender Zellen virusspezifische Proteine gebildet und dann den B‑ und T‑Zellen präsentiert werden. Der genaue Wirkmechanismus von mRNA-Impfstoffen ist in Abb. 7 dargestellt. mRNA-Impfstoffe können Muskelzellen, geweberesidente, antigenpräsentierende Zellen (APC) in der Nähe der Injektionsstelle sowie lymphknotenresidente Zellen transfizieren, was zu einer Aktivierung von B‑ und T‑Zellen führt. Die injizierten mRNA-LNP werden von den antigenpräsentierenden Zellen endozytiert (Schritt 1 in Abb. 7). Im Zytosol wird die mRNA vom Ribosom in Antigenprotein translatiert (Schritt 2). Dieses Antigenprotein kann das Immunsystem auf verschiedene Weise stimulieren. Das von der mRNA kodierte Impfantigen kann zur Gänze auf der Zelloberfläche exprimiert und dadurch von B‑Zellen erkannt werden. Abgesehen davon kann das Antigenprotein durch den Proteasomenkomplex in kleinere Fragmente zerlegt werden, welche den zytotoxischen T‑Zellen (CD8^+^) durch MHC-Klasse-I-Proteine auf der Zelloberfläche präsentiert werden (Schritt 3). Die dadurch aktivierten zytotoxischen T‑Zellen sezernieren zytolytische Moleküle wie Perforin und Granzym und töten damit infizierte Zellen ab (Schritt 4). Zusätzlich können die Proteinantigene auf der Zelloberfläche durch MHC-Klasse-II-Proteine den T‑Helferzellen (CD4+) präsentiert werden (Schritt 5). T‑Helferzellen erleichtern die Beseitigung zirkulierender Krankheitserreger, indem sie B‑Zellen zur Produktion neutralisierender Antikörper anregen und Fresszellen, wie Makrophagen, durch sezernierte Zytokine aktivieren (Schritt 6).

An dieser Stelle sei erwähnt, dass bei den SARS-CoV-2-mRNA-Impfstoffen eine Mutation im Spikeprotein eingebaut wurde (doppelte Prolinmutation). Dadurch wird dieses – im Unterschied zum nativen Spikeprotein des Virus – im Präfusionszustand stabilisiert und es bleibt somit nach Bindung an der Zelloberfläche verankert, um die Bindung an die Angiotensin-Converting-Enzyme-2(ACE2)-Rezeptoren in verschiedenen Geweben/Organen zu verhindern. Dadurch sollen Komplikationen wie beim nativen Protein verhindert oder stark reduziert werden [20].

Prinzipiell zeigen die mRNA-Impfstoffe aufgrund der Aktivierungswege des angeborenen und erworbenen Immunsystems eine höhere Reaktogenität als herkömmliche Impfstoffe. Gerade für Personen mit geschwächtem Immunsystem kann dies aber vorteilhaft für den Aufbau eines Impfschutzes sein.

##### 4.1.2.2 Subunit-Impfstoffe

Zu den Subunit-Impfstoffen zählen immunogene Proteine, Oberflächenpolysaccharide bzw. ihre Proteinkonjugate sowie Virusmembranimpfstoffe (siehe Abb. 6; [11, 23]).

Anstatt ganzer Viruspartikel werden bei den Subunit-Impfstoffen immunogene Partikel eingesetzt. Diese immunogenen Partikel können gereinigte oder rekombinante Proteine oder Polysaccharide der Virusoberfläche sein. Manche Impfstoffe nutzen aber auch die leere Virushülle ohne entsprechendes Genmaterial. Diese ahmt die Virusstruktur nach, kann sich aber nicht replizieren [23]. Impfstoffe, welche auf den Oberflächenpolysacchariden von Bakterien basieren, werden meist in Form von Protein-Polysaccharid-Konjugaten verabreicht, um neben der Antikörperantwort auch eine T‑Zell-Antwort zu erreichen [11].

Subunit-Impfstoffe gibt es beispielsweise gegen SARS-CoV‑2 (enthält das Spikeprotein an der Oberfläche des SARS-CoV-2) oder gegen Meningokokken B (enthält ein Vesikel der äußeren Membran von *Neisseria meningitidis* der Gruppe B). Auch einige der Influenzaimpfstoffe werden zu den Subunit-Impfstoffen gezählt (die meisten sind aber Spaltimpfstoffe).

Als erster rekombinanter Subunit-Impfstoff kam der Hepatitis-B-Impfstoff auf den Markt.

##### 4.1.2.3 Hochvalente Konjugatimpfstoffe

Typische Konjugatimpfstoffe sind z. B. Pneumokokkenimpfstoffe. Da das Impfantigen aus Polysaccharidantigenen der Kapsel von den unterschiedlichen Pneumokokkenserotypen stammt und diese für kleine Kinder (bis 2 Jahre) nicht immunogen sind, werden solche „T-Zell-unabhängigen“ Antigene durch Konjugation an Trägerproteine (zumeist Tetanustoxoid oder [modifiziertes] Diphtherietoxoid) zugängig für T‑Zellen gemacht. Dadurch kann eine T‑Zell-abhängige Immunantwort mit entsprechender B‑Zell-Aktivierung erfolgen. Aufgrund der sich laufend verändernden epidemiologischen Situation (u. a. induziert durch sogenannte Replacement-Phänomene im Zuge von Impfprogrammen) ist es nötig, die Impfstoffe entsprechend den Veränderungen der Erreger in Form neuer hochvalenter Impfstoffe anzupassen.

##### 4.1.2.4 Vektorimpfstoffe

Vektorimpfstoffe haben während der COVID-19-Pandemie eine breitere Bekanntheit erlangt. Vektorimpfstoffe bestehen aus einem rekombinanten, für den Menschen harmlosen Virus, dem Vektor, in dessen Genom auch Genabschnitte des Impfantigens eingebracht wurden. Als Vektorviren werden häufig Adenoviren verwendet, da sie über lange Zeit gut untersucht wurden und z. B. auch bei Gentherapien zum Einsatz kommen [24]. Erste wissenschaftliche Arbeiten dazu stammen aus den 1960er-Jahren [25]. Nach der Impfung wird die Virus-DNA in den Zellkern transportiert und nach entsprechender Transkription bilden die transfizierten körpereigenen Zellen das Virusantigen aus und triggern damit die entsprechende Immunantwort. Es gibt replizierende und nichtreplizierende Vektorimpfstoffe, wobei sich bei ersteren die Vektorviren in der geimpften Person replizieren und weitere Zellen für eine zusätzliche Impfantigenproduktion transfizieren können, bei zweiteren produzieren nur die durch die Impfung transfizierten Zellen das Impfantigen [26]. Vektorimpfstoffe führen zu einer endogenen Impfantigenproduktion und bilden daher eine humorale und eine zelluläre Immunantwort aus [26].

Die rekombinanten COVID-19-Vektorimpfstoffe Vaxzevria (ChAdOx1-S) und JCovden (Ad26.COV2-S) zeigten eine deutlich erhöhte Nebenwirkungsrate. Da diese Impfstoffe keine 2P-Mutationen im S‑Protein eingebaut hatten, könnte dies einer der Gründe für die erhöhte Nebenwirkungsrate gewesen sein [27]. Mittlerweile wurde die Zulassung dieser Vektorimpfstoffe von der Europäischen Kommission auf Antrag der Zulassungsinhaber aus kommerziellen Gründen aufgehoben [28, 29].

Gam-COVID-Vac Lyo, ein russischer Vektorimpfstoff, der auch als „Sputnik“ bekannt ist, der aber nie bei der EMA zur Zulassung eingereicht wurde, nutzt zwei nicht vermehrungsfähige Adenoviren: einen Adenovirusvektor auf der Basis des Adenovirus vom Typ 26 (AD 26) und einen auf Basis des Adenovirus Typ 5 (AD 5), in welche jeweils das Spikeprotein-Gen des Coronavirus integriert wurde. Dabei wird der erste Impfstoff als „Primer“ verwendet, der zweite als „Booster“. Dieser Impfstoff wurde in einigen europäischen Ländern (z. B. in Russland, in Ungarn, der Slowakei und Serbien) angewendet.

### 4.2 Adjuvanzien-Update

Adjuvanzien sind Zusatzstoffe, welche die Immunantwort auf das Impfantigen verstärken. Das können sie auf verschiedene Weise bewirken: Zum einen helfen sie, das Impfantigen in den Organismus einzubringen und dort seine Bioverfügbarkeit durch eine Art Depoteffekt zu verlängern. Zum anderen können sie auch als Immunstimulanzien wirken [30]. Immunstimulanzien können in den Zellen Danger-Signale (Gefahrensignalmoleküle) in Form von Entzündungssignalen induzieren, die andere Zellen anlocken können [31]. Toll-like-Rezeptoren (TLR) und Mustererkennungsrezeptoren auf den antigenpräsentierenden Zellen können sowohl endogene als auch exogene Danger-Signal-Moleküle erkennen, womit eine Reifung und Aktivierung der antigenpräsentierenden Zellen angeregt werden. Daraufhin werden Antigensignale und kostimulierende Signale ausgesandt und somit die Immunantwort des adaptiven Immunsystems deutlich stärker angeregt als ohne Adjuvanzien [30].

Zu den am häufigsten verwendeten Adjuvanzien zählen Aluminiumsalze, die seit mehr als hundert Jahren zum Einsatz kommen. Die neue Generation von Adjuvanzien, die neben der Antikörperbildung auch die zelluläre Abwehr stärken, sind liposombasierte Adjuvanzien, Öl-in-Wasser-Emulsionen und solche, die über die Aktivierung von TLR wirken (TLR‑4 oder 9). Beispiele (die meist eine Kombination verschiedener Formen darstellen) sind MF59, Adjuvanssystem (AS) 01, AS03 und AS04 sowie CpG 1018 und LNP ([30]; Tab. 6).

**Tab. 6:** Adjuvanzien (weltweit in Verwendung). (Adaptiert nach [32, 33])

**Adjuvans**	**Zusammensetzung**	**Adjuvierte Impfstoffe (Beispiele)**
Aluminiumsalze	Ein oder mehrere der folgenden Aluminiumsalze: Aluminiumkaliumphosphat (Alum), amorphes Aluminiumhydroxyphosphatsulfat, Aluminiumhydroxid (AAHS), Aluminiumphosphat	Diphtherie, Tetanus, Hepatitis A, Hepatitis B, HPV, Japanische Enzephalitis, FSME, Meningokokken B, Pneumokokken
AS01_B/E_	Liposomale Formulierung aus MPL und QS21	Malaria, Herpes Zoster, RSV
AS03	Öl-in-Wasser-Emulsion aus (D,L)-α-Tocopherol, Squalen und Tween 80	Influenza (pandemisch)
AS04	Monophosphoryl-Lipid‑A (MPL) auf einem Aluminiumsalz	Hepatitis B, HPV
MF59	Öl-in-Wasser-Emulsion aus Squalen, Span 85 und Tween 80	Influenza (saisonal und pandemisch)
CpG 1018	22-mer-Oligonukleotid-Sequenz mit Cytosin-Phosphat-Guanin(CpG)-Motiven	Hepatitis B
CpG 7909	24-mer-Oligonukleotid-Sequenz mit Cytosin-Phosphat-Guanin(CpG)-Motiven	Anthrax
Virosome	Virusähnliche Partikel bestehend aus synthetischen Phospholipiden, Virusproteinen und Membranlipiden	(Ehemals Influenza, Hepatitis A)
Lipid-basierte Nanopartikel	Verschiedenste Kompositionen basierend auf pegylierten Lipiden, Phospholipiden und Cholesterin	COVID-19-mRNA
SQBA	Öl-in-Wasser-Emulsion aus Squalen, Polysorbat 80, Sorbitantrioleat, Natriumcitrat, Zitronensäure	COVID-19-Proteinimpfstoff
Matrix‑M	Nanopartikelformulierung aus verschiedenen Saponinfraktionen (Matrix‑A, Matrix-C)	COVID-19, Malaria, Influenza

*AS* Adjuvanssystem, *COVID-19* „Coronavirus disease 2019“, *CpG* Cytosin-Phosphat-Guanin, *FSME* Frühsommer-Meningoenzephalitis, *HPV* humanes Papillomavirus, *MF* Mikrofluidisierung, *MPL* Monophosphoryl-Lipid-A, *mRNA* Messenger-Ribonukleinsäure, *QS* *Quillaja saponaria*, *RSV* humanes respiratorisches Synzytialvirus, *SQBA* Squalen-basiertes Adjuvans

### 4.3 Impfstoff-Update

Seit der Publikation der österreichischen Impfempfehlungen bei Immunsuppression im Jahr 2016 [2] wurden wesentliche neue Impfstoffe entwickelt und auf dem europäischen Markt zugelassen. Mit Verweis auf den österreichischen Impfplan, das BASG und die EMA (für Links siehe Abschnitt 9), werden hier nur die neuen bzw. bisher nicht behandelten Impfstoffe im Detail besprochen.

#### 4.3.1 COVID-19

Für die COVID-19-Impfung sind gemäß der Listung auf der EMA-Website (siehe Abschnitt 9) aktuell zwei Impfstoffarten zugelassen: mRNA-Impfstoffe und proteinbasierte Subunit-Impfstoffe. mRNA-Impfstoffe gegen COVID-19 enthalten eine Art Bauanleitung für die Bildung des Spikeproteins, eines Oberflächenproteins des SARS-CoV‑2. Die aktuell zugelassenen Subunit-Impfstoff enthalten das Spikeprotein selbst oder die rezeptorbindende Domäne als Fusionsdimer. Diese Antigene lösen eine Immunreaktion aus und immunisieren somit die geimpfte Person [34]. Proteinbasierte Impfstoffe bestehen aus viralen Antigenfragmenten, welche ebenfalls eine Immunantwort auslösen und dadurch eine Immunität generieren [35]. Weiters gab es für COVID-19 noch mehrere Vektorimpfstoffe, deren Zulassungen jedoch auf Antrag der Zulassungsinhaber aus kommerziellen Gründen aufgehoben wurden [28, 29]. Diese nutzten einen Adenovirusvektor, um die Codon-optimierten DNA-Sequenzen des Spikeproteins des SARS-CoV‑2 in die Wirtszellen der geimpften Person einzuschleusen und nach entsprechender Transkription in mRNA und Translation in reifes S‑Protein eine Immunantwort auszulösen [36].

Die Impfung mit beiden Arten von COVID-19-Impfstoffen bewirkt vorwiegend eine Reduktion des Schweregrads der COVID-19-Erkrankung und eine Verhinderung von Hospitalisierung und Tod. Ein Schutz vor Infektion ist zwar in den Anfangsmonaten nach Impfung möglich, aber wie bei den meisten Impfstoffen gegen respiratorische Infektionen aufgrund fehlender mukosaler Immunitätsinduktion limitiert. Die COVID-19-Impfstoffe werden an wichtige zirkulierende Varianten des SARS-CoV‑2 angepasst. Daten zur Impfeffektivität mit saisonal angepassten Impfstoffen in den vergangenen Saisonen haben gezeigt, dass eine erneute Impfung einen Nutzen für die geimpfte Person bringt – unabhängig vom vorbestehenden Status hinsichtlich durchgemachter Infektion und/oder Impfung. Dieser bewegt sich bei 40–50 % Risikoreduktion bezüglich symptomatischer Erkrankung, liegt in den ersten beiden Monaten nach der Impfung etwas höher bezüglich der Vermeidung von Hospitalisierungen und nimmt danach langsam ab [37–44].

Gemäß dem österreichischen Impfplan [6] ist eine COVID-19-Impfung besonders bei Personen mit Immundefekten, Immundefizienz oder immunsupprimierender Therapie (z. B. HIV-Infektion, Organ- oder Stammzelltransplantation, Autoimmunerkrankungen) indiziert [42–44].

##### Impfschema COVID-19-Impfstoffe

Routinemäßige Auffrischung (saisonal einmal/Jahr) für alle Personen mit Immunsuppression und/oder Alter ≥ 60 Jahre. Ein Abstand zur letzten Exposition (Infektion oder Impfung) von mindestens (4–)6 Monaten sollte eingehalten werden.

Für genaue Impfschemata und aktuelle Empfehlungen wird auf den jeweils aktuellen österreichischen Impfplan verwiesen (siehe Abschnitt 9).

#### 4.3.2 Respiratorisches Synzytialvirus (RSV)

Da das Risiko für schwere Verläufe einer RSV-Infektion bei Früh- und Neugeborenen besonders hoch ist, wird routinemäßig die passive Immunisierung von Säuglingen in der 1. Saison empfohlen und bei erhöhter Anfälligkeit bis zum 24. Lebensmonat. Aktuell sind drei monoklonale Antikörper, Palivizumab [45], Nirsevimab [46] und Clesrovimab zugelassen [47]. Die Anwendung der monoklonalen Antikörper gegen RSV bei immunsupprimierten Erwachsenen ist zum jetzigen Zeitpunkt nicht vorgesehen.

Im Erwachsenenalter ist besonders bei Personen mit Grunderkrankungen und/oder Immunsuppression bei RSV-Infektionen mit schweren Krankheitsverläufen zu rechnen [48]. Daher ist eine RSV-Impfung für diese Personen empfohlen. Gegen RSV sind aktuell drei Impfstoffe ab dem 18. Lebensjahr zugelassen: Arexvy [49], Abrysvo [50], mRESVIA [51]. Die Anwendung dieser Impfstoffe sollte gemäß offiziellen Empfehlungen erfolgen.

Arexvy ist ein monovalenter adjuvierter Subunit-Impfstoff [49]. Abrysvo ist ein für die beiden alternierend auftretenden Virusstämme (A/B) bivalenter, nichtadjuvierter Subunit-Impfstoff, der für Personen ≥ 18 Jahren sowie zur Impfung von Schwangeren in der 24. bis 36. Schwangerschaftswoche zugelassen ist [50]. mRESVIA ist ein mRNA-basierter Impfstoff, der für alle Personen ≥ 18 Jahren zugelassen ist [51]. Die mRNA, die in mRESVIA eingesetzt wird, kodiert für das in der Präfusionskonformation stabilisierte RSV-A-Glykoprotein F. mRESVIA verwendet den Hilfsstoff SM-102, ein basisches Lipid und Transfektionsreagens, welches die mRNA in die Zellen einschleust [51, 52]. SM-102 wurde erstmals im Zuge der Zulassung des COVID-19-Impfstoffs Spikevax klinisch untersucht und in der EU zugelassen [53].

Studien an Erwachsenen im Alter von ≥ 18 Jahren mit erhöhtem RSV-Risiko (Personen mit chronischen Erkrankungen, Diabetes oder Raucher etc.) zeigen, dass sowohl Immunität als auch Reaktogenität des Impfstoffs vergleichbar sind mit jenen bei Erwachsenen ≥ 60 Jahren [54].

##### Impfschema RSV-Impfstoffe

Die aktive Immunisierung gegen RSV wird routinemäßig ab dem vollendeten 60. Lebensjahr und bei Personen mit Immunsuppression ≥ 18 Jahren mit einer einmaligen Impfung empfohlen, bevorzugt vor der RSV-Saison, die meist von Oktober bis März/April andauert. Während die Schutzdauer bei nichtimmunsupprimierten Personen derzeit für 3 Saisonen (Arexvy) angegeben wird, ist die Dauer des Impfschutzes bei Immunsupprimierten unklar.

Für genaue Impfschemata und aktuelle Empfehlungen wird auf den jeweils aktuellen österreichischen Impfplan verwiesen. Laufende Studien mit immunsupprimierten Personen werden klären, wie lange (eine oder mehrere Saisonen) mit einem Schutz zu rechnen ist und wann Folgeimpfungen benötigt werden. Das BASG und die EMA-Website bieten eine stets aktuelle Übersicht aller Impfstoffe (siehe Abschnitt 9).

#### 4.3.3 Influenza

Influenzaimpfstoffe werden jährlich auf Empfehlung der Weltgesundheitsorganisation (WHO) und der EMA an die rezent zirkulierenden Virusstämme angepasst. Nachdem die Impfstoffherstellung in etwa 6 Monate dauert, werden die in der Südhalbkugel zirkulierenden Virusstämme als Orientierung genommen, die Empfehlungen der WHO für die Nord- und Südhalbkugel sind aber nicht zwingend identisch.

Bei Personen mit schwerer Immunsuppression bzw. immunsupprimierenden Therapien kann altersunabhängig (abweichend von der Fachinformation [FI]) eine Impfung mit einem adjuvierten, inaktivierten Impfstoff (z. B. Fluad, zugelassen ab dem vollendeten 50. Lebensjahr) oder einem inaktivierten Hochdosisimpfstoff (z. B. Efluelda, zugelassen ab dem vollendeten 60. Lebensjahr) verabreicht werden. Ggf. kann nach frühestens 4 Wochen einmal mit einem nichtadjuvierten Influenzaimpfstoff aufgefrischt werden. Sind Fluad und Efluelda nicht verfügbar, sollen auch die nichtadjuvierten inaktivierten Impfstoffe als zwei Applikationen im Mindestabstand von 4 Wochen verabreicht werden. In jedem Fall ist auch das Umfeld von Risikopersonen konsequent zu impfen [6]. Eine Koapplikation mit der COVID-19-Impfung ist (im Einzelfall/prinzipiell) möglich, allerdings ist auf die Saisonalität zu achten.

Der nasale Lebendimpfstoff (Fluenz), der in Europa von 2 bis 18 Jahren zugelassen ist, ist für alle immunsupprimierten Personen kontraindiziert.

##### Impfschema Influenzaimpfstoffe

Die aktive Immunisierung mit adjuviertem- oder Hochdosisimpfstoff (für Personen mit Immunsuppression bzw. immunsupprimierenden Therapien) wird einmal pro Saison durchgeführt. Alternativ kann ein nichtadjuvierter Standardimpfstoff zweimal pro Saison verabreicht werden. Die Influenzaimpfung sollte bevorzugt Ende Oktober/Anfang November, aber nicht früher, gegeben werden, um einen Schutz möglichst während der ganzen Influenzasaison zu halten.

**Wichtig:** Auf Umgebungsprophylaxe achten!

#### 4.3.4 Herpes Zoster

Gegen Herpes Zoster ist von der EMA ein inaktivierter Impfstoff zugelassen: der rekombinante, adjuvierte Subunit-Impfstoff Shingrix ([55]; Anmerkung: Der Lebendimpfstoff Zostavax ist nicht mehr empfohlen, verfügbar oder zugelassen). Im österreichischen Impfplan [6] wird aufgrund der hohen, persistierenden Wirksamkeit [56] der Impfstoff Shingrix empfohlen, welcher auch bei immungeschwächten Personen lang andauernd wirksam und sicher ist. Sogar bei schwer immunsupprimierten Personen (nach hämatopoetischer Stammzelltransplantation oder bösartigen hämatoonkologischen Erkrankungen) konnte gezeigt werden, dass robuste humorale und zelluläre Immunantworten mehr als 12 Monate persistieren [56] und eine Wirksamkeit (Vakzineffizienz, VE) von 68 % zum Schutz vor Herpes Zoster bei Personen mit autologer Stammzelltransplantation über einen Beobachtungszeitraum von 21 Monaten und bei Personen mit hämatoonkologischen Erkrankungen eine VE von 87 % über 11 Monate vorliegt [57, 58]. Bei Gesunden liegen die Wirksamkeitsdaten derzeit für 11 Jahre vor [59].

Im österreichischen Impfplan wird eingeräumt, dass auch varizellenseronegative, immungeschwächte Personen (off-label) mit dem inaktivierten Herpes-Zoster-Impfstoff geimpft werden können [6]. Die Impfung ist bei diesen initial varizellenseronegativen Personen immunogen. In Hochrisikogruppen sollten jedoch Impferfolgskontrollen in Betracht gezogen werden, um Personen mit keinem oder nur geringem humoralem Ansprechen zu identifizieren [60]. Daten zum Infektionsschutz vor Varizellenerstinfektion durch eine Shingrix-Impfung liegen aber nicht vor.

Grundsätzlich wird allen Personen ab dem vollendeten 60. Lebensjahr die Herpes-Zoster-Impfung empfohlen. Bei Personengruppen mit besonders hohem Infektionsrisiko bzw. Risiko eines schweren Verlaufs (schwere Grunderkrankungen und/oder schwere Immunsuppression) wird die Impfung ab 18 Jahren empfohlen. Eine Koapplikation mit anderen Impfstoffen ist möglich, es sollte jedoch die erhöhte Reaktogenität des Impfstoffes im Aufklärungsgespräch berücksichtigt werden.

Für genaue Impfschemata und aktuelle Empfehlungen wird auf den jeweils aktuellen österreichischen Impfplan verwiesen. Das BASG und die EMA-Website bieten eine stets aktuelle Übersicht aller Impfstoffe (siehe Abschnitt 9).

##### Impfschema Herpes-Zoster-Impfstoffe

Bei Risikopersonen ≥ 18 Jahren (Gesunde ab dem vollendeten 60. Lebensjahr) werden zwei Dosen in einem Abstand von mindestens 2 Monaten (2–6 Monate möglich) verabreicht. Bei Immundefizienz/Immunsuppression kann die 2. Dosis bereits 1 bis 2 Monate nach der 1. Dosis verabreicht werden.

Nach einer durchgemachten Herpes-Zoster-Reaktivierung ist bei Immunkompetenten ein Abstand von jedenfalls 6 Monaten zur Impfung einzuhalten. Bei Personen mit deutlich erhöhtem Rezidivrisiko für Herpes Zoster kann bereits 3 Monate nach der Erkrankung eine Impfung in Betracht gezogen werden [61].

#### 4.3.5 Meningokokken

Meningokokkenimpfstoffe sind i. d. R. spezifisch für bestimmte Serogruppen der Meningokokken. Es gibt Impfstoffe gegen Meningokokken der Serogruppe B, der Serogruppe C sowie kombinierte Impfstoffe gegen die Serogruppen A, C, W und Y. Der kurzzeitig zugelassene, pentavalente Impfstoff gegen die Serogruppen A, B, C, W und Y wurde von der Firma zurückgezogen [62].

Im Zusammenhang mit Immunsuppression sind Meningokokkenimpfungen aufgrund eines erhöhten Infektionsrisikos und der Gefahr schwerer und äußerst schneller Verläufe bei folgenden Risikogruppen empfohlen:

- bei Personen mit angeborenen oder erworbenen T- und/oder B‑Zell-Defekten (z. B. HIV oder Hypogammaglobulinämie);
- bei schwerer T‑Zell- und B‑Zell-Immunsuppressiva‑/Biologika-Therapie (z. B. Anti-CD20-Antikörper);
- Komplement‑/Properdin-/MBL-Mangel;
- bei Therapie mit/vor Anti-C5(Komplement)-Antikörpern (z. B. Eculizumab) bzw. „small molecule inhibitors“ (SMIs) gegen C3 (Pegcetacoplan) bzw. Faktor B (Iptacopan);
- Bei anatomischer oder funktioneller Asplenie, Splenektomie;
- Cochlea-Implantat und Liquorfistel.

Für genaue Impfschemata und aktuelle Empfehlungen wird auf den jeweils aktuellen österreichischen Impfplan verwiesen. Das BASG und die EMA-Website bieten eine stets aktuelle Übersicht aller Impfstoffe (siehe Abschnitt 9).

#### 4.3.6 Pneumokokken

Gegen Pneumokokken stehen unterschiedlich valente konjugierte Vakzinen (PCV 13, 15, 20, 21) und eine Polysaccharidvakzine (PPV23) zur Verfügung. In Österreich ist der 15-valente PCV15-Impfstoff im kostenfreien Kinderimpfprogramm enthalten (allgemein bis zum 2. Geburtstag und bei spezieller Indikation bis zum 5. Geburtstag). **Für Erwachsene ab dem vollendeten 60. Lebensjahr ist aktuell eine Impfung mit dem PCV21-Impfstoff im Impfplan** vorgesehen, auch wenn frühere Impfungen mit anderen Impfstoffen (PCV13/PCV15/PCV20 gefolgt von PPV23) erfolgt sind. Der empfohlene Abstand zu früheren Impfungen ist frühestens 1–6 Jahre. PCV21 ist nun auch für Kinder im Alter von 2–18 Jahren zugelassen zur Auffrischung nach einer pädiatrischen Grundimmunisierung mit konjugierten Pneumokokkenimpfstoffen [63].

Darüber hinaus liegt in folgenden Situationen ein erhöhtes Risiko und somit eine Indikation für eine Pneumokokkenimpfung vor:

- funktionelle oder anatomische Asplenie (Sichelzellanämie, andere schwere Hämoglobinopathien, angeborene oder erworbene Asplenie);
- Immundefekte wie z. B. Hypogammaglobulinämie, Komplement- und Properdin‑/MBL-Defizienz;
- sekundäre Immunsuppressionen, wie HIV-Infektion oder medikamentös induzierte Immunsuppressionen;
- vor und nach Organtransplantationen, vor Beginn und während einer immunsuppressiven Therapie (insbesondere Therapie mit Biologika);
- nach Stammzelltransplantation (autolog und allogen) oder chimärer Antigenrezeptor(CAR)-basierter Immuntherapie (z. B. CAR-T-Zell-Therapien);
- onkologische Erkrankungen (bes. Lungenkarzinome) und hämatoonkologische Erkrankungen.

##### Impfschema Pneumokokkenimpfstoffe

Bei den oben genannten Indikation ist das folgende sequenzielle Impfschema empfohlen:

**Kinder und Jugendliche bis 18 Jahre:**

- Ab dem vollendeten 2. Lebensjahr: PCV21 frühestens 8 Wochen nach abgeschlossener pädiatrischer Grundimmunisierung.
- Bis zum vollendeten 18. Lebensjahr: sequenzielle Impfung alle 6 Jahre seit der letzten Pneumokokkenimpfung mit PCV15 oder PCV20, nach 8 Wochen gefolgt von PCV21.
- Bei spezieller Indikation (z. B. Asplenie): sequenzielle Impfung alle 6 Jahre (PCV15 oder PCV20 und PCV21 nach 8 Wochen).
- Bis zum vollendeten 18. Lebensjahr nach Stammzelltherapie oder CAR-basierter Therapie: Grundimmunisierung im 3 + 1 Schema mit 3 Dosen PCV15 oder PCV20 im Abstand von jeweils 4 Wochen, gefolgt von 1 Dosis PCV21 nach 12 Monaten.

**Erwachsene ≥** **18 Jahren:**

- Ab dem vollendeten 18. Lebensjahr: Auffrischung mit PCV21 im Abstand von 6 Jahren nach der letzten Impfserie.
- Bei Cochlea-Implantation und bei Planung einer immunsuppressiven Therapie sollte möglichst früh eine Impfung mit PCV21 erfolgen.
- Personen **unter immunsuppressiver Therapie, Asplenie, Komplementdefekt: sequenzielle Impfung mit PCV20 und nach 8 Wochen PCV21.** Der Grund für diese Empfehlung liegt darin, dass die immunsupprimierten Personen nicht nur schlechter auf die Impfung ansprechen, sondern auch die zuvor aufgebaute Immunität verloren geht/abgeschwächt wird. Es ist davon auszugehen, dass weitere Impfungen für diese Personen empfohlen werden, dies richtet sich nach zukünftigen Daten.
- **Personen nach Stammzelltherapie oder CAR-basierter Therapie:** Grundimmunisierung im 3 + 1 Schema mit 3 Dosen PCV20 im Abstand von jeweils 4 Wochen, gefolgt von 1 Dosis PCV21 12 Monate später.

Das BASG und die EMA-Website bieten eine stets aktuelle Übersicht aller Impfstoffe. Für genaue Impfschemata und aktuelle Empfehlungen wird auf den jeweils aktuellen österreichischen Impfplan verwiesen (siehe Abschnitt 9).

## 5 Therapie-Update spezifischer Fachgebiete, Infektionsrisiko und Impfindikation

Dieses Kapitel beleuchtet das Infektionsrisiko durch die jeweiligen Erkrankungen und die damit verbundenen Therapien. Auf Basis dieses Infektionsrisikos ergibt sich die Indikation für die jeweils notwendigen Impfungen.

**Tab. 7:** Qualitative Beschreibung der Infektionsrisikokategorien

**Infektionsrisikokategorie**	**Definition**
*Niedrig*	Kein oder kaum messbares Infektionsrisiko
*Moderat*	Leicht erhöhtes Infektionsrisiko im Vergleich zu Gesunden oder Placebo
*Hoch*	Deutlich erhöhtes Infektionsrisiko mit möglicher Hospitalisierung
*Sehr hoch*	Stark erhöhtes Risiko mit möglicher Hospitalisierung oder Todesfolge, oft bei Kombinationstherapien

### 5.1 Rheumatologie

**Abb. 8:**
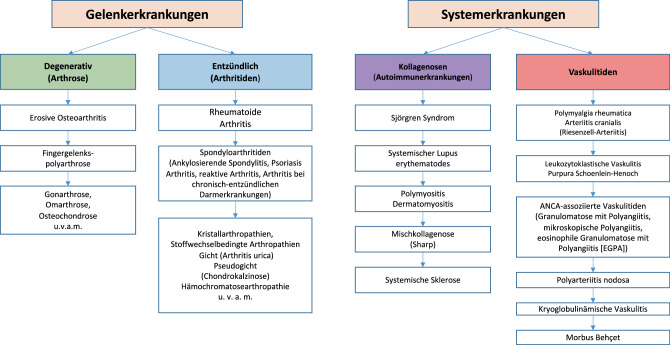
Einteilung von Autoimmunerkrankungen des rheumatischen Formenkreises. *ANCA* anti-Neutrophile zytoplasmatische Antikörper, *u.* *v.* *a.* *m.* und vieles andere mehr

Die Rheumatologie deckt ein breites Spektrum an entzündlichen und degenerativen Gelenkserkrankungen, systemische Autoimmunerkrankungen wie Kollagenosen bis zu entzündlichen Gefäßerkrankungen (Vaskulitiden) ab (Abb. 8).

Das Spektrum der Behandlungsmöglichkeiten ist entsprechend groß und wird im Falle der rheumatoiden Arthritis (RA) – abgesehen von Glukokortikoiden – in unterschiedliche Kategorien krankheitsmodifizierender Antirheumatika (DMARDs) unterteilt: in konventionelle synthetische csDMARDs, wie Methotrexat, Sulfasalazin, Leflunomid und Hydroxychloroquin, in biologische bDMARDS, wozu zahlreiche Zytokininhibitoren, aber auch B‑Zell- und T‑Zell-spezifische Antikörper gehören, und in zielgerichtete synthetische tsDMARDs. Zu dieser Gruppe gehören vorwiegend JAK-Inhibitoren und Tyrosinkinaseinhibitoren. Als Erstlinientherapien kommen die csDMARDs, vor allem Methotrexat, zum Einsatz [64, 65]. Auch Kortikosteroide können aufgrund ihrer entzündungshemmenden Wirkung kurzfristig als Erstlinientherapie eingesetzt werden [64].

In der zweiten Therapielinie folgen Kombinationen aus csDMARDs mit bDMARDs und zunehmend JAK-Inhibitoren [64–66]. In jüngster Zeit wird auch an neuen Konzepten, wie CAR-T-Zellen, die zielgerichtet auf B‑Zellen wirken, aber auch am Einsatz bispezifischer Antikörper gearbeitet [67].

Therapien gegen Kollagenosen, wie dem systemischen Lupus erythematodes (SLE), unterscheiden sich je nach Organbeteiligung, insbesondere bei renaler Beteiligung. Bei nichtrenalem Lupus erythematodes kommen vorwiegend folgende Medikamente zum Einsatz: Hydroxychloroquin, Azathioprin, Mycophenolat mofetil (MMF) sowie Biologika wie Belimumab (Blys/BAFF-Inhibitor), Anifrolumab (IFN-1-Inhibitor) oder Rituximab (Anti-CD20-Antikörper).

Bei einer Lupus-Nephritis kommen zusätzlich zu einer Basistherapie mit Hydroxychloroquin eine Monotherapie mit MMF, Kombinationen (Multi-targeted-Therapien) aus MMF mit Belimumab oder aus MMF mit einem Calcineurininhibitor zum Einsatz. Daneben können niedrig oder hoch dosiertes Cyclophosphamid als Einleitungstherapie, gefolgt von einer Erhaltungstherapie (MMF oder Azathioprin oder Multi-target-Therapien) verabreicht werden [65].

Personen mit rheumatoider Arthritis haben per se aufgrund der Erkrankung ein erhöhtes Infektionsrisiko. Darüber hinaus besteht eine deutliche Korrelation zwischen der Aktivität der Erkrankung, Komorbiditäten, der Art der immunmodulierenden Therapien und der Infektionsrate [68, 69]. Moderne zielgerichtete Therapien sind mit einem erhöhten Risiko für schwere Infektionen verbunden, jedoch hängt dieses sehr von der Wirkungsweise des Therapeutikums ab. Während beispielsweise TNF-Inhibitoren mit einem erhöhten Risiko zur Reaktivierung oder Neuinfektion mit Mykobakterien (v. a. *M. tuberculosis*) vergesellschaftet sind, steht bei den JAK-Inhibitoren das erhöhte Herpes-Zoster-Risiko im Vordergrund. Häufigkeit/Schweregrad von Infektionen, wie Pneumonie, Haut- und Schleimhautinfektionen oder Herpes Zoster sind bei Verabreichung von JAK-Inhibitoren deutlich höher als bei Interleukininhibitoren [70].

Bei Psoriasis-Arthritis und axialer wie periphererer Spondyloarthritis (SpA) werden mittlerweile vermehrt Biologikatherapien wie IL-17- und IL-23-Inhibitoren eingesetzt, die aber mit einem geringeren Infektionsrisiko als TNF- oder JAK-Inhibitoren verbunden sind [71]. Auch das Impfansprechen zeigt sich hier weniger beeinflusst [72].

Während hoch dosierter Glukokortikoidtherapien (> 20 mg/d, > 2 Wochen) kommt es zu einem deutlich erhöhten Risiko für opportunistische Infektionen [73], wie *Pneumocystis-jirovecii*-Pneumonien, Pilzinfektionen oder Sepsis (Tab. 8), besonders bei Kombinationstherapien mit JAK-Inhibitoren.

**Tab. 8:** Infektionsrisiko durch/mit Therapien in der Rheumatologie [64, 65, 68, 74, 75]

**Substanzgruppe**	**Beispiele**	**ISP-Grad**	**Infektionsrisiko**	**Besondere Risiken/Hinweise**
**Konventionelle Immunsuppressiva**				
Glukokortikoide	Prednisolon	(I–) IIIa (dosisabhängig)	mild bis sehr hoch	> 20 mg/d: Pneumonien, Herpes Zoster (besonders in Kombination mit JAK-Inhibitoren); Pilzinfektionen, Sepsis
csDMARDs	Methotrexat (MTX)	II–IIIa (dosisabhängig)	moderat	Atemwegsinfektionen; Leukopenie
csDMARDs	Leflunomid	II	moderat	Hepatotoxizität; Leukopenie
csDMARDs	Sulfazalazin, Hydroxychloroquin	I	niedrig	vergleichbar mit Placebo
**Biologika (bDMARDs)**				
TNF-α-Inhibitoren	Adalimumab, Certolizumab, Etanercept, Golimumab, Infliximab	IIIa	hoch	Tuberkulose-Reaktivierung, Herpes Zoster, bakterielle Infektionen (Respirations- und Genitaltrakt)
IL-6-Inhibitoren	Tocilizumab, Sarilumab	IIIa	hoch	bakterielle Infektionen, atypische Verläufe ohne CRP/Fieber, Herpes Zoster
IL-17-Inhibitoren (IL-17A/F; IL-17R-Inhibitoren)	Ixekizumab, Secukinumab, Bimekizumab	IIIa	hoch	Mykosen (bei Bimekizumab hoch), milde Infektionen URT und Genitaltrakt, Herpes Zoster
IL-12/23-Inhibitoren	Ustekinumab	IIIa	moderat bis hoch	milde Infektionen URT und Genitaltrakt, Herpes Zoster
IL-23-Inhibitoren	Guselkumab, Risankizumab	IIIa	moderat bis hoch	Mykosen, Nasopharyngitis, Infektionen des URT
B-Zell-Depletion	Rituximab, Obinutuzumab	IIIb	hoch	Hypogammaglobulinämie, Hepatitis-B- und Herpes-Zoster-Reaktivierung
CTLA-4-Ig bzw. CD80/-86	Abatacept	IIIa	hoch	geringeres Risiko als bei TNF-α-Inhibition, Herpes Zoster
**Small Molecules (tsDMARDs)**				
JAK-Inhibitoren	Baricitinib, Filgotinib, Ruxolitinib, Tofacitinib, Upadacitinib	II–III	moderat	Herpes Zoster, Pneumonie, bakterielle Infektionen

*bDMARDs* biologische DMARDs, *csDMARDs* konventionelle, synthetische DMARDs, *CTLA‑4* cytotoxic T‑lymphocyte-associated Protein 4, *DMARDs* krankheitsmodifizierende Antirheumatika, *Ig* Immunglobulin, *IL* Interleukin, *ISP* Immunsuppression, *JAK* Januskinase, *TNF* Tumornekrosefaktor, *tsDMARDs* zielgerichtete, synthetische DMARDs

^a^Für die Beschreibung der Risikokategorien siehe Tab. 2 und 7

Abgesehen von einem erhöhten Infektionsrisiko (speziell Herpes Zoster) zeigte sich in der ORAL-Surveillance-Studie [76] bei Personen unter Tofacitinibtherapie im Vergleich zur Therapie mit Adalimumab ein erhöhtes Risiko, ein schweres unerwünschtes kardiovaskuläres Ereignis (MACE) oder ein Karzinom zu entwickeln. Die EMA empfiehlt seither einen vorsichtigen Einsatz bei Personen ≥ 65 Jahren oder einem anamnestischen Risiko für venöse Thromboembolie (VTE), MACE und Krebserkrankungen [77]. Diese Daten wurden zuletzt im deutschen Biologikaregister RABBIT bestätigt [78].

### 5.2 Dermatologie

In der Dermatologie kommen immunsuppressive oder immunmodulatorisch wirkende Substanzen für eine Vielzahl autoinflammatorischer Erkrankungen zum Einsatz. In der Behandlung von Psoriasis, Hidradenitis suppurativa oder Pyoderma gangraenosum finden vor allem TNF-α‑, IL-17-, IL-12/23- und IL-36-Inhibitoren Verwendung. Bei atopischer Dermatitis, Prurigo nodularis und bullösem Pemphigoid werden IL-4/13- oder IL-31-Inhibitoren eingesetzt. Zusätzlich sind – auch gegen Alopecia areata – JAK-Inhibitoren gebräuchlich. Autoimmunerkrankungen, wie bullöse Dermatosen oder Kollagenosen, werden – wie in der Rheumatologie – mit Anti-CD20-Antikörpern behandelt. Diese Antikörper werden auch bei kutanen B‑Zell-Lymphomen eingesetzt. *Mycosis fungoides* wird u. a. mit Anti-CD30-Antikörpern behandelt. Checkpoint-Inhibitoren, die immunmodulatorisch, aber nicht immunsuppressiv wirksam sind, werden vor allem beim Melanom oder bei fortgeschrittenen Plattenepithelkarzinomen eingesetzt.

Die meisten der angegebenen Substanzen führen zu einem erhöhten Risiko von Herpes Zoster (Tab. 9). Mit milden Infektionen der oberen Atemwege (URT, „upper respiratory tract“) sowie im Bereich des Genitaltrakts ist bei fast allen Biologikaanwendungen zu rechnen. Haut- und Weichteilinfektionen sind häufiger [79]. Bei TNF-α-Inhibitoren ist das Risiko für Reaktivierung einer Tuberkulose (Tbc) erhöht und daher muss vor Therapiebeginn eine latente Tbc ausgeschlossen werden [80]. Die Anwendung von IL-17-Inhibitoren erhöht das Risiko für Pilzinfektionen, v. a. Candida [79, 81]. Bei den JAK-Inhibitoren muss neben dem Risiko einer VZV-Reaktivierung eine Einschränkung in der Anwendungsempfehlung bei Personen ≥65 Jahren, erhöhtem kardiovaskulärem Risiko und stattgehabten thromboembolischen Ereignissen erwähnt werden [82].

**Tab. 9:** Infektionsrisiko durch/mit Therapien in der Dermatologie [75, 79–82]

**Substanzgruppe**	**Beispiele**	**ISP-Grad**	**Infektionsrisiko**	**Besondere Risiken/Hinweise**
**Biologika**				
TNF-α-Inhibitoren	Adalimumab, Infliximab	IIIa	hoch	Tuberkulose Reaktivierung, Herpes Zoster, bakterielle Infektionen (Respirations- und Urogenitaltrakt)
IL-17-Inhibitoren (IL-17A/F; IL-17R-Inhibitoren)	Ixekizumab, Secukinumab, Bimekizumab, Brodalumab	IIIa	hoch	Pilzinfektionen (bei Bimekizumab hoch), milde Infektionen URT und Urogenitaltrakt, Herpes Zoster
IL-12/23-Inhibitoren	Ustekinumab	IIIa	moderat bis hoch	milde Infektionen URT und Urogenitaltrakt, Herpes Zoster
IL-23-Inhibitoren	Guselkumab, Tildrakizumab, Risankizumab	IIIa	moderat bis hoch	Nasopharyngitis, Infektionen des URT
IL-36-Inhibitoren	Spesolimab	IIIa	moderat	Harnwegsinfektionen, Kontraindikation Tbc
IL-4/13-Inhibitoren	Dupilumab, Lebrikizumab, Tralokinumab (nur IL-13)	II	niedrig bis moderat	Herpes Zoster
IL-31-Inhibitoren	Nemolizumab	IIIa	moderat	oberflächliche Pilzinfektionen
B-Zell-Depletion	Rituximab	IIIb	hoch	Hypogammaglobulinämie, Hepatitis-B- und Herpes-Zoster-Reaktivierung
Checkpoint-Inhibitoren	Pembrolizumab, Nivolumab, Ipilimumab, Cemiplimab, Avelumab, Durvalumab	0	niedrig	Bakterielle oder virale Infektionen (Pneumonie, Haut-, Weichgewebs-, Urogenitaltraktinfektionen), Pilzinfektionen
**Small Molecules**				
JAK-Inhibitoren	Abrocitinib, Baricitinib, Upadacitinib, Ritlecitinib, Deucravacitinib	II–III	moderat	Herpes Zoster, Pneumonie, bakterielle Infektionen
Phosphodiesterase-4-Inhibitoren	Apremilast	I	niedrig	–

*IL* Interleukin, *ISP* Immunsuppression; *JAK* Januskinase, *KHK* koronare Herzkrankheit, *St.p.* Status post, *Tbc* Tuberkulose, *TNF* Tumornekrosefaktor, *URT* „upper respiratory tract“ = oberer Respirationstrakt

^a^Für Beschreibung der Risikokategorien siehe Tab. 2 und 7

### 5.3 HIV

Das Risiko für Infektionserkrankungen hängt vom Grad der Immunsuppression und der Effektivität der Virussuppression ab (Tab. 10).

**Tab. 10:** HIV – Einteilung nach Grad der Immunsuppression [83, 84]

**ISP-Grad**	**Kinder < 12 Monate, CD4**^**+**^**-Zahl**	**Kinder 1–5 Jahre, CD4**^**+**^**-Zahl**	**Erwachsene und Kinder ≥ 6 Jahre, CD4**^**+**^**-Zahl**
*Grad 0–I*	≥ 1500 Zellen/µl	≥ 1000 Zellen/µl	≥ 500 Zellen/µl
*Grad II*	750–1499 Zellen/µl	500–999 Zellen/µl	200–499 Zellen/µl
*Grad IIIa*	< 750 Zellen/µl	< 500 Zellen/µl	< 200 Zellen/µl

*HIV*  humanes Immundefizienzvirus, *ISP* Immunsuppression

Ein erhöhtes Risiko besteht für Koinfektionen mit Hepatitis B, Hepatitis C, humanem Papillomavirus (HPV) und Syphilis. In Bezug auf impfpräventable Erkrankungen ist die Inzidenz für durch Influenza verursachte Pneumonien und invasiver Pneumokokkenerkrankungen erhöht. Hepatitis-A-Infektionen können je nach Risikoverhalten häufiger auftreten (bei Reisetätigkeit, Männern, die Sex mit Männern haben [MSM], Sexarbeit, intravenösem Drogenkonsum [IVDU], aktiver Hepatitis-B- und -C-Infektion). Das Risiko für Meningokokkeninfektionen ist bei entsprechendem Risikoprofil erhöht (Reise, enger Kontakt zu Kindern, MSM). Primärinfektionen durch Varizellen können schwerer verlaufen und die Reaktivierung/Herpes Zoster tritt häufiger auf bzw. mit schwererem Verlauf. Das Risiko für schwerere RSV-Infektionen ist vor allem bei älteren HIV-Infizierten erhöht. Während das Vorliegen einer kontrollierten HIV-Infektion den Verlauf einer Mpox-Infektion nicht zu beeinflussen scheint, sind bei fortgeschrittener Immunsuppression schwere Verläufe beschrieben worden [85].

Zusätzlich besteht allgemein ein erhöhtes Risiko für opportunistische Infektionen mit *Mycobacterium tuberculosis* (unabhängig von der CD4-Zellzahl), *Mycobacterium avium complex, Pneumocystis jirovecii* (PCP), mukokutane Candidainfektionen (CD4^+^ < 200 Zellen/µl), HSV, Cryptokokken, Cryptosporidiose, John-Cunningham(JC)-Virus, Cytomegalovirus (CMV) oder Toxoplasmose (Enzephalitis; [86, 87]).

### 5.4 Gastroenterologie

Zu den chronisch-entzündlichen Darmerkrankungen (CED) werden Morbus Crohn und Colitis ulcerosa gezählt, wobei bei Morbus Crohn der gesamte Gastrointestinaltrakt und alle Wandschichten betroffen sein können und bei Colitis ulcerosa das Kolon mit mukosalen Entzündungen, Pseudopolypen und Schleimhautblutungen hauptbetroffen ist.

Die eingesetzten immunsuppressiv wirksamen Medikationen (besonders in Kombinationen) können zu einem deutlich erhöhten Risiko für bestimmte Infektionen führen, wobei dieses Risiko auch durch weitere Faktoren wie Malnutrition oder Adipositas, Komorbiditäten, akute Krankheitsschübe oder bei höherem Alter verstärkt sein kann.

Folgende Medikationen sind mit einer erhöhten Infektionsanfälligkeit assoziiert: In Abhängigkeit von der Dosierung ist die Therapie mit Glukokortikoiden, aber auch mit TNF-α-Inhibitoren (besonders in Kombination), mit einem verstärkten Auftreten von Herpes-Zoster-Reaktivierungen assoziiert. Es besteht ein erhöhtes Risiko für Tbc-Reaktivierung wie auch für bakterielle Infektionen (Tab. 11). Beim Einsatz von JAK-Inhibitoren muss man besonders bei der Therapie mit Upadacitinib vermehrt mit Herpes-Zoster-Episoden rechnen. Außerdem kann es gehäuft zu bakteriellen Infektionen, inkl. Pneumonien, kommen. Im Unterschied dazu wirken die Integrin-α4β7-Blocker, die die Migration von T‑Zellen in den Darm inhibieren, nicht (systemisch) immunsuppressiv und sind daher mit keiner erhöhten Infektionsanfälligkeit verbunden. Therapien mit Sphingosinrezeptorinhibitoren, wie Ozanimod, können zu Lymphopenie führen, sind jedoch nur in moderatem Ausmaß mit vermehrten Infektionen assoziiert.

**Tab. 11:** Infektionsrisiko durch/mit Therapien in der Gastroenterologie [71, 75, 88–92]

**Substanzgruppe**	**Beispiele**	**ISP-Grad**	**Infektionsrisiko**	**Besondere Risiken/Hinweise**
**Konventionelle Immunsuppressiva**				
Aminosalicylate	Sulfasalazin (SASP), 5-Aminosalizylsäure (5-ASA)	I	niedrig	–
csDMARDs	Methotrexat (MTX)	II	moderat	>0,4 mg/kg/Wo: Atemwegsinfektionen; Leukopenie
csDMARDs	Azathioprin (AZA)	II	moderat	> 3 mg/kg/d: opportunistische Infektionen (bei Kombination mit MTX, Kortison)
csDMARDs	Ciclosporin, Tacrolimus	IIIa	hoch	Opportunistische Infektionen bes. bei Kombinationstherapien
Glukokortikoide	Prednisolon, Budesonid	I–IIIa (dosisabhängig)	niedrig bis sehr hoch	> 20 mg/d: Pneumonien, Herpes Zoster (besonders in Kombination mit JAK-Inhibitoren), Pilzinfektionen; Sepsis
**Biologika**				
TNF-α-Inhibitoren	Adalimumab, Infliximab, Golimumab	IIIa	moderat bis hoch	Tuberkulosereaktivierung, Herpes Zoster, bakterielle Infektionen
IL-12/23-Inhibitoren	Ustekinumab (IL-12/23), Risankizumab, Mirikizumab, Guselkumab	II–IIIa	niedrig	Infektionen des URT
Integrin-α4β7-Blocker	Vedolizumab	II	niedrig	Keine, da keine systemische Immunsuppression
**Small Molecules**				
S1PR-Modulatoren	Ozanimod, Etrasimod	II	moderat	Lymphopenie; kaum Infektions-assoziiert
JAK-Inhibitoren	Tofacitinib, Filgotinib, Upadacitinib	II–III	moderat	Herpes Zoster (besonders bei Upadacitinib!), Pneumonie, bakterielle Infektionen

*d* Tag, *IL* Interleukin, *JAK* Januskinase, *MTX* Methotrexat, *S1PR* Sphingosin-1-Phosphat-Rezeptor, *TNF* Tumornekrosefaktor, *URT* „upper respiratory tract“ = oberer Respirationstrakt, *Wo* Woche

^a^Für Beschreibung der Risikokategorien siehe Tab. 2 und 7

### 5.5 Neurologie

Die neuroimmunologischen Erkrankungen werden in immun-mediierte Erkrankungen des Zentralnervensystems (ZNS), des peripheren Nervensystems (PNS) und der Muskulatur eingeteilt (Tab. 12).

**Tab. 12:** Einteilung immunmediierter Erkrankungen und Therapiebeispiele in der Neurologie [93, 94]

**Erkrankungen**	**Therapien (beispielgebend)**
**Immunmediierte Erkrankungen des ZNS**	
Multiple Sklerose	IFN‑β, Glatirameracetat, Dimethylfumarat, Teriflunomid
	Cladribin, S1PR-Modulatoren: Fingolimod, Siponimod, Ozanimod, Ponesimod
	Natalizumab, Alemtuzumab
	Anti-CD20-Antikörper: (off-label: RTX), Ocrelizumab, Ofatumumab, Ublituximab
„Neuromyelitis optica spectrum disorder“ (NMOSD)	AZA, MMF, RTX, Tocilizumab (alle off-label)
	Eculizumab, Satralizumab, Inebilizumab, Ravulizumab
Myelin-Oligodendrozyten-Glykoprotein-Antikörper-assoziierte Erkrankung (MOGAD)	AZA, IVIg, MMF, RTX (alle off-label)
Akute disseminierte Enzephalomyelitis (ADEM)	IVMP; PLEX
Autoimmunenzephalitis	AZA, MMF, RTX, Cyclophosphamid
T‑Zell-mediierte ZNS-Erkrankungen (Rasmussen-Enzephalitis)	Tacrolimus, Cyclophosphamid
Neurosarkoidose	MTX, RTX, Cyclophosphamid, Infliximab, Steroide
Zerebrale Vaskulitis	MTX, RTX, Cyclophosphamid, Infliximab, Steroide
**Immunmediierte Erkrankungen des PNS**	
Guillain-Barré-Syndrom	Akute Therapie: PLEX; IVIg
„Chronic inflammatory demyelinating polyneuropathy“ (CIDP)	PLEX, IVIg, AZA, MMF, MTX; Cyclophosphamid
**Immunmediierte Erkrankungen der Muskulatur**	
Myasthenia gravis und myastene Syndrome	Akute Therapie: PLEX; IVIg; Thymektomie, AZA, Kortison, MMF, Tacrolimus, MTX, Eculizumab, Ravulizumab, Zilucoplan (Komplementinhibitoren), FcRn-Modulatoren

*AZA* Azathioprin, *FcRn* neonataler Fc-Rezeptor, *IFN* Interferon, *IVIg* intravenös verabreichtes Immunglobulin, *IVMP* intravenös verabreichtes Methylprednisolon, *MMF* Mycophenolat mofetil, *MTX* Methotrexat, *PLEX* Plasmapherese, *PNS* peripheres Nervensystem, *RTX* Rituximab, *S1PR* Sphingosin-1-Phosphat-Rezeptor, *ZNS* Zentralnervensystem

Eine immunmediierte neuroimmunologische Erkrankung, wie Multiple Sklerose, ist ohne Therapie nicht mit einer Immunsuppression assoziiert und daher nicht mit einem erhöhten Infektionsrisiko verbunden. Allerdings können Faktoren wie Alter, Geschlecht, Ausmaß der neurologischen Beeinträchtigung, bestehende Komorbiditäten oder verschiedene Therapieformen für unterschiedliche Infektionen prädisponieren. Prinzipiell kommen Infektionen des oberen und unteren Respirationstrakts (Pneumonien und Influenzainfektionen) und Harnwegsinfektionen häufiger vor, je länger die Erkrankung vorliegt. Das Risiko für schwere Infektionen wird aber vorwiegend durch die Art der Therapien beeinflusst. Das Risiko für infektionsbedingte Hospitalisierung ist am höchsten unter Alemtuzumab, Anti-CD20-Antikörpern (geringeres Infektionsrisiko unter subkutan verabreichtem Ofatumumab), gefolgt von Natalizumab und Fingolimod. Das geringste Infektionsrisiko besteht unter Behandlung mit Interferon‑β und Glatirameracetat. Grundsätzlich besteht unter allen bei der Multiplen Sklerose eingesetzten Medikamenten (Ausnahme: Glatirameracetat, β-Interferone) ein erhöhtes Herpes-Zoster-Risiko, das sich nicht allein durch den Grad der Immunsuppression oder der Lymphopenie bestimmen lässt ([95]; Tab. 13).

**Tab. 13:** Infektionsrisiko durch/mit Therapien in der Neurologie [75, 96]

**Substanzgruppe**	**Beispiele**	**ISP-Grad**	**Infektionsrisiko**	**Besondere Risiken/Hinweise**
**Konventionelle Immunsuppressiva**				
–	Glatirameracetat	I	gering	Kein Intervall zu Lebendimpfungen
–	Dimethylfumarat (DMF)	II	moderat	Lymphozytopenie; Atemwegsinfektionen, Herpes Zoster, PML
csDMARDs	Methotrexat (MTX)	II–IIIa (dosisabhängig)	moderat	>25 mg/Wo: Atemwegsinfektionen, Leukopenie
csDMARDs	Azathioprin (AZA)	II–III	moderat	>150 mg/d: opportunistische Infektionen
csDMARDs	Mycophenolat mofetil (MMF)	IIIa	hoch	Bronchitis, Harnwegsinfektionen, Herpes Zoster
csDMARDs	Ciclosporin	IIIa	hoch bis sehr hoch	Herpes simplex
csDMARDs	Tacrolimus	IIIa	hoch	–
csDMARDs	Cyclophosphamid	IIIa	hoch	Pneumonien, Hepatitis B/C, Tuberkulose
Glukokortikoide	Prednisolon	I–IIIa (dosisabhängig)	niedrig–sehr hoch	> 20 mg/d: Pneumonien, Herpes Zoster (besonders in Kombination mit JAK-Inhibitoren), Pilzinfektionen, Sepsis
**Biologika**				
IFN-β/GA	β-Interferone	I–II	gering	Respiratorische Infektionen
TNF-α-Inhibitoren	Infliximab	IIIa	hoch	Tuberkulose-Reaktivierung, Herpes Zoster, bakterielle Infektionen
IL-6-Rezeptor-Inhibitoren	Tocilizumab, Satralizumab	IIIa	hoch	Bakterielle Infektionen, atypische Verläufe ohne Fieber, Herpes Zoster
B-Zell-Depletoren	Rituximab, Ocrelizumab, Inebilizumab, Ofatumumab, Ublituximab	IIIb	hoch	Herpesvirusinfektionen, HBV-Reaktivierung, Atemwegsinfektionen, Harnwegsinfektionen (Cave: Hypogammaglobulinämie)
Anti-CD52-Antikörper	Alemtuzumab	III	sehr hoch (bes. in ersten Monaten)	Herpes-Reaktivierung, bes. Herpes Zoster, bakterielle Infektionen (Atemwege, Harnwegsinfektionen), Pneumonien, opportunistische Infektionen
α4-Integrin-Inhibitor	Natalizumab	III	moderat	Cave: PML durch opportunistische ZNS-Infektionen
Komplementinhibitor en	Eculizumab, Ravulizumab, Zilucoplan	III	hoch	Meningokokken, Pneumokokken, HiB-Infektion
FcRn-Blocker	Efgartigimod-alfa, Rozanolixizumab	II–(III)	leicht bis moderat	Infektionen des URT
**Small Molecules**				
Purinanaloga	Cladribin	IIIa	moderat	Herpesinfektionen inkl. Herpes zoster
S1PR-Modulatoren	Fingolimod, Siponimod, Ozanimod, Ponesimod	III	hoch	Herpes Zoster; Herpes simplex; Bronchitis, Pneumonie, erhöhtes Risiko für opportunistische Infektionen (PML, Kryptokokkenmeningitis)

*FcRn* neonataler Fc-Rezeptor, *GA* Glatirameracetat, *HBV* Hepatitis-B-Virus, *HiB Haemophilus influenzae, HPV* humanes Papillomavirus, *IFN* Interferon, *IL* Interleukin, *ISP* Immunsuppression, *JAK* Januskinase, *PML* progressive multifokale Leukenzephalitis, *S1PR* Sphingosin-1-Phosphat-Rezeptor, *TNF* Tumornekrosefaktor, *URT* „upper respiratory tract“ = oberer Respirationstrakt, *ZNS* Zentralnervensystem

^a^Für Beschreibung der Risikokategorien siehe Tab. 2 und 7

### 5.6 Onkologie und Hämatoonkologie

#### 5.6.1 Solide Tumoren

Infektionen stellen für Personen mit Krebserkrankung eine schwerwiegende Komplikation dar. Daher ist die Impfprophylaxe bei Krebspatienten ein essenzieller Teil des Behandlungskonzepts. Fehlende Impfungen sollten möglichst frühzeitig bei Diagnosestellung und vor Therapie komplettiert werden [97]. Klassische/konventionelle Chemotherapien führen zu einer temporären Immunsuppression [98], weshalb – je nach Dringlichkeit – Impfungen während der Therapie meist vermieden werden.

Nach Therapieende können inaktivierte Impfungen nach 3 Monaten verabreicht und mit gutem Impferfolg gerechnet werden, da zu diesem Zeitpunkt i. d. R. die Immunkompetenz wieder hergestellt ist. Gegen saisonale respiratorische Infektionen wie COVID-19, RSV und Influenza soll aber auch während der Therapie und unabhängig vom Krankheitsstatus geimpft werden, da das Erkrankungsrisiko besonders hoch ist. Lebendimpfstoffe sollten frühestens 6 Monate nach Abschluss der Antitumortherapie verabreicht werden; sie können nicht während der Immuntherapie gegeben werden.

Auch zielgerichtete Therapie wie einige Tyrosinkinaseinhibitoren oder Antikörper-Wirkstoff-Konjugate (ADC) können die Immunantwort temporär verringern, ihre Effekte sind allerdings je nach Wirkstoff sehr unterschiedlich [98–101]. Sollte während der Therapie mit konventioneller Chemotherapie oder zielgerichteter Therapie unter Einbeziehung des individuellen Risikos eine Impfung notwendig sein, so sollte diese zwischen den Zyklen durchgeführt werden, wenn die Anzahl der Leukozyten bzw. der Neutrophilen im Normbereich liegt.

Checkpoint-Inhibitoren führen zu keiner Immunsuppression [99, 102]. Impfungen gegen COVID-19 und Influenza scheinen grundsätzlich sicher zu sein [13], es gibt aber Berichte, dass es zu einer im Vergleich zu konventionellen Chemotherapien erhöhten zellmediierten Immunantwort kommen [103] und die Rate an immunvermittelten Nebenwirkungen der Checkpoint-Inhibitor-Therapie erhöht sein kann [12]. Umgekehrt gibt es aber auch Hinweise, dass die mRNA-Impfung das Ansprechen auf Checkpoint-Inhibitoren verbessern kann [14]. Prinzipiell gibt es mit Ausnahme von Influenza und COVID-19 derzeit nur wenige belastbare Daten zur Impfung während der Gabe von Checkpoint-Inhibitoren. Deshalb sollte grundsätzlich individuell vorgegangen werden, insbesondere bei Lebendimpfungen (Tab. 14).

**Tab. 14:** Infektionsrisiko durch/mit (neo)adjuvante/n Therapien und palliative/n Erstlinientherapien bei soliden Tumoren: beispielgebend bei Lungen‑, Brust- und Kolonkarzinom

**Substanzgruppe**	**Beispiele**	**ISP- Grad**	**Infektionsrisiko**	**Besondere Risiken/Hinweise**
**Konventionelle Chemotherapie**				
–	Cisplatin, Carboplatin, Paclitaxel, Docetaxel, Etoposid, Pemetrexed, Doxorubicin, Epirubicin, Cyclophosphamid, Oxaliplatin, Irinotecan	III	hoch	Lebendimpfstoffe kontraindiziert während Therapie, reduzierte Impfantwort, Herpes Zoster, Pneumonien (Pneumokokken und Influenza), bakterielle Infektionen
–	Capecitabin, Pemetrexed, 5-Fluorouracil	II	moderat	Lebendimpfstoffe kontraindiziert während Therapie, reduzierte Impfantwort, Herpes Zoster, Pneumonien (Pneumokokken und Influenza), bakterielle Infektionen
**Biologika**				
EGFR-Antikörper	Cetuximab, Panitumumab	0–I	keine bis gering	Impfantwort meist erhalten, keine spezifischen Impfkontraindikationen
Anti-HER2 Antikörper	Trastuzumab, Pertuzumab	0–I	keine bis gering	Impfantwort meist erhalten, keine spezifischen Impfkontraindikationen
HER2-ADC	Trastuzumab-Deruxtecan, T-DM1	II	moderat	Lebendimpfstoffe kontraindiziert während Therapie, reduzierte Impfantwort möglich
Anti-VEGF Angiogenesehemme r	Bevacizumab, Ramucirumab	0–I	keine bis gering	Impfantwort meist erhalten, keine spezifischen Impfkontraindikationen
Checkpoint- Inhibitoren	Pembrolizumab, Nivolumab, Cemiplimab, Avelumab, Durvalumab, Atezolizumab	0	keines bis gering	Nicht mit erhöhter Infektionsanfälligkeit assoziiert
Checkpoint- Inhibitoren	Ipilimumab, Tremelilumab	0	keines bis gering	Nicht mit erhöhter Infektionsanfälligkeit assoziiert
**Small Molecules**				
EGFR-TKI	Osimertinib, Erlotinib, Gefitinib	0–I	keine bis gering	Impfantwort meist erhalten, keine spezifischen Impfkontraindikationen
ALK-TKI	Alectinib, Brigatinib, Crizotinib	0–I	keine bis gering	Impfantwort meist erhalten, keine spezifischen Impfkontraindikationen
CDK4/6-Inhibitoren	Palbociclib, Ribociclib, Abemaciclib	II	moderat	Lebendimpfstoffe kontraindiziert während Therapie, reduzierte Impfantwort möglich
Endokrine Therapie	Tamoxifen, Letrozol, Anastrozol, Exemestan	0–I	keine bis gering	Impfantwort meist erhalten, keine spezifischen Impfkontraindikationen
PARP-Inhibitoren	Olaparib, Talazoparib	II	moderat	Lebendimpfstoffe kontraindiziert während Therapie, reduzierte Impfantwort möglich
Multikinase-Inhibitoren	Regorafenib, Fruquintinib	II	moderat	Lebendimpfstoffe kontraindiziert während Therapie, reduzierte Impfantwort möglich
BRAF/MEK-TKI	Dabrafenib/Trametinib, Vemurafenib/Cobimetinib, Encorafenib/Binimetinib	0–I	keine bis gering	Impfantwort meist erhalten, keine spezifischen Impfkontraindikationen

Quellen: siehe die jeweiligen Fachinformationen sowie [98, 99, 104–106]

*ADC* Antikörper-Wirkstoff-Konjugate, *ALK* „anaplastic lymphoma kinase“, *BRAF* „B-Raf proto-oncogene“, *CDK4/6* cyclinabhängige Kinasen 4 und 6, *CTLA‑4* „cytotoxic T‑lymphocyte-associated Protein 4“, *EGFR* epidermaler Wachstumsfaktor, *HER2* „human epidermal growth factor receptor 2“, *ISP* Immunsuppression, *MEK* „mitogen-activated protein kinase“, *PARP* Poly(ADP-Ribose)-Polymerase, *PD‑1* „programmed cell death protein 1“, *PD-L1* „programmed cell death-ligand 1“, *T‑DM1* Trastuzumab-Emtansin, *TKI* Tyrosinkinaseinhibitor, *VEGF* „vascular endothelial growth factor“

^a^Für Beschreibung der Risikokategorien siehe Tab. 2 und 7

#### 5.6.2 Hämatologische Neoplasien

Die in der Hämatologie therapeutisch eingesetzten Substanzen, die hinsichtlich Impfungen besonderes relevant sind, umfassen: Antikörper, Tyrosinkinaseinhibitoren, „B-cell-lymphoma-2“(BCL2)-Inhibitoren, Checkpoint-Inhibitoren, immunmodulierende Substanzen und klassische, konventionelle Chemotherapien [107, 108].

Alle Anti-CD20-Antikörper (Rituximab, Ofatumumab, Obinutuzumab), die vor allem bei lymphoproliferativen Erkrankungen eingesetzt werden, führen zu einer langanhaltenden B‑Zell-Depletion [109]. Damit ist vor allem die humorale Impfantwort deutlich reduziert [109]. Impfungen sollten daher frühestens 6 Monate nach der letzten Dosis des Anti-CD20-Antikörpers erfolgen und optimalerweise auch dann nur nach Erreichen einer adäquaten Immunrekonstitution mit B‑Zellen ≥ 20/µl und Immunglobulin G > 400 mg/dl [107, 108].

Bruton-Tyrosinkinase-Inhibitoren (BTKi, z. B. Ibrutinib, Acalabrutinib, Zanubrutinib) führen ebenfalls zu einer B‑Zell-Depletion und damit zu einer reduzierten Impfantwort, allerdings kann diese durch Pausieren der Therapie verbessert werden, da sich die B‑Zell-Funktion nach Absetzen innerhalb weniger Tage wiederherstellt [110].

Unter JAK2-Inhibitoren (z. B. Ruxolitinib), die zur Behandlung myeloproliferativer Erkrankungen eingesetzt werden, kommt es zu einer Blockade der intrazellulären Signalweiterleitung von Zytokinen [98]. Dadurch kann eine Immunantwort reduziert sein (siehe Abschnitt 6.3.3), inaktivierte Impfstoffe sind aber auch während der Behandlung erlaubt und sicher [98, 107, 108]. Das Risiko für Herpes-Zoster-Reaktivierungen ist erhöht.

Unter BCL2-Inhibitoren (z. B. Venetoclax) sind inaktivierte Impfstoffe ebenfalls erlaubt, wobei bei Personen mit chronisch-lymphatischer Leukämie (CLL) durch die Erkrankung an sich ein erhöhtes Infektionsrisiko besteht, Impfungen also besonders wichtig sind [111].

Bei der Behandlung des Multiplen Myeloms werden sowohl in der Induktion als auch in der Erhaltung in vielen Schemata Immunmodulatoren (IMiDs, z. B. Lenalidomid, Pomalidomid) eingesetzt. Diese sind nur mäßig immunsuppressiv. Inaktivierte Impfstoffe können daher sicher verabreicht werden [112], Lebendimpfstoffe allerdings nur bei dringlicher Indikation [107, 108, 112]. Aufgrund einer möglichen Neutropenie kann es generell gehäuft zu Infektionen kommen [112].

Die Anti-CD38-Antikörper (Daratumumab, Isatuximab) sind spezifisch mit einem erhöhten Risiko für Herpes-Zoster-Reaktivierungen verbunden [112, 113]. Die Kombination mit Lenalidomid und Dexamethason führt zu einer moderaten Immunsuppression, insbesondere durch die Reduktion der NK-Zellen [113]. Impfantworten gegen inaktivierte Impfstoffe sind i. d. R. deutlich reduziert und das Antikörper-Waning erhöht. Erste Daten zeigen, dass der lebend-attenuierte Masern-Mumps-Röteln(MMR)-Impfstoff unter Anti-CD38-Antikörpertherapie bei Patienten mit Multiplem Myelom (MM) nach autologer SZT sicher zu sein scheint [114].

Beim Myelodysplastischen Syndrom wird routinemäßig die hypomethylierende Substanz Azacitidin verwendet, die zu Zytopenien führen kann, aber im Gegensatz zur konventionellen Chemotherapie nicht mit einer hochgradigen Immunsuppression assoziiert ist [115]. Klassisch zytostatisch sind immer noch unterschiedliche Substanzklassen in der Hämatologie im Einsatz. Alle führen zu einer temporären Immunsuppression, weshalb – je nach Dringlichkeit – Impfungen während der Therapie meist vermieden werden und erst 3 bis 6 Monate nach Therapieende empfohlen sind ([97, 107, 108]; Tab. 15).

**Tab. 15:** Infektionsrisiko durch/mit Therapien bei hämatologischen Systemerkrankungen

**Substanzgruppe**	**Beispiele**	**ISP-Grad**	**Infektionsrisiko**	**Besondere Risiken/Hinweise**
**Konventionelle Chemotherapien**				
Zytostatika	Doxorubicin, Cyclophosphamid, Etoposid	III	hoch	Bakterielle und virale Infektionen, Herpes Zoster, opportunistische Infektionen
Hypomethylierende Substanzen	Azacitidin	II	moderat	Bakterielle Infektionen (Pneumonien, Sepsis), opportunistische Infektionen
**Biologika**				
Anti-CD20-Antikörper	Rituximab	IIIb	hoch bis sehr hoch	Virale Infektionen, Herpes Zoster, bakterielle Infektionen (Cave: Hypogammaglobulinämie)
Anti-CD38-Antikörper	Daratumumab	III	moderat bis hoch (bes. bei Kombinationstherapien)	spezifisch erhöhtes Risiko für Herpes Zoster Reaktivierungen, HBV-Reaktivierung; gehäufte respiratorische Infektionen, bakterielle Pneumonien wegen Reduktion der NK- und B-Zellen (Hypogammaglobulinämie)
BiTE	Blinatumomab	IIIa	hoch	Bakterielle Infektionen, virale Reaktivierungen
ADC	Inotuzumab, Gemtuzumab	0–III	gering bis hoch – abhängig vom Antikörper und vom Wirkstoff	Gehäufte bakterielle, virale und opportunistische Infektionen
**Small Molecules**				
IMiDs	Lenalidomid, Thalidomid, Pomalidomid	II	moderat	Neutropenie → erhöhte bakterielle und virale Infektionen
BTKi	Zanubrutinib, Ibrutinib, Acalabrutinib	II–III	moderat	Bakterielle und virale Infektionen, Sepsis (selten)
TKI (BCR-ABL)	Dasatinib, Imatinib, Nilotinib	II	moderat	Bakterielle und virale Infektionen
JAK-Inhibitoren	Ruxolitinib	II–III	moderat	Herpes Zoster
BCL2-Inhibitoren	Venetoclax	II–III	moderat	Bakterielle und virale Infektionen

Quellen: siehe die jeweiligen Fachinformationen sowie [98, 99, 104–106]

*ADC* Antikörper-Wirkstoff-Konjugat, *BCL2* B cell lymphoma 2, *BCR-ABL* breakpoint cluster region-Abelson murine leukemia viral oncogene, *BiTE* bispezifischer T-Cell-Enganger, *BTKi* Bruton-Tyrosinkinase-Inhibitor, *HBV* Hepatitis-B-Virus, *IMiDs* immunmodulatorische Wirkstoffe, *ISP* Immunsuppression, *JAK* Januskinase, *NK-Zellen* natürliche Killerzellen, *TKI* Tyrosinkinaseinhibitor

^a^Für Beschreibung der Risikokategorien siehe Tab. 2 und 7

##### 5.6.2.1 Allogene hämatopoetische Stammzelltransplantation

Bei der konditionierenden Chemotherapie werden mehrere Substanzen kombiniert, die klassisch zytotoxisch, aber auch lymphodepletierend sind (z. B. Fludarabin, Cyclophosphamid, im Falle einer Stammzelltransplantation bei angeborenem Immundefekt kommt auch das Reduced-intensity-conditioning[RIC]-Schema zur Anwendung). Zur Graft-versus-Host-Prophylaxe werden zusätzlich Calcineurininhibitoren und oftmals Antithymozytenglobulin (ATG) eingesetzt. Insgesamt kommt es daher zu einer langanhaltenden Immunsuppression. Insbesondere die CD4-positiven T‑Zellen brauchen mindestens 1 Jahr bis zur Regeneration, aber auch B‑Zellen sind oftmals erst nach 100 Tagen nachweisbar. Kommt es zu einer aktiven Graft-versus-Host-Erkrankung, muss die Immunsuppression oft über einen noch längeren Zeitraum angenommen werden (siehe Tab. 21). Lebendimpfstoffe werden üblicherweise nach 2 Jahren verabreicht. Bei erhöhtem epidemiologischen Risiko (wie z. B. Masernausbruch) kann bei Personen mit Immunrekonstitution eine Lebendimpfung mit MMR ab 12 Monaten nach individueller Nutzen-Risiko-Analyse verabreicht werden [116].

##### 5.6.2.2 Autologe hämatopoetische Stammzelltransplantation

Der Grad der Immunsuppression ist bei der allogenen insgesamt deutlich höher als bei der autologen Stammzelltransplantation, bei der die Immunsuppression wesentlich durch das myeloablative Konditionierungsschema (z. B. Melphalan, BEAM-Schema) bestimmt wird. Nach 3 Monaten ist aber bereits eine immunologische Rekonstitution im Gange, weshalb bereits da mit einer Revakzinierung gestartet werden kann. Lebendimpfstoffe sollten frühestens 12 Monate nach Stammzelltransplantation verabreicht werden (siehe Tab. 21).

##### 5.6.2.3 CAR-T-Zell- und andere Zell-basierte Immuntherapien

Die derzeit bekannten Chimeric-antigen-receptor(CAR)-basierten Immuntherapien nutzen T‑Zellen, es sind aber auch alternative Immunzellplattformen (z. B. NK-, NKT-, γδ-T-Zellen) in Entwicklung [117]. Diese könnten z. B. bei der akuten myeloischen Leukämie (AML) zum Einsatz kommen, wo viele für die CAR-T-Zell-Therapie zugängliche Oberflächenantigene auch auf gesunden hämatopoetischen Stamm- und Vorläuferzellen exprimiert werden und es daher zu bedeutenden Off-Target/Off-Tumor-Toxizitäten und einer langanhaltenden Myeloablation kommt [117]. Zunehmend kommen CAR-basierte Immuntherapien auch in anderen Disziplinen zum Einsatz, z. B. bei SLE.

Zu den bekannten Nebenwirkungen von CAR-T-Zell-Behandlungen gehören Zytopenien, Hypogammaglobulinämie, Mangel an T‑Helferzellen, persistierende B‑Zell-Aplasie sowie spät auftretende Neoplasien. Die bisher beobachtete Non-Relapse-Mortalität ist bis zu 70 % auf Infektionen zurückzuführen [118].

Die wesentlichen Schlüsselparameter der Immunrekonstitution sind auch hier CD4-Helferzellen > 200/µl, Immunglobulin G > 400 mg/dl sowie B‑Zellen ≥ 20/µl.

Es wurde in einer Studie gezeigt, dasss prä-CAR-T-vakzinierte Personen den Impfschutz bezüglich Masern, Mumps, Röteln, VZV, Diphtherie und Tetanus nach Therapie nicht verlieren, wobei die Antikörperspiegel nach CD19-CAR-T-Zell-Therapie besser erhalten bleiben, als nach BCMA-CAR-T-Zell-Therapie [119, 120]. Diese Tatsache sollte in der Vorbereitungsphase einer CAR-T-Zell-Therapie bedacht werden.

Aufgrund reduzierten Impfansprechens sollte jedenfalls gegen epidemisch auftretende Erreger, wie Influenza, RSV oder SARS-CoV‑2, vor dem Start der notwendigen lymphodepletierenden Konditionierung (spätestens 2 Woche davor) geimpft werden. Als weitere Impfungen vor CAR-T-Zell-Therapie können Pneumokokken, Herpes Zoster und Hepatitis B in Betracht gezogen werden [121].

Generell sollten Impfungen nach CAR-T-Zell-Therapie nicht in fix vorgegebenen Zeitintervallen erfolgen, sondern man sollte sich an der Immunrekonstitution orientieren. Ein immunologisches Assessment bzw. eine Impferfolgskontrolle sollten in der Nachsorge nach > 6 Monaten erhoben werden. Die Immunisierung speziell gegen saisonale Infektionen wie **COVID-19, Influenza und RSV sowie gegen Pneumokokken** sollte als erste angeboten werden, da es nach CAR-T-Zell-Therapie häufig zu respiratorischen Infektionen kommt. Auch sind **Herpes-Zoster-Impfungen** bei diesen Personen besonders indiziert.

Bei inaktivierten Vakzinen empfehlen internationale Guidelines einen Abstand von ≥ 3 Monaten für saisonale Impfungen (Influenza und COVID-19) sowie von ≥ 6 Monaten für die weiteren inaktivierten Impfstoffe, sofern die Immunrekonstitution nachgewiesen ist und keine immunsuppressiven Therapien laufen [99, 122]. Für Lebendimpfungen gilt ein Richtwert von ≥ 12 Monaten bei kompletter Immunrekonstitution nach CAR-T-Zell-Therapie, keiner laufenden immunsuppressiven Behandlung und kein IVIg für mindestens 8 Monate [99, 122].

Es ist anzumerken, dass die derzeitige Empfehlung von Impfungen vor und nach CAR-T-Zell-Therapie auf extrapolierten Daten nach allogener hämatopoetischer Stammzelltransplantation beruht und bei Vorhandensein einer besseren Datenlage ggf. eine Optimierung des Impfschemas erfolgen wird [123].

### 5.7 Solide Organtransplantation

Personen nach solider Organtransplantation (SOT) sind besonders vulnerabel und haben ein deutlich erhöhtes Infektionsrisiko in Abhängigkeit von der zugrunde liegenden Erkrankung (Indikation der SOT) und der immunsuppressiven Therapie. Die Mehrheit der zum Einsatz kommenden Therapien zur Vorbeugung von Transplantatabstoßungen reduzieren sowohl die humorale wie auch die zelluläre Abwehr, wodurch die Impfantworten stark reduziert werden; als Beispiel sei hier Mycophenolat mofetil genannt. Daher ist eine frühzeitige Planung der Infektionsprävention bei SOT-Kandidaten entscheidend, ebenso wie eine kontinuierliche Impfversorgung nach Transplantation bei SOT-Empfängern [124].

Beim Prätransplantationsscreening, bei dem u. a. ein virologisch-serologisches Testen auf latente Virusinfektionen durch CMV, Epstein-Barr-Virus (EBV), Hepatitis-B-Virus, Hepatitis-C-Virus und HIV erfolgt [125], muss auch unbedingt auf den Impfstatus geachtet werden. Das betrifft besonders Masern, Mumps, Röteln und Varizellen, die als Lebendimpfungen nur vor der Transplantation gegeben werden können/dürfen.

Prinzipiell müssen fehlende Impfungen mit inaktivierten Impfstoffen bis spätestens 2 Wochen vor Transplantation abgeschlossen sein, um mit einem adäquaten Impfansprechen rechnen zu können. Lebendimpfstoffe müssen spätestens 1 Monat vor Transplantation gegeben werden. Danach sind bei erwachsenen SOT-Empfängern aufgrund möglicher Komplikationen durch die Lebendimpfstämme keine Lebendimpfungen mehr möglich/empfohlen. Bei kindlichen SOT-Empfängern gibt es wenige Daten zur Anwendung von Lebendimpfungen gegen MMR oder Varizellen. Die vorhandenen Studien zeigten, dass es zu keinen Infektionen mit den attenuierten Impfstämmen gekommen ist [126]. Dennoch wird die Anwendung der Lebendimpfungen gegen MMR und VZV bei kindlichen SOT-Empfängern auf folgende Situationen beschränkt:

- Personen, die klinisch in gutem Allgemeinzustand sind, mehr als 1 Jahr nach einer Leber- oder Nierentransplantation stehen und bei denen mindestens 2 Monate keine akute Abstoßungsreaktion aufgetreten ist.
- Personen unter „Minimum-Immunsuppression und Minimum-Immunkriterien“, letztere definiert durch Lymphozytenzahl > 1500/µl und CD4^+^-T-Zellen > 700/µl bei Kindern < 6 Jahre; Lymphozytenzahl > 1000/µl; CD4^+^-T-Zellen > 500/µl bei Kindern > 6 Jahre, normale IgG-Werte und die Fähigkeit normale Impfantworten auf inaktivierte Impfstoffe zu machen. Unter Minimum-Immunsuppression versteht man Steroidgaben < 2 mg/kg KG/Tag oder einer kumulativen Dosis von < 20 mg/Tag; Tacrolimus < 8 ng/ml Zieltalspiegel und Cyclophosphamid < 100 ng/ml Zieltalspiegel.
- SOT-Empfänger müssen nach der Lebendimpfung engmaschig monitiert werden.

Nach der Transplantation soll ein Zeitabstand von mindestens 6 bis 12 Monaten eingehalten werden, weil davor mit einem zu geringen Ansprechen zu rechnen ist [127]. Eine Ausnahme stellen die inaktivierten Influenzaimpfstoffe dar, die frühsten 1 Monat nach Transplantation gegeben werden dürfen, und bei denen eine Revakzinierung 2 bis 3 Monate später erfolgen soll/kann [128].

Folgende impfpräventable Erkrankungen stellen ein besonderes Infektionsrisiko für alle SOT-Empfänger dar, gegen die vor Transplantation und ggf. danach unbedingt geimpft werden muss.

**COVID-19**: Das Risiko für eine schwere COVID-19-Erkrankung war zu Beginn der Pandemie hoch und die Sterberate lag bei hospitalisierten SOT-Empfängern bei 20 % [129]. Wenngleich sich die epidemiologische Situation im Hinblick auf die Pathogenität des Virus und die Immunkompetenz in der Bevölkerung deutlich verbessert haben, bleibt eine COVID-19-Impfung weiterhin bei diesen Patienten als sinnvoll einzustufen. Ähnliches gilt für **Influenza** und es konnte gezeigt werden, dass eine Influenzaimpfung den Schweregrad und damit das Mortalitätsrisiko deutlich reduzieren kann [127]. Cave: Die Lebendimpfung ist bei Kindern kontraindiziert.

**RSV** ist eine häufige Quelle (ca. 10 %) für schwere Infektionen des unteren Respirationstraktes und auch Ursache für Mortalität bei SOT – besonders Lungentransplantatempfänger. Eine Studie berichtet, dass 74 % der von RSV-Infektionen betroffenen Lungentransplantierten hospitalisiert werden mussten [130].

Auch **Pneumokokken**infektionen stellen bei SOT-Empfängern ein großes Problem dar und eine Impfung muss daher unbedingt vor geplanter Transplantation durchgeführt werden.

**Herpes Zoster:** SOT-Empfänger haben ein deutlich erhöhtes Risiko, Komplikationen bei primären Varizelleninfektionen zu erleiden. Etwa 2–3 % der SOT-Kandidaten sind Varizella-Zoster-Virus seronegativ und sollen bis spätesten 4 Wochen vor geplanter Transplantation mit der zweimaligen Lebendimpfung versorgt werden (Mindestabstand: 4 Wochen). Ebenso ist das Risiko für eine Herpes-Zoster-Reaktivierung bei SOT-Empfängern deutlich höher als in der gesunden Bevölkerung [131]. Daher sollen sowohl SOT-Kandidaten als auch SOT-Empfänger gegen Herpes Zoster mit dem inaktivierten Impfstoff geimpft werden. Da der Herpes-Zoster-Impfstoff erst ab 18 Jahren zugelassen ist, entspricht eine Anwendung bei Personen unter 18 Jahren einer Off-Label-Behandlung und bedarf einer Nutzen-Risiko-Abwägung und einer entsprechenden Aufklärung.

**Humane Papillomaviren** führen zu erhöhten Erkrankungsraten bei SOT-Empfängern [132]. Empfänger solider Organtransplantate haben ein höheres Risiko, eine HPV-Infektion zu bekommen, als Personen der allgemeinen Bevölkerung. Eine Infektion manifestiert sich als prämaligne Läsionen, Warzen oder Karzinome im Genitoanalbereich und die anfangs symptomlose Erkrankung macht eine rechtzeitige Diagnose ohne regelmäßiges Screening schwierig. Daher ist die prophylaktische HPV-Impfung für Frauen und Männer vor Transplantation sehr wichtig.

Invasive **Meningokokken**infektionen haben eine hohe Mortalitätsrate (15 %) und in einer Studie wurde berichtet, dass SOT-Empfänger ein 40-fach erhöhtes Risiko für invasive Verläufe haben [133]. Bei Nierentransplantationen wird zur Verhinderung von GvHD oftmals eine Behandlung mit Eculizumab (Komplemet-C5a-Inhibitor) angedacht. In diesen Fällen werden bereits vor Transplantation Impfungen gegen Meningokokken (ACWY und B) empfohlen.

**Hepatitis B und A**: Besonders gegen Hepatitis B sollen alle SOT-Kandidaten geimpft werden. Personen mit chronischen Leber- oder Nierenerkrankungen sollen bereits vor Transplantation geimpft werden (bei Lebererkrankung auch Hepatitis A).

Bei Personen, die auch nach Transplantation an **Reisetätigkeiten** denken, ist es entscheidend vor SOT gegen Erkrankungen zu impfen, die entweder nur als Lebendimpfstoffe vorliegen (z. B. Gelbfieber) und bei Immunsuppression kontraindiziert sind, oder gegen Erkrankungen, die bei ungenügendem Schutz einen tödlichen Verlauf nehmen können [124].

Details zu Impfempfehlungen bei SOT-Empfängern siehe die entsprechenden Impfschemata für die eingesetzten Immunsuppressiva.

### 5.8 Asplenie, Splenektomie

Man unterscheidet zwischen anatomischer und funktioneller Asplenie. Anatomische Asplenie bedeutet, dass eine Person keine Milz hat, entweder durch operative Entfernung oder in seltenen Fällen fehlt die Milz von Geburt an. Funktionelle Asplenie oder Hyposplenie bedeutet, dass die Milzfunktion beeinträchtigt ist. Dies wird i. d. R. durch angeborene Störungen, systemische Erkrankungen wie Sichelzellenanämie, Thalassämie, Leberzirrhose, hämatoonkologische Erkrankungen, Autoimmunität oder Infektionen (wie HIV, Malaria) oder SARS-CoV-2-Infektionen verursacht [134].

Menschen mit anatomischer oder funktioneller Asplenie haben lebenslang ein **erhöhtes Risiko für schwere bakterielle Infektionen**, insbesondere mit bekapselten Bakterien, wie ***Streptococcus pneumoniae*, *Neisseria meningitidis*** **und *Haemophilus influenzae Typ B***. Eine **Influenzainfektion** stellt besonders durch die Gefahr einer bakteriellen Superinfektion für Menschen ohne Milz ein erhöhtes Risiko dar. **SARS-CoV-2-Infektionen** wiederum können Ursache für die Entstehung von Hyposplenismus sein, da SARS-CoV‑2 einen starken Tropismus für die Milz zeigt. Durch den Funktionsverlust der Milz kann eine SARS-CoV-2-Infektion einen sehr schlechten Verlauf nehmen [134].

Das Infektionsrisiko nach einer Splenektomie bleibt lebenslang bestehen. Allerdings stellen die Jahre unmittelbar **nach der Splenektomie das größte Risiko** dar, da fast **30 % der Infektionen innerhalb des ersten Jahres und 50 % innerhalb der ersten zwei Jahre nach der Splenektomie auftreten.**

Bei einer geplanten Splenektomie sollten die empfohlenen Impfungen idealerweise mindestens 2 Wochen vor der Operation abgeschlossen sein.

Nach einer Notfallsplenektomie sollten alle ausstehenden Impfungen idealerweise mindestens 2 Wochen nach der Operation und vor Spitalentlassung verabreicht werden, können aber auch gegeben werden, sobald die Person stabil ist. Prinzipiell sind die Antikörperantworten (insbesondere auf Pneumokokkenimpfstoffe) besser, wenn diese Impfstoffe 2 Wochen nach der Operation verabreicht werden. Wenn jedoch Bedenken bestehen, dass die Person nicht zurückkehrt oder nach der Splenektomie für die Nachsorge nicht mehr zur Verfügung steht, können die Impfungen verabreicht werden, sobald die Person stabil ist und vor der Entlassung aus dem Krankenhaus steht.

Personen mit nichtchirurgischer Asplenie oder Hyposplenie sollten alle altersempfohlenen Impfungen erhalten, sobald die eingeschränkte Milzfunktion erkannt wird.

Hinsichtlich des prinzipiellen Ansprechens auf Impfungen bei Asplenie oder Hyposplenie ist zu erwähnen, dass aufgrund der geringeren Anzahl von B‑Gedächtniszellen und langlebigen Plasmazellen die Impfantworten niedriger sein können bzw. es zu einem rascheren Abfall der Antikörper kommen kann – das betrifft nicht nur Impfungen mit Polysaccharidantigenen (wie Pneumokokken). Dementsprechend sind wiederholte Auffrischungsimpfungen (und ggf. Impferfolgskontrollen) nötig. Das Fehlen der Milz per se stellt noch keine Kontraindikation für Lebendimpfungen dar – allerdings kann die zugrunde liegende Erkrankung (z. B. immunmediierte Erkrankung) sehr wohl zur Kontraindikation für Lebendimpfungen führen ([134]; Tab. 16).

**Tab. 16:** Beispiele für Hauptursachen für Asplenie und Hyposplenismus. (Adaptiert nach [134, 135])

**Ursachen von Asplenie**	
*Angeborene Asplenie*	Frühgeburt (kann eine Ursache für Asplenie sein)
	Isolierte angeborene Asplenie, syndromale Erkrankungen mit Asplenie (z. B. Ivemark-Syndrom)
	Hypoparathyreoidismus-Retardierung-Dysmorphismus
	Zyanotische Herzkrankheit
	Fanconi-Anämie
*Chirurgische Splenektomie*	–
**Ursachen von Hyposplenismus/funktioneller Asplenie**	
*Magen-Darm-Erkrankungen*	Zöliakie
	Entzündliche Darmerkrankungen
	Primäre eosinophile gastrointestinale Erkrankungen
	Autoimmungastritis
*Lebererkrankungen*	Chronisch aktive Hepatitis
	Leberzirrhose und portale Hypertonie
	Primär biliäre Cholangitis
*Onkologische und hämatoonkologische Erkrankungen*	Hämolytische Anämien; Hämoglobinbildungsstörungen/Thalassämie
	Akute Leukämie
	Chronische myeloproliferative Erkrankung
	Idiopathische thrombozytopenische Purpura
	Milzkrebs (primär und sekundär)
*Immunvermittelte Erkrankungen oder Immunschwächen*	Systemischer Lupus erythematodes
	Rheumatoide Arthritis
	Glomerulonephritis
	Sarkoidose
	Vaskulitis
*Infektionskrankheiten*	HIV-Infektion und AIDS
	Pneumokokkenmeningitis
	Malaria
	SARS-CoV-2-Infektion
*Iatrogene Ursachen*	Hoch dosierte Steroide
	Milzstrahlentherapie
*Milzgefäßveränderungen*	Thrombose der Milzarterie
	Milzvenenthrombose
*Andere Ursachen*	Amyloidose
	Hyposplenismus aufgrund des Alterns
	Hypopituitarismus

*AIDS* erworbenes Immunschwächesyndrom, *HIV* humanes Immundefizienzvirus, *SARS-CoV‑2* „severe acute respiratory syndrome Coronavirus 2“

### 5.9 Angeborene („primäre“) Immundefekte

Genetisch determinierte angeborene Fehler des Immunsystems („inborn errors of immunity“, IEI) umfassen heutzutage mehr als 550 genetisch definierte Entitäten [136]. Obwohl die einzelnen Erkrankungsentitäten für sich gesehen selten sind, machen sie in der Summe eine beträchtliche Anzahl der Erkrankungen aus. Epidemiologische Analysen aus den USA gehen von einer zusammengefassten Krankheitsprävalenz klinisch relevanter IEI von 1:1500 bis 1:2000 aus und somit überschreitet die Gesamtzahl die Definition einer seltenen Erkrankung (i.e. seltener als 1:2000). Somit ist es von Bedeutung, auch die Impfungen und impfpräventable Infektionsprophylaxe bei angeborenen Immundefekten zu diskutieren und Empfehlungen auszusprechen.

Zurzeit können die IEI in zehn Gruppen zusammengefasst werden, die es erlauben, das Impfansprechen vorherzusagen bzw. die möglichen Impfstrategien einzuteilen (Klassifikation [2024] der primären Immundefekte gemäß der International Union of Immunological Societies [IUIS] [136]):

I. Immundefekte mit Störung zellulärer und humoraler Immunität
II. Kombinierte Immundefekte mit syndromalen Merkmalen
III. Vorwiegende Antikörperdefekte
IV. Erkrankungen mit Immunregulationsstörung
V. Defekte der Phagozytenzahl/-funktion
VI. Defekte intrinsischer/angeborener Immunität
VII. Autoinflammatorische Erkrankungen
VIII. Komplementdefekte
IX. Knochenmarkversagen
X. Phänokopien von PID

Impfempfehlungen, welche eventuell bereits im 1. Lebensjahr von den allgemeinen Impfempfehlungen abweichen, können aufgrund des in Österreich etablierten Neugeborenenscreenings auf schwere (kombinierte) Immundefekte (S[C]ID) frühzeitig personalisiert und eventuelle Kontraindikationen für Lebendimpfungen eingeschätzt werden (Tab. 17).

**Tab. 17:** Typische Beispiele für angeborene Immundefekte, geordnet nach den IUIS-2024-Gruppen, mit Infektionsrisiko [136]

**Typ nach IUIS**	**Betroffene immunologische Achsen**		**Name**	**Abkürzung**	**ISP-Grad**^**a**^	**Infektionsrisiko**	**Besondere Hinweise/Risiken (konkrete Impfempfehlung siehe Abschnitt 6)**
I – ID mit Störung zellulärer und humoraler Immunität	T Zellen	T‑B−NK−	Adenosindesaminase Mangel (SCID)	ADA	III	Hoch	Keine Lebendimpfungen; inaktivierte Impfungen möglich, aber Wirkung oftmals eingeschränkt. Bei Ig-Therapie inaktivierte Influenzaimpfung möglich; postexpositionell (z. B. Masern) Verabreichung von spezifischem Ig
		T‑B + NK−	Common-gamma-chain deficiency (SCID)	γc	III	Hoch	
		T‑B + NK+	IL-7‑α receptor deficiency (SCID)	IL7Ra	III	Hoch	
	v. a. CD 8	–	Weitere Beispiele: DOCK8-Def, ZAP70-Def	–	II–III	Hoch	
II – ID mit syndromalen Merkmalen	T Zellen & Syndrome	–	Wiscott-Aldrich Syndrome	WAS	III	Hoch	Keine Lebendimpfungen; inaktivierte Impfungen möglich, aber Wirkung oftmals eingeschränkt. Bei Ig-Therapie inaktivierte Influenzaimpfung möglich; postexpositionell (z. B. Masern) Verabreichung von spezifischem Ig
		–	Ataxia teleangiectasia	AT	III	Hoch	
		–	DiGeorge-Syndrom	DGS	II–III	Moderat–hoch	Lebendimpfungen abhängig von ID/ISP-Grad; inaktivierte Impfungen möglich, aber Wirkung oftmals eingeschränkt. Bei Ig-Therapie inaktivierte Influenzaimpfung möglich; postexpositionell (z. B. Masern) Verabreichung von spezifischem Ig
III – Vorwiegend AK-Defekte	–	B−	X‑linked agammaglobulinemia	XLA	III	Hoch	Keine Daten für Rotavirusimpfung; Routine-Totimpfstoffe können gegeben werden, wenn keine Ig-Therapie, Wirksamkeit aber unsicher
		B+	Common variable immunodeficiency	CVID	II–III	Moderat–hoch	Impfung vor allem gegen bekapselte Bakterien wichtig (Meningokokken, Pneumokokken, Haemophilus influenzae); Impfungen auch unter Ig-Therapie teile sinnvoll (z. B. Influenza, COVID-19, FSME)
		–	Selective IgA deficiency	sIgA Mangel	II	Gering	Fast alle Impfungen möglich. Besonders empfohlen werden Impfung gegen Pneumokokken, wegen schwererem Krankheitsverlauf; Impfungen über die Schleimhäute wie etwa Rotavirusimpfung, Influenzalebendimpfung sind kontraindiziert
		–	Subklassenmangel	–	I–II	Gering–moderat	Alle Impfungen möglich. Besonders empfohlen werden Impfung gegen Pneumokokken, Influenza, Meningokokken und Haemophilus influenzae
IV – Erkrankungen mit Immunregulationsstörung	–	–	Autoimmunes lymphoproliferatives Syndrom	ALPS	I	Gering	Prinzipiell alle Impfungen möglich. Achtung bei immunsuppressiven Therapien siehe Abschnitt 6
	–	–	Autoimmune polyendocrinopathy with candidiasis and ectodermal dystrophy = autoimmunes polyglanduläres Syndrom Typ 1 (APS Typ 1)	APECED	III	Hoch	Keine Gelbfieber- und nasale Influenzaimpfung. Andere Lebendimpfungen möglich, aber nicht bei hohen anti-IFN-AK-Spiegeln bzw. medikamentöser Immunsuppression. APECED ist oft mit funktioneller Asplenie assoziiert, daher Impfungen gegen bekapselte Bakterien wie Pneumokokken, Meningokokken und HiB wichtig. Jährliche Influenza Impfung
V – Defekte der Phagozytenzahl/-funktion	–	Neutropenie	Shwachman-Diamond Syndrome	–	I	Gering	Alle inaktivierten und Lebendimpfungen sind sicher und wirksam
	–	Ohne Neutropenie	Chronische Granulomatöse Erkrankung	CGD	II–III	Moderat–hoch	KI für die folgenden Lebendimpfungen: Gelbfieber-, orale Polio‑, Typhus- und nasale Influenzaimpfung
	–	–	Leukozyten Adhäsionsdefekt Typ 1	LAD 1	III	Hoch	i. d. R. keine Lebendimpfungen; alle inaktivierten Impfstoffe gut und wichtig
	Phagozytenmotilität	–	Cystische Fibrose	CF	I–II	Moderat	Jährliche Influenza‑, COVID-19-, Pneumokokkenimpfung, sowie obligate Hepatitis-A- und -B-Impfung
VI – Defekte intrinsischer/angeborener Immunität	–	Viral	Interferon-α-Rezeptor-1-Defizienz	IFNAR1 Def	III	Hoch	KI: Gelbfieber- und Masern- sowie nasale Influenzaimpfung
			Interferon-regulierender Faktor-7/9-Defekte	IRF7 & IRF 9	III	Hoch	KI: Gelbfieber- und Masern- sowie nasale Influenzaimpfung. Da schwere Influenzainfektionen vorkommen können: jährliche Influenzaimpfung sowie alle anderen inaktivierten Impfungen empfohlen
	–	Bakteriell	IL-12-Achsendefekte (Mendelian Susceptibility to Mycobacterial Disease)	MSMD	II–III	Moderat–hoch	KI: Impfungen mit lebenden Bakterien (orale Typhusvakzine und BCG). Virale Lebendimpfstoffe erlaubt sowie auch alle inaktivierten Impfstoffe
	–	–	Prädisposition für u. a. Pilzerkrankungen	STAT1 & IL-17R	II–III	Moderat–hoch	KI: Gelbfieber‑, MMR- sowie nasale Influenzalebendimpfung v. a. bei Patienten mit STAT1-LOF-Mutationen. Wichtig ist Aufbau eines Impfschutzes gegenüber saisonaler Influenza und Pneumokokken
VII – Autoinflammatorische Erkrankungen	–	–	Familiäres Mittelmeerfieber	FMF	I	Gering	Im Prinzip alle Impfungen möglich, jedoch mögliche Einschränkungen bei Lebendimpfstoffen durch verabreichte Medikation (z. B. bei IL-1-Biologika). Wichtig: Impfungen gegen saisonale Influenza und Pneumokokken
			TNFR-assoziierte periodische Fiebersyndrome	TRAPS	I	Gering	Prinzipiell sind alle Impfungen indiziert, Einschränkungen können für Lebendimpfungen bei Therapie mit IL-1-Inhibitoren entstehen. Saisonale Influenza- sowie Pneumokokkenimpfung dringend angezeigt (verhindert TRAPS-Ausbruch)
			Arthritis, Pyoderma gangraenosum, Myositis, akute Phase Proteine	PAPA-Syndrom	I	Gering	Prinzipiell alle Impfungen möglich, jedoch abhängig von Therapie
VIII – Komplementdefekte	–	C5-C9	–	–	I–III	Moderat	Besonders anfällig für Infektionen mit bekapselten Bakterien (Pneumo- und Meningokokken, HiB), siehe Abschnitt 6
		Properdin Def	–	–	I–III	Moderat	
		MBL Def	–	–	I	Gering	
IX – Knochenmarkversagen	–	–	Fanconi Anämie	FA	I–II	Moderat	HPV-Impfungen zwingend ab 13. Lebensjahr; des Weiteren sind Impfungen gegen Hepatitis B, saisonale Influenza, Pneumokokken und Haemophilus influenzae dringend indiziert
	–	–	Dyskeratosis congenita	DC	I	Gering	HPV-Impfungen zwingend ab 13. Lebensjahr; des Weiteren sind Impfungen gegen Hepatitis B, saisonale Influenza, Pneumokokken und HiB dringend indiziert
X – Phänokopien von PID	–	–	Chronische mukokutane Candidiasis durch anti-IL-17 Autoantikörper	CMC	I–II	Moderat	KI: Gelbfieber-, nasale Influenzaimpfung, sowie MMR v. a. bei Patienten mit Phänokopie STAT1-LOF-Mutationen. Wichtig ist Aufbau eines Impfschutzes gegenüber saisonaler Influenza und Pneumokokken
	–	–	Adult-onset Immundefizienzsyndrom mit Anfälligkeit für Mykobakterien durch anti-IFN-γ-Autoantikörper	AOIDSM	II	Moderat	KI: Impfungen mit bakterielle Lebendimpfstoffe. Virale Lebendimpfstoffe sind erlaubt sowie auch alle inaktivierten Impfstoffe

*AK* Antikörper, *BCG* Bacillus Calmette-Guérin, *COVID-19* Coronavirus disease 2019, *HiB Haemophilus influenzae* Typ B, *HPV* humanes Papillomavirus, *ID* Immundefizienz, *IFN* Interferon, *Ig* Immunglobulin, *IL* Interleukin, *ISP* Immunsuppression, *IUIS* International Union of Immunological Societies, *KI* Kontraindikation, *MMR* Masern-Mumps-Röteln-Dreifachimpfung, *PID* primäre Immundefekte, *St.p.* Status post, *STAT1-LOF* Loss-of-Function-Mutation im *STAT1*-Gen, *TNFR* Tumornekrosefaktorrezeptor

### 5.10 Immunsupprimierte Kinder (sekundäre Immundefizienz)

Kinder, die infolge einer immunsuppressiven oder immunmodulatorischen Therapie eine sekundäre Immundefizienz entwickeln, tragen im Vergleich zu gesunden Gleichaltrigen ein erhöhtes Risiko für bakterielle, virale und opportunistische Infektionen, wie Pneumokokken, Influenza, Varizellen, Masern oder Hepatitis B, die nicht selten mit schwereren Verläufen und gehäuften Komplikationen einhergehen. Impfungen sind bei immunsupprimierten Kindern jedoch häufig mit Zurückhaltung und Unsicherheit verbunden, was nicht selten zu unvollständigen Impfserien sowie verzögerten oder gänzlich versäumten Indikationsimpfungen führt [137].

Impfungen bei Kindern mit sekundärer Immundefizienz erfordern eine sorgfältige Differenzierung nach Art und Ausmaß der Immunsuppression. Inaktivierte Impfstoffe sind in dieser Population im Allgemeinen sicher und sollten konsequent entsprechend dem österreichischen Impfplan [6] eingesetzt werden, um Standard- und Indikationsimpfungen zu vervollständigen. Lebendimpfstoffe sind unter intensiver zytotoxischer Chemotherapie und bei ausgeprägter Immunsuppression kontraindiziert und können erst nach dokumentierter Immunrekonstitution wieder in Betracht gezogen werden. Bei Therapien mit geringer oder fehlender systemischer Immunsuppression, wie BRAF- und MEK-Inhibitoren ohne weitere immunsuppressive Kombinationspartner oder stabile Tacrolimusmonotherapie Jahre nach Lebertransplantation, sind Lebendimpfungen nach individueller Risikoabwägung und unter strenger Einhaltung definierter Sicherheitskriterien möglich. Für alle diese Konstellationen gilt, dass Impfentscheidungen idealerweise im interdisziplinären Team getroffen, Eltern transparent aufgeklärt und Impfantworten – wo immer möglich – serologisch überprüft werden sollten [137, 138]. Weiters kann die Messung der Impfantikörperkonzentrationen vor und nach Impfung als Surrogatmarker für die Funktionalität des Immunsystems (B-Zell-Funktion und T‑Zell-Aktivierung) herangezogen werden (diagnostische Impfung).

Auch im Kindesalter spielen für eine immunsuppressive Therapie systemische Glukokortikoide, konventionelle Immunsuppressiva wie Methotrexat, Azathioprin oder Mycophenolat mofetil, Calcineurininhibitoren sowie Biologika und andere zielgerichtete Therapien (z. B. BRAF-, MEK- und JAK-Inhibitoren) eine zentrale Rolle [137, 138].

Wie bei Erwachsenen auch sollten Impfungen/Impfserien – soweit möglich – vor Beginn einer immunsuppressiven Therapie abgeschlossen werden, da zu diesem Zeitpunkt die Immunantwort jedenfalls zuverlässig ist. Inaktivierte Impfstoffe gelten bei Kindern mit sekundärer Immundefizienz im Allgemeinen als sicher und können auch unter laufender Therapie eingesetzt werden. Sie führen nach derzeitigem Wissensstand weder zu Schüben der Grunderkrankung noch zu systematischen Verschlechterungen des klinischen Zustands. Die Empfehlungen der Société Internationale d’Oncologie Pédiatrique (SIOP) zur Immunisierung von Kindern mit Krebs [104] unterstreichen, dass inaktivierte Impfstoffe grundsätzlich verabreicht werden sollen, sofern keine akute Kontraindikation besteht. Gleichwohl ist zu berücksichtigen, dass die Immunantwort abgeschwächt sein kann. Insbesondere bei Impfstoffen, bei denen klar definierte Antikörperkonzentrationen für ein Schutzkorrelat existieren (z. B. Hepatitis B, Tetanus, Masern, Varizellen), sind Impferfolgskontrollen sinnvoll (siehe Tab. 5), um den Impferfolg und die Protektionsdauer abzuschätzen und gegebenenfalls zusätzliche Impfdosen einzuplanen [139].

Die Empfehlungen der European Alliance of Associations for Rheumatology (EULAR)/Paediatric Rheumatology European Society (PReS) für Kinder mit rheumatologischen Grunderkrankungen unterscheiden bei MMR- und VZV-Lebendimpfung zwischen erster Impfung und Wiederholungsimpfung. Wiederholungsimpfungen können nach Nutzen-Risiko-Abwägung unter Methotrexat-Therapie durchgeführt werden. In geringerem Ausmaß gilt dies auch unter Therapie mit TNF-α‑, IL-1- und IL-6-Inhibitoren [140]. Lebendimpfstoffe wie MMR und Varizellen sind während einer Chemotherapie kontraindiziert und ihre Wiederaufnahme ist frühestens mehrere Monate nach Therapieende vorgesehen, wenn sich die Lymphozytenzahlen erholt haben und eine stabile hämatologische Rekonstitution (> 1500/µl für Kinder < 6 Jahre bzw. 1000/µl für Kinder > 6 Jahre) vorliegt [104]. Dies kann mindestens 3 bis 6 Monate, teils bis zu 12 Monate dauern; bei B‑Zell-depletierenden Antikörpertherapien (z. B. Rituximab) werden deutlich längere Abstände gefordert [104].

Bei BRAF- und MEK-Inhibitoren (z. B. Dabrafenib, Trametinib, Selumetinib) werden keine opportunistischen Infektionen beschrieben [98]. MEK-Inhibitoren weisen eine komplexe Immunmodulation auf, die sich aber nicht zwingend immunsuppressiv auswirkt [141]. Inaktivierte Impfstoffe können daher uneingeschränkt verabreicht werden, Lebendimpfstoffe (MMR, Varizellen) allerdings nur nach individueller Nutzen-Risiko-Abwägung sowie unter der Voraussetzung stabiler klinischer und laborchemischer Verhältnisse, häufig ergänzt durch Impferfolgskontrollen [137].

Für verschiedene Tyrosinkinase-, „mechanistic target of rapamycin“(mTOR)- oder JAK-Inhibitoren wurde ein teils erheblich erhöhtes Risiko für bakterielle, virale und opportunistische Infektionen beschrieben [142]. Hier sind Lebendimpfstoffe – analog zur intensiven Chemotherapie – in aller Regel kontraindiziert. Da für viele dieser neueren Wirkstoffe kaum Daten zum Impfansprechen vorliegen, ist bei der Planung von Impfungen eine enge interdisziplinäre Abstimmung sinnvoll. Wo es möglich ist, können serologische Kontrollen, Lymphozytensubsettypisierungen und Bestimmung der Immunglobulinspiegel zur Einschätzung der individuellen Immunkompetenz herangezogen werden.

Nach solider Organtransplantation (siehe Abschnitt 5.7), insbesondere nach Lebertransplantation, stehen die Kinder meist unter einer calcineurininhibitorbasierten Immunsuppression (vor allem Tacrolimus). Mehrere Kohorten- und Registerstudien konnten zeigen, dass MMR- und Varizellenimpfungen bei sorgfältig ausgewählten pädiatrischen Leber- und Nierentransplantierten – meist Jahre nach Transplantation, unter niedriger Calcineurininhibitormonotherapie, stabiler Graftfunktion und adäquaten Lymphozyten- und Immunglobulinwerten – sowohl sicher als auch immunogen sein können [143]. Insgesamt waren Varizellenimpfungen etwas weniger immunogen als in der Normalbevölkerung, was die Empfehlung zu serologischen Kontrollen und ggf. Wiederholungsimpfungen stützt (Tab. 18).

**Tab. 18:** Immunsuppression/sekundäre Immundefizienz im Kindes- und Jugendalter und impfrelevante Konsequenzen [2, 126, 143–146]

**ISP-Grad**	**Typische pädiatrische Beispiele (Diagnosen/Therapien)**	**Impfrelevante Konsequenzen**
*I – keine/geringe Immunsuppression*	Kurzzeitige systemische Glukokortikoidtherapie: Prednisolon < 20 mg/Tag **oder** < 2 mg/kg/Tag, Dauer < 2 Wochen; alternierende Gabe	**Alle inaktivierten und Lebendimpfstoffe** (MMR, VZV) wie bei Gesunden möglich
	Inhalative, topische, intraartikuläre Steroide	Keine routinemäßigen Impferfolgskontrollen notwendig (Ausnahme: besondere Fragestellungen, z. B. Hepatitis B vor Ausbildung zu einem Risikoberuf)
	Autoimmun-/entzündliche Erkrankung **ohne** immunsuppressive/systemische Therapie (z. B. JIA nur unter NSAR, milde CED unter 5‑ASA)	
	DMARDs mit praktisch keiner systemischen ISP: Mesalazin, Sulfasalazin, Hydroxychloroquin, 5‑ASA	
	HIV-infizierte Kinder mit CD4 ≥ 500/µl bzw. > 25 % CD4	
	Kinder mit onkologischen Erkrankungen > 3 (–6) Monate nach Ende der Chemotherapie, in kompletter Remission, ohne laufende Immunsuppression	
	Topisches Tacrolimus (Dermatologie)	
	Zielgerichtete Therapien ohne nachweisbare relevante Immunsuppression, z. B. **MEK-Inhibitoren (Trametinib, Selumetinib)** in Monotherapie, sofern keine zusätzliche immunsuppressive Kombination und keine auffällige Infektionsanfälligkeit	
*II – leichte bis mittelgradige Immunsuppression*	Systemische Steroide < 20 mg Prednisolonäquivalent/Tag **>** **2 Wochen** oder Depotsteroidgabe	**Inaktivierte Impfstoffe:** uneingeschränkt empfohlen, ggf. mit Impferfolgskontrolle (z. B. Hepatitis B, Tetanus)
	> 20 mg/Tag für < 2 Wochen (Lebendimpfungen erst ≥ 2 Wochen nach Ende)	**Lebendimpfstoffe (MMR, VZV):** im Regelfall **möglich**, aber nur bei stabiler klinischer Situation und sorgfältiger Risiko-Nutzen-Abwägung; Impferfolgskontrollen (z. B. Masern/VZV) sinnvoll, wenn verfügbar
	„Low-dose“-Immunsuppressiva: Methotrexat < 0,4 mg/kg/Woche (z. B. JIA) Azathioprin < 3 mg/kg/Tag 6‑Mercaptopurin < 1,5 mg/kg/Tag (z. B. CED)	
	Asymptomatische HIV-Infektion mit CD4 200–499/µl bzw. 15–25 % bei Kindern	
	Anatomische/funktionelle Asplenie (z. B. Splenektomie, Sichelzellanämie)	
	Fortgeschrittene chronische Nieren‑/Lebererkrankung, Diabetes mit Organschäden	
*IIIa – schwere Immunsuppression (stabile/chronische Phase)*	Systemische Steroide > 20 mg/Tag **oder** > 2 mg/kg/Tag **>** **2 Wochen** (Kinder < 10 kg)	**Inaktivierte Impfstoffe:** empfohlen; Impfansprechen häufig reduziert → Impferfolgskontrollen nach Möglichkeit (Hepatitis B, Tetanus, Masern/VZV etc.)
	Kombination mehrerer Immunsuppressiva (z. B. MTX + TNF-α-Inhibitoren bei JIA/CED)	**Lebendimpfstoffe:** grundsätzlich **kontraindiziert**, können aber in **streng selektionierten Einzelfällen** (z. B. pädiatrische Leber‑/Nierentransplantierte unter niedriger Tacrolimusmonotherapie, stabile Graftfunktion) in spezialisierten Zentren nach Studienlage [126, 143–146] erwogen werden
	Biologika mit relevanter systemischer Wirkung: TNF-α-Inhibitoren, IL-6-/IL-17-Inhibitoren, JAK-Inhibitoren, mTOR-Inhibitoren etc. (Erhaltungsphase)	
	Stabile Phase nach SOT (> 12 Monate), unter **Calcineurininhibitormonotherapie (z.** **B. Tacrolimus)** in niedriger bis mittlerer Dosis, ohne Organabstoßung und mit guter Organfunktion (typische Situation pädiatrischer Lebertransplantations-Zentren, die VZV/MMR in Einzelfällen impfen)	
	HSCT > 2 Jahre mit noch milder Erhaltungsimmunsuppression/GvHD Grad I–II	
*IIIb – sehr schwere/tiefe Immunsuppression (akute/instabile Phase)*	Laufende **intensive zytotoxische Chemotherapie** bei ALL/AML, Lymphomen, Hochrisikoneuroblastom, Sarkomen etc. sowie die ersten Monate nach Ende der Chemotherapie (je nach Regime ≥ 3–6, teils 12 Monate)	**Lebendimpfstoffe: absolute Kontraindikation**
	Allogene SZT ≤ 2 Jahre nach Transplantation und/oder unter Immunsuppression/GvHD	**Inaktivierte Impfstoffe:** können – meist mit reduziertem Ansprechen – gegeben werden, Priorität auf Influenza, Pneumokokken, HiB, Meningokokken, Hepatitis B etc.; möglichst Impfung in Phasen geringerer Immunsuppression, wenn planbar
	SOT ≤ 12 Monate nach Transplantation oder bei Abstoßungsbehandlung (hoch dosierte Steroide, Antithymozytenglobulin, Rituximab etc.)	Serologische Kontrollen und ggf. Wiederholungsimpfungen besonders wichtig
	Therapie mit stark T‑/B-Zell-depletierenden Antikörpern in den ersten 6–12 Monaten (z. B. Rituximab, Alemtuzumab, Anti-CD52-Antikörper)	
	Schwere kombinierte oder andere ausgeprägte primäre Immundefekte (z. B. SCID)	

*5‑ASA* 5-Aminosalicylsäure, *ALL* akute lymphatische Leukämie, *AML* akute myeloische Leukämie, *CED* chronisch-entzündliche Darmerkrankung, *DMARDs* krankheitsmodifizierende Antirheumatika, *ggf.* gegebenenfalls, *GvHD* Graft-versus-Host-Erkrankung, *HiB Haemophilus influenzae* Typ B, *HIV* humanes Immundefizienzvirus, *HSCT* hämatopoetische Stammzelltransplantation, *IL* Interleukin, *ISP* Immunsuppression, *JAK* Januskinase, *JIA* juvenile idiopathische Arthritis, *MEK* „mitogen-activated protein kinase“, *MMR* Masern-Mumps-Röteln, *mTOR* „mechanistic target of rapamycin“, *MTX* Methotrexat, *NSAR* nichtsteroidale Antirheumatika, *SCID* schwere kombinierte Immundefekte, *SOT* solide Organtransplantation, *SZT* Stammzelltransplantation, *TNF* Tumornekrosefaktor, *VZV* Varicella-Zoster-Virus

### 5.11 Biologika in Schwangerschaft und Stillzeit – Impfen von Kindern behandelter Mütter

Die Möglichkeit, Säuglinge von Müttern zu impfen, die während der Schwangerschaft mit Biologika behandelt werden, hängt vom immunsupprimierenden Biologikum und der Art des zu applizierenden Impfstoffs ab, sowie dem Zeitpunkt der maternalen Therapie (vor oder nach der 20. Schwangerschaftswoche [147]). Tab. 19 fasst die unterschiedlichen Szenarien und Biologikatherapien zusammen [148]*. *Die RSV-Prophylaxe mit monoklonalen Antikörpern kann gemäß allgemeinen Empfehlungen verabreicht werden – es sind keine zusätzlichen Intervalle einzuhalten.

**Tab. 19:** Verabreichung von inaktivierten und Lebendimpfstoffen an Säuglinge, die biologischen immunsuppressiven Therapien in utero ausgesetzt waren [6, 83]

**Alter**	**Inaktivierte Impfstoffe**	**Lebendimpfstoffe für Säuglinge, die in utero biologischen Immunsuppressiva ausgesetzt waren (Ausnahme: Anti-CD20-Antikörper)**	Lebendimpfstoffe für Säuglinge, die in utero Anti-CD20-Antikörpern ausgesetzt waren*
< 6 Monate	Ja, nach österr. Impfplan	Ja, nach österr. Impfplan (auch Rotavirusimpfstoff)	Nein**
≥ 6 Monate	Ja, nach österr. Impfplan	Ja, nach österr. Impfplan	Ja, nach österr. Impfplan

* Anmerkungen: Rituximabtherapie der Mutter während Schwangerschaft führt zu Depletion von B‑Zellen bei Mutter und Kind. **Maternale monoklonale Antikörper persistieren etwa 6 Monate im Säugling**. Ein Intervall von 6–12 Monaten bei Mutter und Kind vor Applikation einer Lebendimpfung soll eingehalten werden, anderenfalls ist eine immunologische Kontrolle empfohlen.

** Ja, wenn die Therapie bis zur 20. Schwangerschaftswoche verabreicht wurde [147].

Während der Stillzeit applizierte Biologika haben i. d. R. keinen wesentlichen Einfluss auf die Impffähigkeit und die Impfantwort beim Kind. Die Übertragung monoklonaler Antikörper durch die Muttermilch ist als minimal einzustufen, und mögliche geringe Mengen werden im Gastrointestinaltrakt verdaut/inaktiviert, sodass Säuglinge/Kinder von Müttern unter monoklonaler Antikörpertherapie sowohl mit Lebendimpfungen (Ausnahme: Rotavirus bei maternaler Anti-CD20-Antikörpertherapie in der Schwangerschaft) als auch inaktivierten Impfstoffen entsprechend dem Impfkalender des österreichischen Impfplans geimpft werden können (Ausnahme: Gelbfieberimpfung; [149]).

## 6 Praktisches Vorgehen bei Immunsuppression: konkrete Impfempfehlungen nach Substanzklassen/Therapiearten

In den folgenden Abschnitten soll auf Substanzklassen bzw. Therapiearten eingegangen werden, bei denen typischerweise eine Immunsuppression zu erwarten ist und bei denen konkrete Infektionsrisiken entstehen, gegen die ein Impfschutz bestehen bzw. aufgebaut werden soll/muss.

Das erforderliche Impfprogramm sollte im Optimalfall spätestens 2 bis 4 Wochen vor Therapiebeginn durchgeführt werden. Kann dieses Intervall aufgrund der Krankheitsaktivität nicht eingehalten werden, kann *das Schema mit ****inaktivierten – nicht aber mit Lebend-****Impfstoffen* auch während immunsuppressiven Therapien ausgeführt werden (siehe 6.2).

Die Beurteilung der Impffähigkeit dieser Personen kann immer nur im Zusammenspiel zwischen Beurteilung der Grunderkrankung und Anwendung der Medikamentengruppe getroffen werden.

### 6.1 Impfschema für lebend-attenuierte Impfstoffe

Lebendimpfungen sind **unter** immunsupprimierender Therapie vom Grad III prinzipiell kontraindiziert, weswegen die Immunität gegenüber diesen impfpräventablen Erkrankungen (z. B. MMR, Varizellen) **VOR Immuntherapie überprüft werden soll;** ggf. sollen **4 bis 6 Wochen vor Therapiebeginn** notwendige Lebendimpfungen gegeben werden.

Wo immer es inaktivierte Alternativimpfstoffe gibt, sind diese vorzuziehen (Tab. 20).

**Tab. 20:** Lebend-attenuierte Impfstoffe

**Impfungen**	**Grundimmunisierung**	**Auffrischung (routinemäßig)**	**Alternativer inaktivierter Impfstoff**
**Rotavirus** (ab 6. Lebenswoche bis 24./32. Lebenswoche)	2 bzw. 3 Dosen je nach Impfstoff	Nein	Nein
**MMR** (ab (6.) 9. Lebenmonat)	2 Dosen, Abstand ≥ 4 Wochen	Nein	Nein
**Varizellen** (ab 12. Lebensmonat)	2 Dosen, Abstand ≥ 4 Wochen	Nein	Nein, ≥ 18 Jahre inaktivierter Herpes-Zoster-Impfstoff (siehe österr. Impfplan)
**MMRV** (ab 12. Lebensmonat)	2 Dosen, Abstand ≥ 4 Wochen	Nein	Nein
**Influenza** LAIV (2–18 Jahre)	Bis vollendetes 9. Lebensjahr 2× Abstand 4 Wochen	Jährlich	Ja
**Reiseimpfungen**			
*Chikungunya*	1 Dosis	Derzeit nicht bekannt	Ja
*Cholera*	1 Dosis	Derzeit nicht bekannt	Ja
*Dengue*	2 Dosen, Abstand ≥ 3 Monate	Derzeit nicht bekannt	Nein
*Gelbfieber*	1 Dosis	Einmalig nach 10 Jahren	Nein
*Mpox (nicht replizierend)*	2 Dosen, Abstand ≥ 4 Wochen	2–5 Jahre	Nein
*Typhus*	3 Kapseln (Tage 1, 3, 5), nüchtern	Nach 3 Jahren	Ja

*LAIV* „live attenuated influenza vaccine“, *MMR* Masern-Mumps-Röteln, *MMRV* Masern-Mumps-Röteln-Varizellen

### 6.2 Impfschemata für inaktivierte Impfstoffe – nach Indikation

Bei fehlender Grundimmunisierung sollte diese ehestmöglich nachgeholt werden; bei dokumentierter Grundimmunisierung sollten Auffrischungen zeitgerecht erfolgen. Alle durchgeführten Impfungen und ggf. zuletzt im Impfpass vermerkte, noch gültige Impfungen sollen in den **elektronischen Impfpass** eingetragen werden.

Aus immunologischer Sicht sind Kombinationen inaktivierter Impfstoffe prinzipiell immer möglich. Dabei ist auf die Reaktogenität der Impfstoffe, den Gesundheitszustand der Betroffenen sowie auf den geplanten Therapiebeginn Rücksicht zu nehmen. Es empfiehlt sich bei jedem Besuch eine individuelle Reevaluierung des erstellten Impfplans.

#### 6.2.1 Impfschema A – Routineimpfungen

**Abb. 9:**
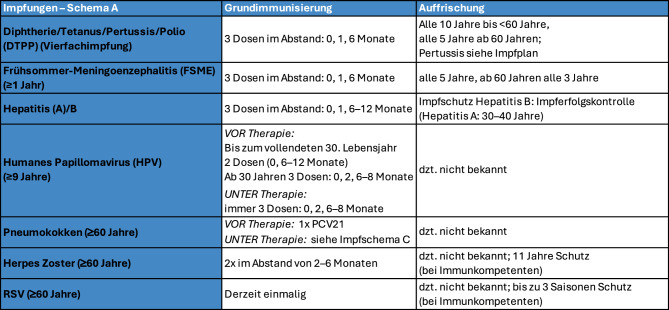
Impfschema A – Routineimpfungen laut österreichischem Impfplan (Erwachsene ≥ 18 Jahren; [6]). *PCV* Pneumokokken-Konjugat-Impfstoff, *RSV* humanes respiratorisches Synzytialvirus

Vor jeder immunsuppressiven Therapie sollten die in Abb. 9 aufgeführten Impfungen (als Impfschema A bezeichnet) durchgeführt werden (*altersentsprechend* nach dem österreichischen Impfplan [6]). Sie werden in den folgenden Abbildungen als Impfschema A bezeichnet.

**Wichtig**: Abb. 9 bezieht sich auf Routineimpfungen für Erwachsene ≥ 18 Jahre. Für das entsprechende Kinderimpfprogramm wird auf die Übersichtstabelle im österreichischen Impfplan [6] verwiesen (http://www.sozialministerium.gv.at/impfplan) – siehe auch Abschnitt 9.

#### 6.2.2 Impfschema B – saisonale Impfungen

**Abb. 10:**
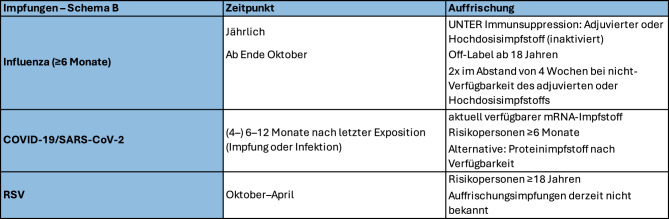
Impfschema B – saisonale Impfungen. (Quelle: österreichischer Impfplan [6].) *COVID-19* „Coronavirus disease 2019“, *mRNA* Messenger-Ribonukleinsäure, *RSV* humanes respiratorisches Synzytialvirus, *SARS-CoV‑2* „severe acute respiratory syndrome Coronavirus 2“

Saisonale Impfungen werden auch unter immunsupprimierender Therapie empfohlen (*altersentsprechend*) und werden in den folgenden Abbildungen als Impfschema B bezeichnet (Abb. 10).

#### 6.2.3 Impfschema C – ergänzende Impfungen vor oder unter Therapie mit Immunsuppressiva Grad III

**Abb. 11:**
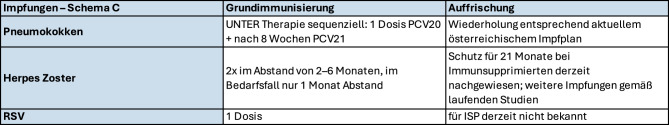
Impfschema C – indizierte Impfungen ab dem Alter von ≥ 18 Jahren. (Quelle: österreichischer Impfplan [6].) *ISP* Immunsuppression, *PCV* Pneumokokken-Konjugat-Impfstoff, *RSV* humanes respiratorisches Synzytialvirus

Die in Abb. 11 dargestellten Impfungen werden *ab 18 Jahren* bei jeglicher Form der Immunsuppression empfohlen und werden in den folgenden Abbildungen als Impfschema C bezeichnet).

**Hinweis**: Es kann durch die Grundkrankheit per se, auch ohne stark immunsuppressive Therapie, die Impfung nach Impfschema C indiziert/sinnvoll sein, siehe spezielle Indikationen laut jeweils aktuellem österreichischem Impfplan (siehe Abschnitt 9).

#### 6.2.4 Impfschema D – Impfungen gegen bekapselte Bakterien

**Abb. 12:**
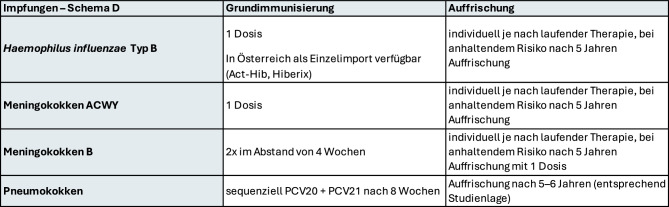
Impfschema D – indizierte Impfungen gegen bekapselte Bakterien (alle Altersgruppen). (Quelle: österreichischer Impfplan [6].) *PCV* Pneumokokken-Konjugat-Impfstoff

Bei geplanter B‑Zell-depletierender Therapie, anatomischer oder funktioneller Asplenie, Komplementdefekten (MBL-Mangel) oder Komplementinhibitoren (Anti-C5-Antikörper etc.) sollten die in Abb. 12 aufgeführten Impfungen ergänzend zu den Impfungen der Gruppen A bis C durchgeführt werden. Diese werden in den folgenden Abbildungen als Impfschema D bezeichnet).

Die unter dem Abschnitt 6.2 dargestellten Impfschemata werden im folgenden Abschnitt 6.3 je nach Indikation als Impfschema A, B, C oder D gelistet.

### 6.3 Anwendung der Impfschemata nach Indikation

#### 6.3.1 Angeborene/primäre Immundefekte

Bei angeborenen Immundefekten dürfen inaktivierte Impfstoffe verabreicht werden, jedoch muss je nach betroffenem Teil des Immunsystems unter Umständen mit suboptimalem Impfansprechen gerechnet werden, weswegen Impferfolgskontrollen empfohlen werden. Für die Einteilung der primären Immundefekte siehe Tab. 17. *Für das Routineimpfprogramm für Kinder wird auf die Übersichtstabelle im österreichischen Impfplan *[6]* verwiesen – siehe auch Abschnitt 9.* Weitere Hinweise finden sich in der Literatur [83, 150–155], wobei besonders auf das *Australian Immunization Handbook* [83] verwiesen werden soll.

**Abb. 13:**
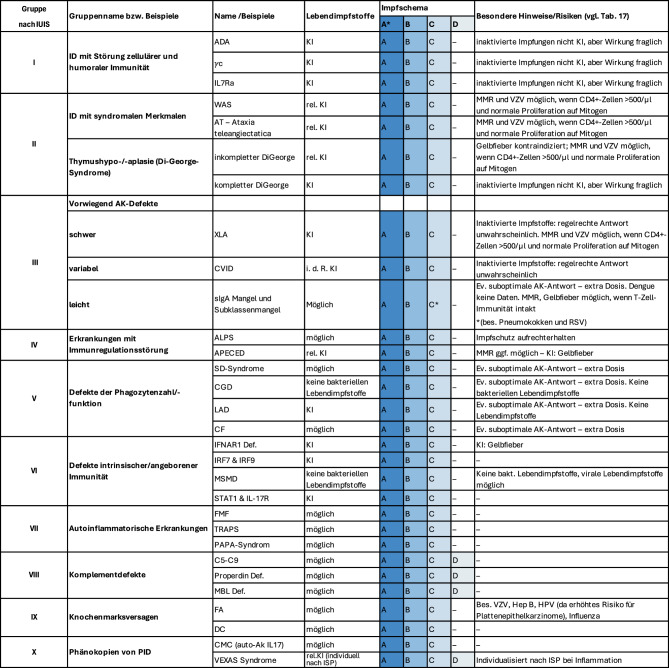
Impfen bei angeborenen/primären Immundefekten bei Erwachsenen ≥ 18 Jahren [83, 150–155]. Für PID-bezogene Abkürzungen siehe Tab. 17. *AHI* Australian Handbook of Immunization, *AK* Antikörper, *CDC* Centers for Disease Control and Prevention, *Def*. Defizienz, *ev* eventuell, *Hep B* Hepatitis B, *HPV* Humane Papillomaviren, *ID* Immundefizienz, *IFN* Interferon, *ISP* Immunsuppression, *KI* Kontraindikation, *KM* Knochenmark, *MMR* Masern-Mumps-Röteln, *MMRV* Masern-Mumps-Röteln-Varizellen, *OPV* orale Poliovakzine, *PID* primäre Immundefizienz, *rel.* relativ, *RSV* Humanes respiratorisches Synzytialvirus, *V* Varizellen, *VZV* Varicella-Zoster-Virus. * WICHTIG: Abb. 13 bezieht sich auf Impfungen für Erwachsene ≥18 Jahre. Für das entsprechende Kinderimpfprogramm wird auf die Übersichtstabelle im österreichischen Impfplan verwiesen (http://www.sozialministerium.gv.at/impfplan) – siehe auch Abschnitt 9 [6].

Das Ergebnis des österreichischen Neugeborenenscreenings zur Erkennung schwerer (kombinierter) Immundefekte ist zu berücksichtigen: Es können kombinierte Immundefekte, B‑Zell-Defekte sowie T‑Zell-Defekte diagnostiziert werden. Dies ist in Bezug auf Kontraindikationen für Lebendimpfungen zu berücksichtigen (Abb. 13).

#### 6.3.2 Konventionelle Therapeutika/Immunsuppressiva (Grad I–III)

Dieser Abschnitt bezieht sich auf die Impfungen, die unter konventioneller Immuntherapie, beispielsweise mit Methotrexat, Kortison, allen Nichtinterferonen und monoklonalen Antikörpern, gegeben werden sollen/können. Hier sei noch einmal darauf hingewiesen, dass Lebendimpfungen nur nach Einhaltung eines bestimmten Abstands gegeben werden können – siehe die Kommentare bei den jeweiligen Impfschemata.

##### 6.3.2.1 Glukokortikoide

**Abb. 14:**
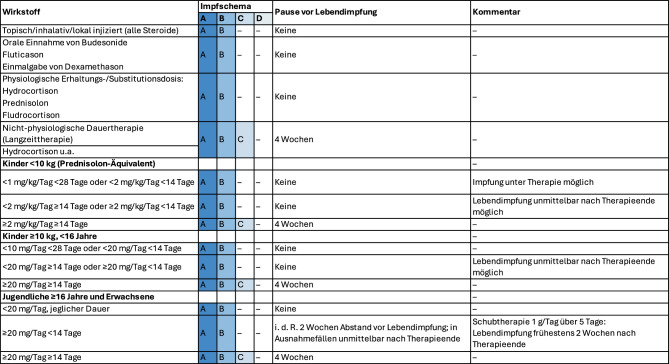
Impfen bei Gabe von Glukokortikoiden [8, 83]. *i.* *d.* *R.* in der Regel. *Asterisk* Cave: Altersempfehlung bei Impfschema einhalten – siehe österreichischer Impfplan [6]

Bei Glukokortikoiden sind die Abstände vor und nach Verabreichung eines Lebendimpfstoffs abhängig von der Dosis und der Behandlungsdauer (Abb. 14):

- Bei Kurzzeittherapie für < 2 Wochen, bei einer niedrigen Dosis (Prednisolon < 20 mg/d oder entsprechende Äquivalenzdosis) oder bei einer längerfristigen alternierenden Tagestherapie (verabreicht jeden 2. Tag) sowie bei topischer, inhalativer, intraartikulärer oder intrabursaler Applikation ist kein Abstand vor und nach der Impfung nötig.
- Bei Prednisolon-Gabe > 20 mg/d oder entsprechende Äquivalenzdosis aber < 2 Wochen sollten Lebendimpfungen erst 2 Wochen nach Therapieende gegeben werden.
- Bei Schubtherapie von 1 g/d über 5 Tage sollte zumindest 2 Wochen Abstand vor Lebendimpfungen eingehalten werden.
- Bei Konzentrationen > 20 mg/d für > 2 Wochen sollten Lebendimpfungen erst 4 Wochen nach Therapieende gegeben werden.
- Bei Verabreichung von Depotkortison: vor Impfung 2 Wochen, nach Impfung 2 bis 4 Wochen Abstand.
- Prednisolon > 20 mg/d oder entsprechende Äquivalenzdosis für > 2 Wochen: vor und nach Impfung je 4 Wochen Abstand.

##### 6.3.2.2 Methotrexat und Azathioprin

**Abb. 15:**
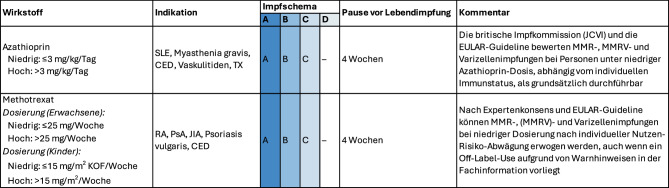
Impfen bei Gabe von Methotrexat und Azathioprin [8, 83]. *CED* chronisch-entzündliche Darmerkrankungen, *EULAR* European Alliance of Associations for Rheumatology, *JCVI* Joint Committee on Vaccination and Immunisation, *JIA* juvenile idiopathische Arthritis, *KOF* Körperoberfläche, *MMR* Masern-Mumps-Röteln, *MMRV* Masern-Mumps-Röteln-Varizellen, *PsA* Psoriasis-Arthritis, *RA* rheumatoide Arthritis, *SLE* systemischer Lupus erythematodes, *TX* Transplantation

Bei Personen, die Methotrexat ≤ 0,4 mg/kg/Woche (oder ≤ 25 mg/Woche bei Fixdosis) oder Azathioprin ≤ 3 mg/kg/Tag erhalten, können die Abstände zur Impfung bei Bedarf verkürzt werden. Um das Ansprechen der Influenzaimpfung zu verbessern, wurde eine Pausierung der Methotrexatgabe von 2 Wochen nach der Impfung empfohlen [8, 156]. Diese Vorgehensweise sollte aber mit dem behandelnden Arzt besprochen werden (Abb. 15).

##### 6.3.2.3 Weitere konventionelle Immunsuppressiva

**Abb. 16:**
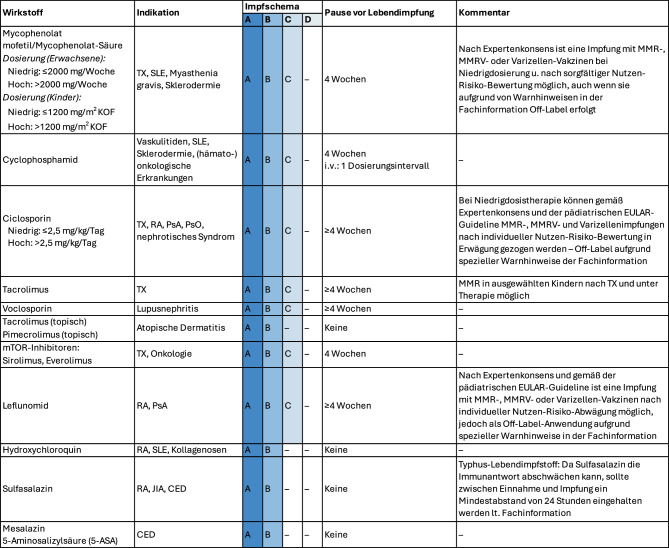
Impfen bei Gabe anderer konventioneller Therapeutika bzw. Immunsuppressiva. (Quellen: jeweilige Fachinformationen und [8, 157].) *CED* chronisch-entzündliche Darmerkrankungen, *EULAR* European Alliance of Associations for Rheumatology, *i.v.* intravenös, *JIA* juvenile idiopathische Arthritis, *KOF* Körperoberfläche, *MMR* Masern-Mumps-Röteln, *MMRV* Masern-Mumps-Röteln-Varizellen, *PsA* Psoriasis-Arthritis, *PsO* Psoriasis, *RA* rheumatoide Arthritis, *SLE* systemischer Lupus erythematodes, *TX* Transplantation

Weitere konventionelle Immunsuppressiva sind in Abb. 16 dargestellt.

##### 6.3.2.4 TNF-α- und Interleukininhibitoren

In Anlehnung an die aktuelle Empfehlung des American Collage of Rheumatology [8] ist bei allen Interleukin- und TNF-α-Inhibitoren, ein Abstand von **1 Dosierungsintervall **vor Applikation einer Lebendimpfung einzuhalten.

##### 6.3.2.4.1 TNF-α-Inhibitoren (Grad IIIa)

**Abb. 17:**
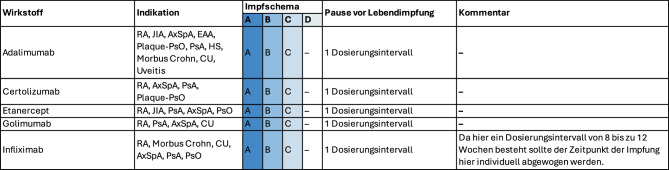
Impfen bei Gabe von TNF-α-Inhibitoren. (Quellen: jeweilige Fachinformationen und [8, 83].) *AxSpA* axiale Spondylarthritis, *CED* chronisch-entzündliche Darmerkrankungen, *CU* Cholitis ulcerosa, *EAA* enthesitisassoziierte Arthritis, *HPV* humanes Papillomavirus, *HS* Hidradenitis suppurativa, *JIA* juvenile idiopathische Arthritis, *PsA* Psoriasis-Arthritis, *PsO* Psoriasis, *RA* rheumatoide Arthritis

Unter TNF-α-Therapie kommt es bei nahezu allen inaktivierten Impfstoffen zu einer verminderten Immunantwort oder zu einem frühzeitigen Abfall der Immunantwort, besonders bei Primärimmunisierung [43], wenngleich zwei randomisierte, kontrollierte Studien keinen negativen Effekt der Impfantwort auf Influenzavakzinierung während einer TNF-α-Inhibitortherapie fanden [158, 159]. Dennoch sollte eine Therapieindikation nicht verzögert werden und eine Durchimmunisierung kann parallel zu Therapiebeginn erfolgen (Abb. 17).

##### 6.3.2.4.2 Interleukininhibitoren (Grad II–IIIa)

**Abb. 18:**
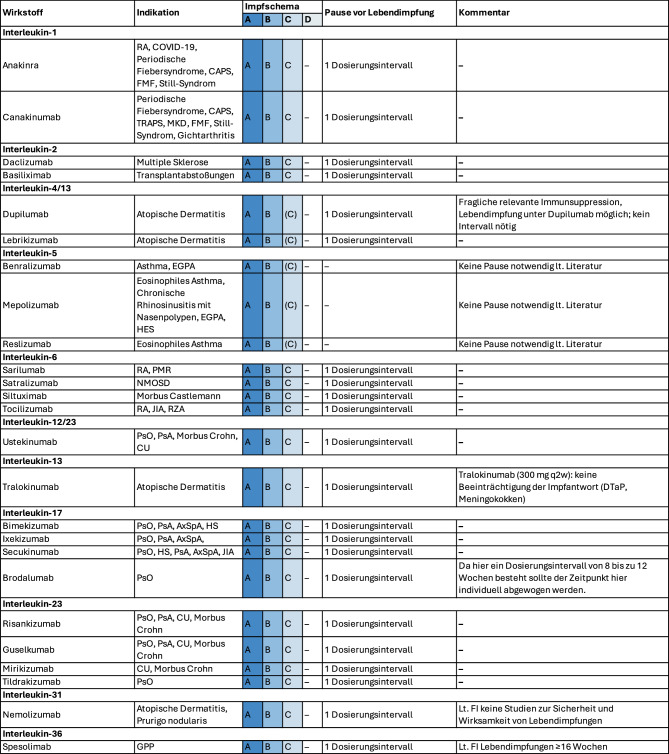
Impfen bei Gabe von Interleukininhibitoren. (Quellen: jeweilige Fachinformationen und [8, 83, 160–162].) *AxSpA* axiale Spondylarthritis, *CAPS* cryopyrinassoziierte periodische Syndrome, *CED* chronisch-entzündliche Darmerkrankungen, *COVID-19* „Coronavirus disease 2019“, *CU* Colitis ulcerosa, *DTaP* Diphtherie‑, Tetanus- und azellulärer Pertussis-Impfstoff, *EGPA* eosinophile Granulomatose mit Polyangiitis, *FI* Fachinformation, *FMF* familiäres Mittelmeerfieber, *GPP* generalisierte pustulöse Psoriasis, *HES* Hypereosinophiliesyndrom, *HS* Hidradenitis suppurativa, *JIA* juvenile idiopathische Arthritis, *MKD* Mevalonatkinasedefizienz, *NMOSD* Neuromyelitis-optica-Spektrum-Erkrankungen, *PMR* Polymyalgia rheumatica, *PsA* Psoriasis-Arthritis, *PsO* Psoriasis, *q2w* Einmalgabe alle 2 Wochen, *RA* rheumatoide Arthritis, *RZA* Riesenzellarteriitis, *TRAPS* Tumornekrosefaktorrezeptor-assoziierte periodische Fiebersyndrome

Interleukininhibitoren beinhalten umfassende Indikationen und Halbwertszeiten (Abb. 18).

#### 6.3.3 JAK-Inhibitoren (Grad II–III)

**Abb. 19:**
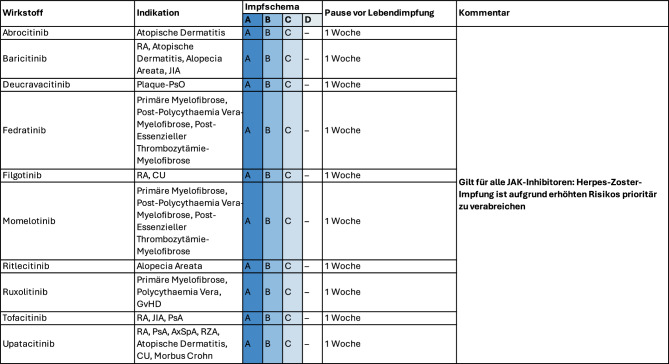
Impfen bei Gabe derzeit gängiger JAK-Inhibitoren. (Quellen: jeweilige Fachinformationen und [83].) *AxSpA* axiale Spondylarthritis, *CU* Colitis ulcerosa, *GvHD* Graft-versus-Host-Erkrankung, *JAK* Januskinase, *JIA* juvenile idiopathische Arthritis, *PsA* Psoriasis-Arthritis, *PsO* Psoriasis, *RA* rheumatoide Arthritis, *RZA* Riesenzellarteriitis

Zum Impfansprechen unter JAK-Inhibitoren gibt es deutlich weniger Daten als unter TNF-α-Inhibitor-Therapie. Da die Halbwertszeit von JAK-Inhibitoren extrem kurz ist (3–6 h), ist zwischen der letzten JAK-Inhibitorgabe und einer Lebendimpfung ein Intervall von 1 Woche ausreichend. Danach müssen aber (maximal) 4 Wochen (in Abhängigkeit vom Lebendimpfstoff und der Dringlichkeit des Therapiewiederbeginns) vor der erneuten JAK-Inhibitoreinnahme abgewartet werden (Abb. 19).

#### 6.3.4 Komplementinhibitoren (Grad II–IIIa)

**Abb. 20:**
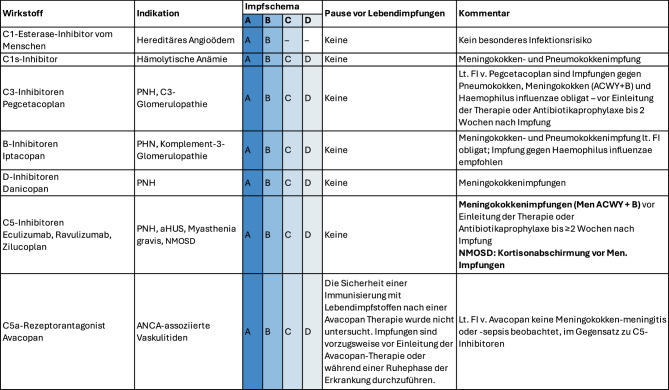
Impfen bei Gabe von Komplementinhibitoren. (Quellen: jeweilige Fachinformationen und [83, 163].) *aHUS* atypisches hämolytisch-urämisches Syndrom, *ANCA* anti-Neutrophile zytoplasmatische Antikörper, *FI* Fachinformation, *NMOSD* Neuromyelitis-optica-Spektrum-Erkrankungen, *PNH* paroxysmale nächtliche Hämoglobinurie

Komplementinhibitoren sind mit einem erhöhten Risiko für invasive Infektionen durch bekapselte Bakterien, insbesondere Meningokokken, assoziiert. Daher sollen Impfungen gegen Meningokokken (ACWY und B), Pneumokokken sowie *Haemophilus influenzae* Typ B möglichst vor Therapiebeginn erfolgen. Bei vorzeitigem Therapiebeginn wird eine antibiotische Prophylaxe bis mindestens 2 Wochen nach erfolgter Meningokokkenimpfung empfohlen. Lebendimpfstoffe sind unter Komplementinhibitortherapie in der Regel nicht kontraindiziert. Komplementinhibitoren sind Abb. 20 zu entnehmen.

#### 6.3.5 T-Zell-Inhibitoren (Grad IIIa)

**Abb. 21:**
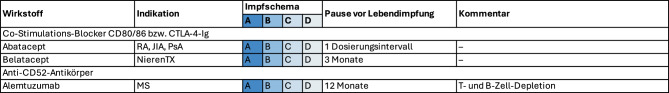
Impfen bei Gabe von T‑Zell-Inhibitoren. (Quellen: jeweilige Fachinformationen und [83].) *CTLA-4* „cytotoxic T-lymphocyte-associated protein 4“, *JIA* juvenile idiopathische Arthritis, *MS* Multiple Sklerose, *PsA* Psoriasis-Arthritis, *RA* rheumatoide Arthritis, *TX* Transplantation

T‑Zell-Inhibitoren sind in Abb. 21 zusammengefasst.

#### 6.3.6 B-Zell-Inhibitoren/Depletoren (Grad IIIb)

**Abb. 22:**
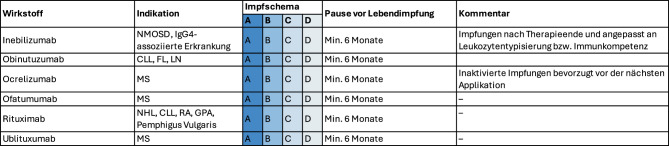
Impfen vor/bei Gabe von B‑Zell-Inhibitoren/Depletoren. (Quellen: jeweilige Fachinformationen und [83].) Anmerkung: Humorale Antworten sind während laufender Therapie nur eingeschränkt zu erwarten, es können aber positive Effekte durch zelluläre Impfantworten erreicht werden. Daher sind Impfungen gegen saisonale respiratorische Erkrankungen, wie Influenza oder COVID-19, unter Therapie dennoch sinnvoll. *CLL* chronisch-lymphatische Leukämie, *COVID-19* „Coronavirus disease 2019“, *FL* follikuläres Lymphom, *GPA* Granulomatose mit Polyangiitis, *IgG4* Immunglobulin G4, *LN* Lupus-Nephritis, *MS* Multiple Sklerose, *NHL* Non-Hodgkin Lymphom, *NMOSD* Neuromyelitis-optica-Spektrum-Erkrankungen, *RA* rheumatoide Arthritis

Unter der Anti-CD19/20-Antikörpertherapie kommt es zu einer deutlichen Immunsuppression, unter der eine Antikörperentwicklung auf Impfungen kaum zu erwarten ist. Die Impfversorgung sollte daher, wenn möglich, unbedingt *vor* der Therapie durchgeführt werden (Abb. 22).

Für Plasmazelldepletoren siehe Abb. 26 und Abb. 27.

#### 6.3.7 Checkpoint-Inhibitoren

Beispiele für Checkpoint-Inhibitoren sind PD-1-, PD-L1- und CTLA-4-Inhibitoren. Die Datenlage zum immunsuppressiven Potenzial von Checkpoint-Inhibitoren ist gering, es ist jedoch aufgrund ihres Wirkmechanismus nicht mit einer eingeschränkten Immunantwort zu rechnen. Die Immunantwort auf Impfungen kann aber aufgrund der Grunderkrankung oder weiterer Therapien eingeschränkt sein. Bezogen auf die Sicherheit von Impfungen unter Checkpoint-Inhibitor-Therapie zeigte eine Übersichtsarbeit kein erhöhtes Risiko für immunmediierte Nebenwirkungen nach Influenza- und COVID-19-Impfungen [16]. Besonders bei Influenza- und COVID-19-Vakzinen wurde gezeigt, dass die Behandlung mit Checkpoint-Inhibitoren kein erhöhtes Nebenwirkungsrisiko hat und es sogar zu einem verbesserten Ansprechen auf die Tumorbehandlung führen kann. Dies wurde jüngst durch eine vermehrte Sensibilisierung der Tumoren auf Checkpoint-Inhibitoren bei Gabe von mRNA-Vakzinen erklärt [14].

**Abb. 23:**
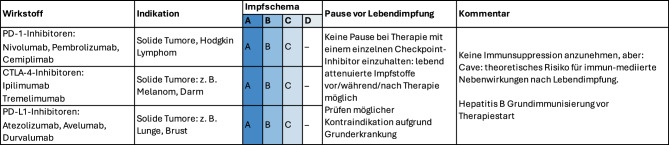
Impfen bei Gabe von Checkpoint-Inhibitoren. (Quellen: jeweilige Fachinformationen und [83].) *CTLA‑4* „cytotoxic T‑lymphocyte-associated protein 4“, *PD‑1* „programmed cell death protein 1“, *PD-L1* „programmed cell death-ligand 1“

Die Anwendung inaktivierter Impfstoffe ist jederzeit möglich. Für Lebendimpfstoffe gilt: Bei Therapie mit einem einzelnen Checkpoint-Inhibitor sind Impfungen mit Lebendimpfstoffen jederzeit möglich. Aufgrund der geringen Datenlage empfiehlt sich derzeit die Applikation von Lebendimpfungen eher nach Ende des Behandlungszyklus. Es gibt ein theoretisches Risiko für immunmediierte Nebenwirkungen wegen erhöhter T‑Zellaktivierung. Kontraindikationen gegen Lebendimpfungen können sich aber aufgrund der Grundkrankheit, z. B. hämatoonkologische Erkrankungen, oder zusätzlicher immunsuppressiver Therapien ergeben (Abb. 23).

#### 6.3.8 Zielgerichtete Therapien in der Onkologie/Hämatoonkologie

**Abb. 24:**
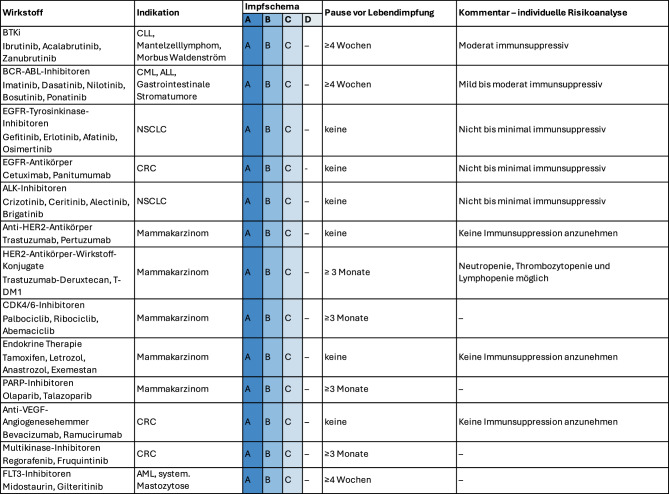
Impfen bei Gabe zielgerichteter Therapien in der Onkologie/Hämatoonkologie. (Quellen: jeweilige Fachinformationen und [83, 164, 165].) *ALK* „anaplastic lymphoma kinase“, *ALL* akute lymphatische Leukämie, *AML* akute myeloische Leukämie, *BCR-ABL* Breakpoint-cluster-region-Gen-Abelson-murine-leukemia-viral-oncogene-homolog-1-Gen, *BTKi* Bruton-Tyrosinkinase-Inhibitor, *CDK4/6* cyclinabhängige Kinasen 4 und 6, *CLL* chronisch-lymphatische Leukämie, *CML* chronisch myeloische Leukämie, *CRC* Kolorektalkarzinom, *EGFR* „epidermal growth factor receptor“, *FLT3* „Fms-related receptor tyrosine kinase 3“, *HER2* „human epidermal growth factor receptor 2“, *NSCLC* nichtkleinzelliges Lungenkarzinom, *PARP* „poly(ADP-ribose) polymerase“, *T‑DM1* Trastuzumab-Emtansin, *VEGF* „vascular endothelial growth factor“

Bei zielgerichteten Therapien ist eine individuelle Nutzen-Risiko-Analyse durchzuführen. Einige zielgerichtete Therapien führen zu Neutropenien und Immunsuppression. Impfungen sollten daher zwischen den Zyklen durchgeführt werden, wenn die Anzahl der Leukozyten und Neutrophilen im Normbereich ist (Abb. 24).

##### 6.3.8.1 Bispezifische Antikörper (BsAbs) und bispezifische T-Zell-Engager (BiTEs)

**Abb. 25:**
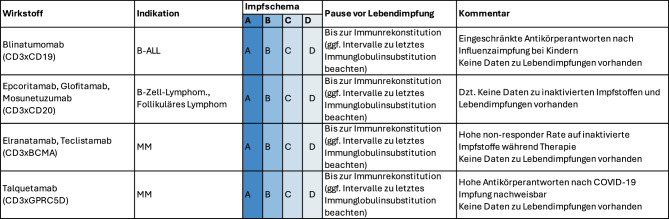
Bispezifische Antikörper und bispezifische T‑Zell-Engager. (Quellen: jeweilige Fachinformationen und [97, 166–171].) *ALL* akute lymphatische Leukämie, *COVID-19* „Coronavirus disease 2019“, *dzt.* derzeit, *ggf.* gegebenenfalls, *MM* Multiples Myelom

Es wird empfohlen, alle fälligen Impfungen vor der Einleitung bispezifischer Antikörper‑/BiTE-Therapien zu verabreichen, da ein besseres Impfansprechen vor Therapie als während der Therapie erwartet wird ([166–168]; Abb. 25).

##### 6.3.8.2 Antikörper-Wirkstoff-Konjugate (ADC)

**Abb. 26:**
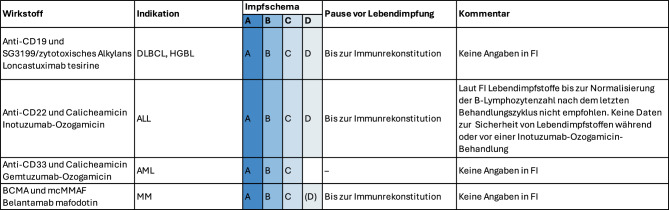
Beispielgebende Antikörper-Wirkstoff-Konjugate (*ADC*). (Quellen: jeweilige Fachinformationen.) *ALL* akute lymphatische Leukämie, *AML* akute myeloische Leukämie, *BCMA* B-Zell-Reifungsantigen, *DLBCL* diffus-großzelliges B‑Zell-Lymphom, *FI* Fachinformation, *HGBL* hochmalignes B‑Zell-Lymphom, *mcMMAF* Maleimidocaproyl-Monomethylauristatin F, *MM* Multiples Myelom

Bei Gabe von Antikörper-Wirkstoff-Konjugaten sind Immunsuppression und Infektionsrisiko stark abhängig von den jeweiligen Bestandteilen der Wirkstoffe. Es empfiehlt sich die Immunrekonstitution zu überprüfen und zwischen den Zyklen bei normaler Anzahl an Leukozyten bzw. neutrophilen Leukozyten zu impfen. Im Einzelfall können Patienten nach entsprechender Nutzen-Risiko-Abwägung individuell geimpft werden. Beispiele für Antikörper-Wirkstoff-Konjugate zeigt Abb. 26.

#### 6.3.9 Ergänzende immunsuppressive Medikamente mit ISP-Grad III

**Abb. 27:**
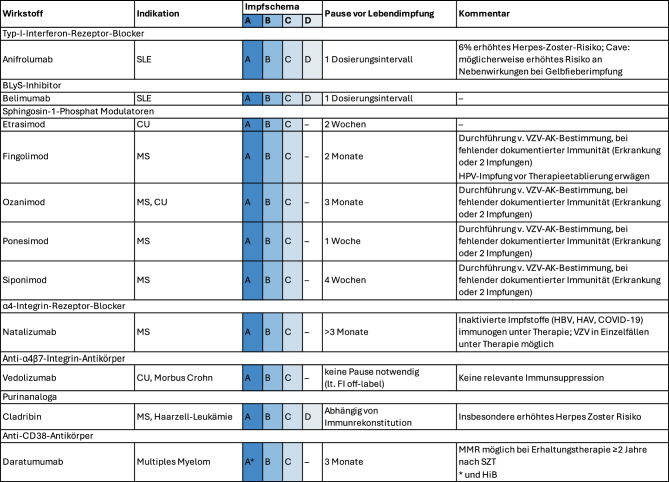
Ergänzung: Impfen bei Gabe von Immunsuppressiva mit ISP-Grad III. (Quellen: jeweilige Fachinformationen und [114, 172–174].) *AK* Antikörper, *BLyS* B-Lymphozyten-Stimulator, *COVID-19* „Coronavirus disease 2019“, *CU* Colitis ulcerosa, *FI* Fachinformation, *HAV* Hepatitis-A-Virus, *HBV* Hepatitis-B-Virus, *HiB* *Haemophilus influenzae* *B*, *HPV* humanes Papillomavirus, *MS* Multiple Sklerose, *SLE* systemischer Lupus erythematodes, *SZT* Stammzelltransplantation, *VZV* Varicella-Zoster-Virus

In diesem Abschnitt werden alle immunsuppressiven Medikamente mit ISP-Grad III zusammengefasst, die keiner der oben genannten Kategorien zuzuordnen sind (Abb. 27).

Bei Behandlung mit konventionellen Chemotherapeutika (Folsäureantagonisten, Purin- und Pyrimidinanaloga, Platinzytostatika, Taxane, Nitroharnstoffderivate, Stickstoff-Lost-Derivate, Alkylsulfonate, Triazene) muss vor Applikation einer Lebendimpfung ein Abstand von 6 Monaten nach Abschluss der Chemotherapie eingehalten werden. Im Einzelfall können Patienten nach entsprechender Nutzen-Risiko-Abwägung individuell geimpft werden. Bei Personen mit Lungenkarzinomen unter Therapie kann laut österreichischem Impfplan zusätzlich die *Haemophilus-influenzae*-Impfung sinnvoll sein [6].

#### 6.3.10 Immunmodulatoren mit ISP-Grad 0–II

**Abb. 28:**
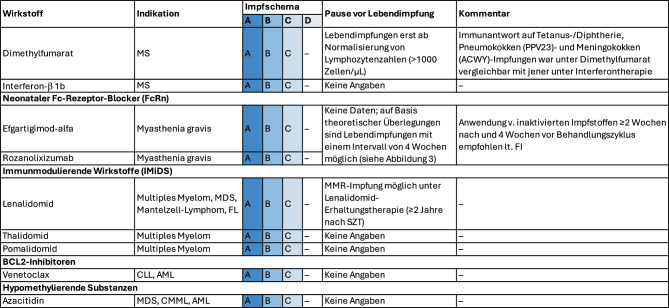
Ergänzung: Impfen bei Gabe von Immunmodulatoren mit ISP-Grad II. (Quellen: jeweilige Fachinformationen und [137, 175–177].) *AML* akute myeloische Leukämie, *BCL2* „B cell lymphoma 2“, *BRAF* „B-Raf proto-oncogene“, *CLL* chronisch-lymphatische Leukämie, *CMML* chronische myelomonozytäre Leukämie, *FI* Fachinformation, *FL* follikuläres Lymphom, *MDS* Myelodysplastisches Syndrom, *MMR* Masern-Mumps-Röteln, *MS* Multiple Sklerose, *PPV* Pneumokokken-Polysaccharid-Impfstoff, *SZT* Stammzelltransplantation

Folgende Substanzen haben per se keine immunsuppressive Wirkung. Lebendimpfungen können daher vor, während und nach Therapie gegeben werden. Achtung: Eine Beurteilung der Immunsuppression muss aber auch immer die Grunderkrankung der Betroffenen mit einbeziehen. Zum Beispiel: Azacitidin hat primär kaum eine immunsuppressive Wirkung, wird aber bei Personen mit hämatoonkologischen Erkrankungen appliziert, bei denen die Anwendung von Lebendimpfungen aufgrund der Erkrankung prinzipiell kontraindiziert ist. Die Impffähigkeit dieser Personen kann daher immer nur im Zusammenspiel zwischen der Grunderkrankung und der angewandten Medikamentengruppe beurteilt werden (Abb. 28 und 29).

**Abb. 29:**
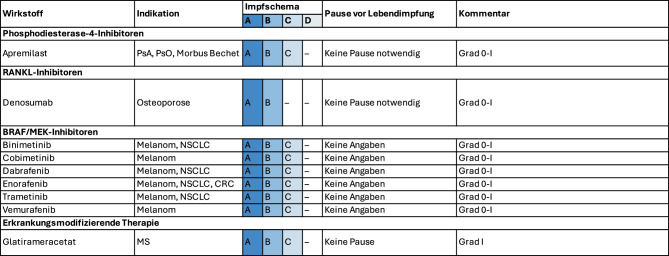
Ergänzung: Impfen bei Gabe von Immunmodulatoren mit ISP-Grad I. (Quellen: jeweilige Fachinformationen.) *BRAF* „B-Raf proto-oncogene“, *CRC* Kolorektalkarzinom, *MEK* „mitogen-activated protein kinase“, *MS* Multiple Sklerose, *NSCLC* nichtkleinzelliges Lungenkarzinom, *PsA* Psoriasis-Arthritis, *PsO* Psoriasis, *RANKL* „receptor activator of nuclear factor κB ligand“

#### 6.3.11 Nach Stammzelltransplantation (autolog, allogen)

Nach allogener Stammzelltransplantation dürfen frühesten nach 6 Monaten inaktivierte und nach 24 Monaten Lebendimpfungen appliziert werden. Aufgrund der Saisonalität und epidemiologischen Lage können die Influenza- und COVID-19-Impfungen auch bereits ab 3 Monate nach allogener Stammzelltransplantation verabreicht werden. Nach autologer Stammzelltransplantation kann bereits nach 3 Monaten mit gewissen inaktivierten Impfungen begonnen werden und eventuell können bereits nach 12 Monaten (wenn keine immunsuppressiven Therapien gegeben werden) Lebendimpfungen appliziert werden [116, 148]. Weiters besteht, besonders bei hohem Infektionsrisiko, nach individueller Nutzen-Risiko-Analyse die Möglichkeit, ab 12 Monaten nach allogener Stammzelltransplantation Lebendimpfungen zu verabreichen [116, 148]. Das ist vor allem für MMR-Impfungen in Ausbruchsituationen relevant [178]. Zudem gibt es erste Daten zur Sicherheit und guten Verträglichkeit der MMR-Impfung 24 Monate nach autologer Stammzelltransplantation unter laufender Therapie mit Lenalidomid (Immunmodulator) oder Bortezomib (Proteasominhibitor; [177]). Ab 24 Monaten nach Stammzelltransplantation (in Krankheitsremission, ohne akuter GvHD und ohne immunsuppressive Therapien) kann eine Grundimmunisierung wie bei Immungesunden altersentsprechend durchgeführt werden – es ist nicht mehr nötig, auf das intensivierte Grundimmunisierungsschema zurückzugreifen (Tab. 21).

**Tab. 21:** Impfen nach Stammzelltransplantation. (Adaptiert nach [83, 178, 179])

**Frühester Zeitpunkt nach SZT** **autolog/allogen**		**Impfstoff**	**Grundimmunisierung**	**Auffrischungen**	**Kommentar**
≥ 3 Monate		Influenza	1 × saisonal mit adjuviertem oder Hochdosisimpfstoff (ggf. mit Normaldosis 2 × mit 4 Wochen Abstand)	Jährlich	Siehe ergänzend aktuelle Empfehlung österr. Impfplan [6] COVID-19: manche Guidelines Grundimmunisierung mit 2 Dosen
≥ 3 Monate		COVID-19	3 × je 1 Monate Abstand (0, 1, 2 Monate)	Nach 6 Monaten (danach altersentsprechend je nach Indikation)	
Autolog: ≥ 3 Monate	Allogen: ≥ 3–6 Monate	Pneumokokken	3 × PCV20: je 1 Monat Abstand (0, 1, 2 Monate)	1 × PCV21 nach 12 Monaten	–
Autolog: ≥ 3–6 Monate	Allogen: ≥ 6–12 Monate	RSV	1 ×	Dzt. nicht bekannt	Adjuvierter oder nichtadjuvierter Impfstoff verfügbar; dzt. keine Daten zu Abstand nach SZT; Serokonversion nach RSV-Impfung erst nach 12 Monaten Post-allo-SZT [179]
Autolog: ≥ 3–6 Monate*	Allogen: ≥ 6–12 Monate*	Herpes Zoster	2 × Abstand 2–6 Monate	Dzt. nicht bekannt	Ggf. späterer Impfzeitpunkt, wenn unter antiviraler Prophylaxe; * Nur eingeschränkt Daten nach allogener SZT
≥ 6 Mo		Diphtherie, Tetanus, Pertussis, Polio (IPV), Hepatitis B, HiB	3 × je 1 Monat Abstand (0, 1, 2 Monate)	Nach 12 Monaten (danach altersentsprechende Intervalle)	Bevorzugt erhöhter Diphtherie- und Tetanus-Antigengehalt; Off-label 6‑fach Kinderimpfstoff möglich; Hepatitis B Antikörperkontrolle ab 4 Wochen nach Auffrischung, da niedriger Antigengehalt in 6‑fach Impfstoff
≥ 6 Monate		FSME	3 × je 1 Monat Abstand (0, 1, 2 Monate)	Nach 12 Monaten (danach altersentsprechende Intervalle)	Impferfolgskontrolle nach Grundimmunisierung empfohlen
≥ 6 Monate		Meningokokken B	2 × Abstand ≥ 4 Wochen	–	Grundimmunisierung je nach Alter. Weitere Auffrischungen nur bei fortbestehender Indikation nach 5 Jahren
≥ 6 Monate		Meningokokken ACWY	2 × Abstand ≥ 4 Wochen	–	
≥ 6 Monate		Hepatitis A	2 × Abstand 6–12 Monate	–	Je nach Indikation, besonders bei Reisen in Endemiegebiete
≥ 6 Monate		HPV	3 × Abstand: (0, (1–) 2, 6–8 Monate)	Dzt. nicht bekannt	Altersunabhängig, ggf. nach Risikoabschätzung
≥ 24 Monate**		MMR und VZV (wenn seronegativ)	2 × Abstand ≥ 4 Wochen	–	** Wenn keine akute GvHD und keine laufende immunsupprimierende Therapie (≥ 12 Monate, siehe Text)

*COVID-19* „Coronavirus disease 2019“, *FSME* Frühsommer-Meningoenzephalitis, *dzt.* derzeit, *ggf.* gegebenenfalls, *GvHD* Graft-versus-host-Erkrankung, *HiB Haemophilus influenzae B, HPV* humanes Papillomavirus, *IPV* inaktivierter Polio-Impfstoff, *MMR* Masern-Mumps-Röteln-Dreifachimpfung, *PCV* Pneumokokken-Konjugat-Impfstoff, *RSV* humanes respiratorisches Synzytialvirus, *SZT* Stammzelltransplantation, *VZV* Varicella-Zoster-Virus

#### 6.3.12 Vor und nach CAR-T- und anderen Zell-basierten Immuntherapien

Vor Beginn einer CAR-basierten Therapie (d.h. CAR-T- oder andere Zell-basierte Therapie) wird derzeit eine Impfung gegen Influenza, COVID-19, Pneumokokken, Herpes Zoster und Hepatitis B empfohlen [121].

Für Impfungen nach CAR-basierter Therapie wird nach heutigem Wissenstand ein Impfschema wie nach allogener Stammzelltransplantation empfohlen bis weitere Daten verfügbar sind. Der Impfzeitpunkt vor allem bei Lebendimpfungen sollte an die Immunrekonstitution angepasst werden, wofür folgende Parameter herangezogen werden können: Gesamt IgG ≥ 400 mg/dl, CD19^+^-B-Zellen nachweisbar bzw. ≥ 20 Zellen/µl, CD4^+^-T-Zellen > 200/µl [120, 180].

Empfohlen wird, routinemäßig ein Lymphozytenprofiling (3–)6 Monate nach Therapie durchzuführen, um den Stand der Immunrekonstitution einschätzen und das Impfvorgehen danach ausrichten zu können. Die folgenden Empfehlungen sind aus Ermangelung an Studiendaten weitgehend an die allogene SZT angepasst (Tab. 22).

**Tab. 22:** Impfen von Erwachsenen vor und nach Behandlung mit CAR-T- bzw. Zell-basierter Therapie. (Modifiziert nach [122, 123])

**Impfstoff**	**Vor CAR‑T**	**Zeitpunkt nach CAR‑T**	**Grundimmunisierung**	**Auffrischungen**	**Kommentar**
Influenza	1 ×^a^	≥ (3^d^–)6 Monate	1 Dosis mit adjuviertem oder Hochdosisimpfstoff (ggf. Normaldosis 2 × mit 4 Wochen Abstand)	Saisonal jährlich	Saisonal
COVID-19	1 ×^a^	≥ (3^d^–)6 Monate	3 × je 1 Monat Abstand	Nach (4–)6 Monaten	Saisonal
RSV	1 ×^a^	(3^d^–)6 Monate	1 ×	Dzt. nicht bekannt	Impfung nach Saisonalität planen
Pneumokokken	^b^	≥ 6 Monate	3 × PCV20 je 1 Monat Abstand	Nach 12 Monaten PCV21	–
Herpes Zoster	^b,c^	≥ 6 Monate	2 × Abstand 2–6 Mo	Dzt. nicht bekannt	–
Diphtherie, Tetanus, Pertussis, Polio (IPV), Hepatitis B, HiB	^b^	≥ 6 Monate	3 × Abstand ≥ 1 Monat	Wie bei SZT	6‑fach Impfstoff mit erhöhtem Di/Tet-Antigengehalt möglich; Off-label 6‑fach Kinderimpfstoff nach CAR-T möglich
FSME	^b^	≥ 6 Monate	3 × Abstand ≥ 1 Monat	–	–
Meningokokken B	^b^	≥ 6 Monate	2 × Abstand ≥ 4 Wochen	–	Weitere Auffrischungen nur bei fortbestehender Indikation nach 5 Jahren
Meningokokken ACWY	^b^	≥ 6 Monate	2 × Abstand ≥ 4 Wochen	–	–
Hepatitis A	^b^	≥ 6 Monate	2 × 6 Monate Abstand	–	Weitere Impfungen individuell je nach Reiseanamnese
HPV	^b^	≥ 6 Monate	3 × Abstand: (0, (1–)2, 6–8 Monate)?	–	Altersentsprechend
MMR und VZV (wenn seronegativ)	^b^	≥ 12 Monate	2 × Abstand ≥ 4 Wochen	–	Nach Immunrekonstitution und wenn keine laufende Immunsuppression

*CAR* „chimeric antigen receptor“, *COVID-19* „Coronavirus disease 2019“, *Di/Tet* Diphtherie/Tetanus, *dzt.* derzeit, *FSME* Frühsommer-Meningoenzephalitis, *ggf.* gegebenenfalls, *HiB Haemophilus influenzae* Typ B, *HPV* humanes Papillomavirus, *IPV* inaktivierter Polio-Impfstoff, *MMR* Masern-Mumps-Röteln, *PCV* Pneumokokken-Konjugat-Impfstoff (PCV20 oder PCV21), *PPSV23* 23-valenter Pneumokokken-Polysaccharid-Impfstoff, *RSV* humanes respiratorisches Synzytialvirus, *SZT* Stammzelltransplantation, *VZV* Varicella-Zoster-Virus

^a^ Aufgrund der Saisonalität Impfung vor CAR-T-Zell-Therapie empfohlen

^b^ Je nach zeitlicher Planbarkeit und bei Indikation bis zu 2 Wochen bzw 4 Wochen (bei Lebendimpfstoffen) vor CAR-T-Zell-Therapie empfohlen, ansonsten nachher

^c^ Seropositive Personen; auch bei seronegativen Personen möglich [60]

^d^ Ab 3 Monaten, wenn während der Saison

#### 6.3.13 Impfungen bei solider Organtransplantation

Grundsätzlich sollen alle Impfungen, insbesondere Lebendimpfungen, vor SOT appliziert werden. Nach SOT sollen nur noch ausständige und saisonale Impfungen verabreicht oder bei Seronegativität geimpft werden (Tab. 23).

**Tab. 23:** Impfen vor und nach SOT. (Adaptiert nach [83, 125, 127, 181])

**Impfstoff**	**Vor SOT**		**Nach SOT**		**Kommentar**
	**Zeitpunkt**	**Schema**	**Zeitpunkt**	**Schema**	
**Inaktivierte Impfstoffe**					
*Impfungen gegen respiratorische Erkrankungen*					
Influenza	Bis zu 2 Wo vor TX (bevorzugt Okt/Nov)	1 Dosis	Frühestens 1 Mo, besser 3–6 Mo nach SOT (bevorzugt Okt/Nov)	1 Dosis (s. österr. Impfplan)	Hochdosis oder adjuvierter Impfstoff nach SOT: 2 Impfdosen im 1. Jahr nach SOT, danach 1 Dosis/Jahr [83]
COVID-19	1 Mo (bis spätestens 2 Wo) vor SOT	Grundimmunisierung und ≥ 2 Booster sollen vorhanden sein	3–6 Mo nach SOT	Alle 6–12 Monate 1 Dosis (bevorzugt Sep/Okt)	Bei Herz-TX und Lungen-TX: 2 Dosen im Abstand von 3 Mo [181]
RSV	Bis zu 2 Wo vor SOT	1 Dosis (idealerweise vor Saison: Okt–Apr)	3–6 Mo nach SOT	1 Dosis (Saison: Okt–Apr)	VE und weitere Impfungen > 6 Monate abhängig vom Schweregrad der Immunsuppression
Pneumokokken	Bis zu 2 Wo vor SOT	1 Dosis PCV oder bei bestehender Immunsuppression sequenziell PCV20 + PCV21	3–6 Mo nach SOT	Sequenziell PCV20, nach 8 Wo PCV21	Wiederholung nach 5–6 Jahren in Abhängigkeit von Studiendaten (Kinder: siehe österr. Impfplan)
HiB	Bis zu 2 Wo vor SOT	1 Dosis	3–6 Mo nach SOT	1 Dosis	Bes. bei Lungen-TX
*Standardimpfungen*					
DTPP	Bis zu 2 Wo vor SOT	1 Dosis	3–6 Mo nach SOT	1 Dosis	Impferfolgskontrolle
Hepatitis B	Bis zu 2 Wo vor SOT	0–1–6/12 Mo	3–6 Mo nach SOT	Min. 1 Dosis bevorzugt mit Hochdosis- oder adjuvierten Impfstoffen	Impferfolgskontrolle bzw. 0–1–6/12 bei Leber-TX, Nieren-TX
Hepatitis A	Bis zu 2 Wo vor SOT	0–6 Mo	3–6 Mo nach SOT	Min. 1 Dosis	Impferfolgskontrolle bes. bei Leber-TX
Meningokokken ACWY+B	Bis zu 2 Wo vor SOT	Wie allgemeine Bevölkerung	3–6 Mo nach SOT	Bei erhöhtem Risiko für invasive M.E.	Bei Nieren-TX vor GvHD-Behandlung mit Eculizumab vor SOT; bei spezifischen Indikationen: 1 Dosis vor und nach SOT (Men B 2 Dosen; [181])
HPV	Bis zu 2 Wo vor SOT	0–2–6 Mo	3–6 Mo nach SOT	Bei vollständiger GI, keine weitere Dosis nötig	Bei Personen > 30 Jahren: Risikoabschätzung, ob weitere Impfungen nötig
Herpes Zoster	Bis zu 2 Wo vor SOT	0–2/6 Mo	3–6 Mo nach SOT	Bei fehlender Impfung: 2 Dosen	Generell empfohlen; besonders bei Nieren-TX: 2 Dosen im Mindestabstand von 4–8 Wo [181]
*Reiseimpfungen*					
Tollwut	Bis zu 2 Wo vor SOT (Anlassfall)	3 Dosen	3–6 Monate	3 Dosen	Bei Exposition immer PEP und IG!!
Chikungunya (inakt.)	Bis zu 2 Wo vor SOT	1 Dosis	Keine Daten	Keine Daten	–
Typhus (inakt.)	Bis zu 2 Wo vor SOT	1 Dosis	3–6 Mo	Bei Indikation	VE unklar
Japanische Enzephalitis	Bis zu 2 Wo vor SOT	3 Dosen 0–1–12 Mo	Frühestens 3–6 Mo	Bei Indikation	VE unklar
**Lebendimpfstoffe (bis zu 4 Wo vor TX)**					
*Standardimpfungen*					
MMR	Spätestens 4 Wo vor SOT 2. MMR	2 Dosen im Mindestabstand von 4 Wo bei Seronegativität	Frühestes 12 Mo nach SOT nach Immunoprofiling	2 Dosen bei Seronegativität	IG-Substitution bei Kontakt von Seronegativen
Varizellen	Spätestens 4 Wo vor SOT 2. VZV	2 Dosen im Mindestabstand von 4 Wo bei Seronegativität	Frühestens 12 Mo nach SOT nach Immunoprofiling	2 Dosen bei Seronegativität	IG-Substitution bei Kontakt von Seronegativen
*Reiseimpfungen*					
Gelbfieber	Spätestens 4 Wo vor SOT	1 Dosis	KI, nicht empfohlen	KI	Nicht empfohlen
Dengue	Spätestens 4 Wo vor SOT 2. Dosis	2 Dosen im Abstand von 3 Monaten	KI, nicht empfohlen	KI	Nicht empfohlen
Mpox	Bis zu 2 Wo vor SOT	Wenn indiziert: 2 Dosen im Abstand von 4 Wo	Wenn indiziert	–	Kann unter Immunsuppression gegeben werden; Auffrischung nach 2–5 Jahren bei dauerhaftem Risiko
Chikungunya (lebend)	Bis spätestens 4 Wo vor SOT	1 Dosis	KI, nicht empfohlen	KI	–

*COVID-19* „Coronavirus disease 2019“, *DTPP* Diphtherie/Tetanus/Pertussis/Polio, *GI* Grundimmunisierung, *GvHD* Graft-versus-Host-Erkrankung, *HiB* *Haemophilus influenzae* Typ B, *HPV* humanes Papillomavirus, *IG* Immunglobulin, *inakt* inaktiviert, *KI* Kontraindikation, *M.E.* Meningokokkenenzephalitis, *MMR* Masern-Mumps-Röteln, *Mo* Monat(e), *PCV* Pneumokokken-Konjugat-Impfstoff, *PEP* Postexpositionsprophylaxe, *RSV* humanes respiratorisches Synzytialvirus, *SOT* solide Organtransplantation, *TX* Transplantation, *VE* Vakzineffizienz, *VZV* Varicella-Zoster-Virus, *Wo* Woche(n)

#### 6.3.14 Impfungen bei HIV

Bei HIV-Infizierten ist das Impfansprechen nach Erreichen der virologischen Suppression und nach Immunrekonstitution besser als bei virämischen Personen. Daher sollte, wenn möglich, darauf gewartet werden, dass die Viruslast unter der Nachweisgrenze (oder zumindest niedrig) ist und die CD4-Zellzahl ≥ 200 Zellen/μl oder ≥ 15 % beträgt, bevor mit einer Impfserie begonnen wird [83, 86]. Mit den aktuell verwendeten HIV-Medikamenten ist eine virologische Suppression oder zumindest ein biologisch vergleichbarer Zustand gewöhnlich innerhalb von Wochen bis max. 3 Monaten erreicht. Sollten Impfungen bereits vor virologischer Suppression bzw. bei einer CD4-Zellzahl < 200 Zellen/μL (oder < 15 %) erfolgt sein, dann sollten Auffrischungsimpfungen nach virologischer Suppression/Immunrekonstitution bzw. Antikörperbestimmungen (z. B. Tollwut, FSME, Hepatitis A/B) erwogen werden. Nachdem Impfantworten bei HIV-Infizierten geringer ausfallen können (geringere Serokonversionsraten und schnellerer Titerabfall) sollten keine Schnellimmunisierungsschemata (wie z. B. Tollwut, Hepatitis A/B) angewandt werden. Sollten postexpositionelle Impfungen nach einem tollwutsuspekten Kontakt nötig werden – sollten alle empfohlenen Impfdosen/Immunglobulin und Booster sorgfältig verabreicht werden.

Empfohlen sind alle altersentsprechenden Impfungen laut österreichischem Impfplan – sowie COVID-19 und Influenza jährlich, Hepatitis A (v. a. MSM) + B, HPV (bis 45 Jahre – Nutzen bei älteren Erwachsenen individuell besprechen), Meningokokken ACWY + B (unabhängig von HIV-Stadium/CD4-Zellzahl), Mpox bei Risikoexposition, Pneumokokken, RSV, Herpes Zoster bei fortgeschrittener HIV-Infektion oder nicht therapierten Personen mit HIV.

Lebendimpfungen wie z. B. MMR, Varizellen, Gelbfieber sind bei < 200 CD4-Zellen/μL (oder < 15 %) oder AIDS kontraindiziert. Immunglobuline sollen nach Exposition verabreicht werden, wenn der Patient ungeimpft oder seronegativ ist. Eine Ausnahme bildet die Mpox-Impfung – eine lebend-attenuierte, aber nicht replizierende, modifizierte Vaccinia-Ankara-Virus-Vakzine (Jynneos, Imvamune® oder Imvanex®), die auch bei < 200 CD4-Zellen/μl (oder < 15 %) sicher angewandt werden kann, jedoch kann die Immunogenität vermindert sein ([86]; Tab. 24).

**Tab. 24:** Impfen bei HIV. (Adaptiert nach [86])

**Infektion**	**Impfschema**	**Kommentar**
Influenza	Jährlich	–
HPV	3 Dosen (9–45 Jahre), danach individuell	Bei gut kontrollierter HIV-Infektion (CD4 > 500 Zellen/μl), Serokonversion nach 2 Dosen
Hepatitis B	Ggf. Grundimmunisierung mit adjuviertem oder Hochdosisimpfstoff (0, 1, 6 Monate). Bei Seronegativität Boosterdosis bis HBs-AK ≥ 100 IU/l. Bei Nonrespondern: 3 Dosen mit adjuviertem oder alternativ Hochdosis-Hepatitis-B-Impfstoff (0, 1, 6 Monate)	AK-Kontrolle zur Bestätigung des Impfansprechens. Bei niedrigem CD4-Count (< 200 Zellen/μl), vor HBV-Impfung antiretrovirale Therapie. CpG-adjuvierten Impfstoff für Grundimmunisierung. Bei HBc positiv/HBs-Antigen negativ/HBs-Antikörper negativ: HBV-Impfung empfohlen
Hepatitis A	Bei Seronegativität Grundimmunisierung	Impfung empfohlen, besonders bei MSM, Drogenabusus, aktiver Hepatitis B oder C, chron. Lebererkrankung, SexarbeiterInnen und anderen Expositionsrisiken
Meningokokken	1 Dosis Meningokokken ACWY Impfstoff* 2 Dosen Meningokokken B Impfstoff (0, 1–2 Monate)	*Bei gut kontrollierter HIV-Infektion. Auffrischungen alle 5 Jahre bei weiterer Indikation. Besonders bei Reise, enger Kontakt zu Kindern, MSM, Einnahme von PrEP, Behandlung in Kliniken für sexuelle Gesundheit
Pneumokokken	1 × PCV20 nach 8 Wochen 1 × PCV21 Bei gut kontrollierter HIV-Infektion, 1 Dosis PCV21 ausreichend	Bei zuvor gegen Pneumokokken geimpften (PCV20, PCV15, PCV13, oder PPV23) 1 × PCV21 mit Mindestabstand 8 Wochen zur Vorimpfung. (Für weitere Auffrischungsimpfungen siehe auch aktueller Ö Impfplan). PCV20 wegen Epidemiologie von Serotyp 4
VZV	Bei Seronegativität 2 Dosen lebend-attenuierter VZV-Impfstoff (≥ 4 Wochen Mindestabstand)	Kontraindikation: Immunsuppression Grad III; CD4 ≤ 200 Zellen/µL Antikörperüberprüfung des Impferfolges
Herpes Zoster	2 Dosen mit rekombinantem Impfstoff (0, 2–6 Mo)	Impfstoff ab 18 Jahren für Risikopatienten zugelassen
COVID-19	Jährlich	COVID-19-Impfung für alle Personen mit HIV, ungeachtet der CD4-Zellzahl und Viruslast empfohlen. Geringere Antikörperantwort in Personen mit fortgeschrittener HIV-Infektion (CD4-Zellen < 200/μL) bzw. Virämie
RSV	1 Dosis	Empfohlen für alle Personen mit HIV ≥ 18 und besonders ≥ 60 Jahren
Mpox	*Präexpositionell*: 2 Dosen (0,5 µL) s.c. mit Abstand von 4 Wochen. *Postexpositionell*: innerhalb von 4 Tagen nach Exposition, 2 Dosen (0,5 µL) s.c. mit Abstand von 4 Wochen	Präexpositionelle Impfung soll allen mit PrEP, SexarbeiterInnen, Transgenderpersonen und Personen mit Hochrisikoexposition angeboten werden. Keine Indikation zur Impfung von Personen mit durchgemachter Mpox-Infektion. Eine Auffrischungsimpfung ist bei andauerndem Risiko/Indikation nach 2–5 Jahren empfohlen (österr. Impfplan). Bei Impfstoffknappheit, Priorisierung von Personen mit HIV mit CD4-Zellen < 350/µL oder nachweisbarer HIV-Virämie (WHO). Die 1. Dosis der postexpositionellen Impfung sollte innerhalb von 4 Tagen (bis maximal 14 Tagen) nach Exposition verabreicht werden
*Haemophilus influenzae* Typ B	1 Dosis	Bis 18 Jahre, wenn zuvor keine HiB-Impfung erfolgte

*AK* Antikörper, COVID-19 Coronavirus disease 2019, *CpG* Cytosin-Phosphat-Guanin, *HBc* Hepatitis B-Core-Antigen, *HBs* s-Oberflächenantigen bzw. s‑Oberflächenantikörper des Hepatitis-B-Virus, *HiB Haemophilus influenzae* Typ B, *HIV* humanes Immundefizienzvirus, *HPV* humanes Papillomavirus, *MSM* Männer, die Sex mit Männern haben, *PCV* Pneumokokken-Konjugat-Impfstoff, *PPV* Pneumokokken-Polysaccharid-Impfstoff, *PrEP* Präexpositionsprophylaxe, *RSV* respiratorisches Synzytialvirus, *s.c.* subkutan, *VZV* Varicella-Zoster-Virus, *WHO* Weltgesundheitsorganisation

#### 6.3.15 Impfen bei Asplenie/Hyposplenie

Bei Asplenie/Hyposplenie wird insbesondere das Impfschema D (Abb. 12) sowie die jährliche Influenza- und COVID-19-Impfung empfohlen.

## 7 Wenn nicht geimpft werden kann

Wenn unter immunsuppressiver Therapie ein fehlender Schutz gegen Masern, Mumps, Röteln oder Varizellen vorliegt und ein Kontakt mit einem Infizierten (Masern, Varizellen) stattgefunden hat, gibt es die Möglichkeit einer postexpositionellen Prophylaxe mit Immunglobulinen für Masern oder Varizellen-PEP (siehe österr. Impfplan; [6]).

Bei hochgradig immunsuppressiven Therapien (Anti-CD20-Antikörper oder zellgerichtete Therapien) kann die Gabe von Immunglobulinen sinnvoll sein, solange das Ansprechen auf Impfungen (aufgrund fehlender B‑ und/oder T‑Zellen) stark eingeschränkt ist (siehe Abschnitt 3.3) und/oder eine Infektionsanfälligkeit vorliegt.

### 7.1 Immunglobuline

Nach Immunglobulingabe ist kein Abstand zu inaktivierten Impfstoffen nötig.

Bei Lebendimpfstoffen muss gemäß österreichischem Impfplan [6] ein Abstand eingehalten werden, da die Verabreichung von Immunglobulin die Wirkung von Viruslebendimpfstoffen beeinflussen/reduzieren können. Bezüglich des Abstands einer Lebendimpfung zur Immunglobulinapplikation ist die Fachinformation der jeweiligen Produkte heranzuziehen. Bei Mumps, Röteln und Varizellen beispielsweise kann die Immunantwort über einen Zeitraum von mindestens 6 Wochen bis zu 3 Monaten negativ beeinflusst werden. Es soll daher ein Zeitraum von **3 Monaten** verstreichen, bevor eine **Mumps‑, Röteln-, und Varizellenimpfung** gegeben werden kann. Bei **Masern** kann dieser Zeitraum auch **8 bis 12 Monate** andauern! Deshalb empfiehlt sich bei Personen, die eine Masernimpfung nach Immunglobulingabe erhalten, den Antikörperspiegel zu überprüft.

Bei Gelbfieberimpfung muss in der Regel kein Abstand (bis maximal 3 Monate) nach Immunglobulingabe eingehalten werden, da unwahrscheinlich ist, dass in europäischen humanen Immunglobulinpräparaten große Mengen an gelbfieberspezifischen Antikörpern enthalten sind, die das Impfvirus inaktivieren könnten.

### 7.2 Antivirale Prophylaxe und Therapie

Eine antivirale Abschirmung (Aciclovir gegen Herpes simplex Virus [HSV] und VZV) wird routinemäßig nach Stammzelltransplantation oder T‑Zell-gerichteten Therapien für mindestens 12 Monate gegeben. Für die CMV-Prophylaxe wird Letermovir bis zum Tag 200 eingesetzt.

Im Falle eines Kontakts einer VZV-negativen immunsupprimierten Person mit einer varizelleninfizierten Person muss (besonders wenn kein Immunglobulin verfügbar ist) eine Prophylaxe mit Aciclovir für 1 Woche durchgeführt werden.

### 7.3 Umgebungsprophylaxe

Ein wichtiger Teil in der Betreuung von immunsupprimierten Personen ist, dass alle Kontaktpersonen (An- und Zugehörige, Betreuungspersonen etc.) in die Impfversorgung eingeschlossen werden sollen. Alle Impfungen des österreichischen Impfplans sollen gemäß den altersentsprechenden Empfehlungen vorliegen bzw. aufgefrischt werden (siehe Tab. 3).

## 8 Kurzer Überblick zu Reiseimpfungen bei Immunsuppression/Immundefekten

Alle Reiseimpfungen sind Indikationsimpfungen. Der Begriff Indikationsimpfung bezeichnet alle Impfungen, welche im Gegensatz zu Standardimpfungen nur unter bestimmten Voraussetzungen bzw. für bestimmte Risikogruppen mit einem erhöhten Expositions‑, Erkrankungs- oder Komplikationsrisiko sowie zum Schutz Dritter empfohlen werden. Bei nicht vermeidbaren Reisen in Endemiegebiete muss eine Einzelfallentscheidung zur Durchführbarkeit notwendiger bzw. verpflichtender Impfungen erfolgen.

Bei Reiseimpfungen wird der Schweregrad der Immunsuppression berücksichtigt (Tab. 1 und 2). Die Tab. 25 zeigt einen Überblick über wichtige Aspekte, die bei Reiseimpfungen im Setting der Immunsuppression berücksichtigt werden müssen.

**Tab. 25:** Reiseimpfungen bei Personen mit Immunsuppression

**Impfung**	**Impfempfehlung**			**Anmerkungen und besondere Indikationen**	**Serologische Prüfung**			**Literatur**
Grad der ISP	I	II	III		I	II	III	
*Lebendimpfstoffe*^*a*^								
Zu beachten: Impfungen mit Lebendimpfstoffen sollten mindestens 4 Wochen vor Beginn einer immunmodulatorischen Therapie bzw. Organtransplantation abgeschlossen sein [182]								
Cholera Lebendimpfstoff	(J)	(J)	N	Kontraindiziert (Grad III), alternativen inaktivierten Impfstoff anwenden	N	N	N	Keine Daten zu ISP
Dengue	(J)	N	N	Kontraindiziert (Grad I–III)	N	N	N	Keine Daten zu ISP
Chikungunya Lebendimpfstoff	(J)	(J)	N	Kontraindiziert (Grad I–III), alternativen VLP-Impfstoff anwenden	N	N	N	Keine Daten zu ISP
Gelbfieber	J	(J)	N	Bei immunsupprimierten Personen kontraindiziert	N	N	N	[183, 184]
				Lebendimpfstoff mit besonders hoher Replikationskapazität				
Typhus (Vivotif)	J	N	N	Bei Grad-II/III-ISP sollte ein inaktivierter Typhusimpfstoff verwendet werden	N	N	N	Kein Daten zu ISP
				Kontraindikation: generell bei Grad-III-ISP				
*Inaktivierte Impfstoffe*^*a*^								
Zu beachten: Impfungen mit inaktivierten Impfstoffen sollten mindestens 2 Wochen vor Beginn einer immunmodulatorischen Therapie bzw. Organtransplantation abgeschlossen sein [182]								
Cholera	J	J	(J)	Serologische Überprüfung nicht routinemäßig möglich	N	N	N	Nur bei Reisen in Gebiete mit Ausbruchssituation
Hepatitis A	J	J	J	Bei ISP ist ein komplettes 2‑Dosen-Schema (0–6 Monate) wichtig	N	b. B.	J	[185–187]
				Titerkontrolle empfohlen				
				Bei inkomplettem Impfschema IG zusätzlich empfohlen				
Chikungunya inaktiviert	J	J	(J)	Grad-III-ISP fragliches Ansprechen, serologische Kontrolle derzeit nicht möglich	N	N	N	Keine Daten zu ISP
Japanische Enzephalitis	J	J	(J)	Keine Daten bei Grad-III-ISP	N	N	N	Nur bei hohem Expositionsrisiko empfohlen
Tollwut	J	J	J	Details siehe 8.2	N	(J)	J	Keine Daten zu ISP
Typhus	J	J	J	Impferfolgskontrolle derzeit nicht möglich	N	N	N	Keine Daten zu ISP

Quellen: laut Angaben in der Tabelle

J = empfohlen, N = nicht empfohlen, (J) = nur nach entsprechender Nutzen-Risiko-Abwägung empfohlen, b. B. = nur bei Bedarf

*IG* Immunglobulin, *ISP* Immunsuppression, *VLP* virusartige Partikel

^a^ Bei Reiseimpfungen häufig angewendete Schnellimpfschemata sollten bei immunsupprimierten Personen nicht zum Einsatz kommen [188]

### 8.1 Gelbfieber

Gelbfieber wird durch das Gelbfiebervirus aus der Familie der Flaviviridae hervorgerufen und durch Stechmücken der Gattung Aedes übertragen.

Die Hauptendemiegebiete liegen in Afrika südlich der Sahara und Südamerika. Das Virus zirkuliert zwischen Stechmücken und infizierten Affen, Nagern und Beuteltieren, beim urbanen Gelbfieber auch zwischen Menschen. Nach einer Inkubationszeit von 3 bis 10 Tagen manifestiert sich die Erkrankung meist als fieberhafter und milder Infekt. Die schwere Form von Gelbfieber geht mit hohem Fieber, Hepatitis und Blutungen (Petechien, Hämatemesis, Meläna) einher und zeigt eine hohe Letalität (10–20 %). Es gibt keine spezifische Therapie.

Als Prävention wird einmalig ein lebend-attenuierter Impfstoff verabreicht, der laut WHO nach 10 Tagen einen lebenslangen Schutz bietet. Der österreichische Impfplan empfiehlt jedoch jedenfalls eine zweite Impfung nach frühestens 10 Jahren (vor Einreise in ein aktives Endemiegebiet), aufgrund der geringen Datenlage für einen lebenslangen Schutz einer einmaligen Impfung bei Reisenden aus Nichtendemiegebieten.

ACHTUNG: Der Impfstoff wird auf Hühnerembryonen gezüchtet und ist bei Personen mit Hühnereiweißallergie kontraindiziert.

Weiters ist die Impfung kontraindiziert bei Säuglingen < 9 Monaten, bei immunsupprimierten Personen mit primären oder sekundären Immundefekten und bei Schwangeren/Stillenden – wobei bei Schwangeren/Stillenden nach strenger Nutzen-Risiko-Analyse eine Impfung erwogen werden kann.

Schwere Nebenwirkungen („yellow fever vaccine associated neurological disease“, YEL-AND; „yellow fever vaccine associated viscerotropic disease“, YEL-AVD) treten selten nach der ersten Gelbfieberimpfung auf. Das Risiko für diese schweren Nebenwirkungen besteht hauptsächlich bei Kindern < 1 Jahr und bei Personen ≥ 60, besonders ≥ 70 Jahren sowie bei Personen mit Erkrankungen der Thymusdrüse. Wegen des potenziellen Nebenwirkungsrisikos ist eine strenge Indikationsstellung der Impfung sowie eine Nutzen-Risiko-Abwägung, Aufklärung über die Nebenwirkungen und Dokumentation der Aufklärung unumgänglich.

Nur eine von WHO-zertifizierten Impfstellen im internationalen Impfpass dokumentierte Gelbfieberimpfung wird im internationalen Reiseverkehr als Nachweis anerkannt. Die Liste der Risikoländer und die geltenden Impf- und Einreisevorschriften können auf der Website der WHO eingesehen werden [189]. Bei immunsupprimierten Personen, bei denen eine Gelbfieberimpfung kontraindiziert ist, kann bei Reisen in Länder mit verpflichtender Gelbfieberimpfung ein Ausschluss für ein Impfzertifikat ausgestellt werden. Dies ist aber nur anzuraten, wenn die Gelbfieberimpfung nur ein Formalerfordernis für die Einreise darstellt, aber nicht, wenn ein tatsächliches Expositionsrisiko für die betroffene Person vorliegt. Reisende müssen darüber aufgeklärt werden, dass ein Ausschlusszertifikat von den Behörden nicht anerkannt werden muss.

#### 8.1.1 Anwendung der Gelbfieberimpfung in besonderen Fällen

*Multiple Sklerose:* In den letzten Jahren sind Studien publiziert worden, die ein individuelles Vorgehen bei Personen mit Immunsuppression möglich machen. Da die Erkrankung Multiple Sklerose per se keine Immunsuppression auslöst, können Betroffene ohne Therapie entgegen dem früheren Dogma gegen Gelbfieber geimpft werden. Unter bestehender ISP-Grad-II-Therapie, z. B. Glatirameracetat, Interferon‑β, ist laut neueren Publikationen [190] die Gelbfieberimpfung möglich, ohne dass sich das Risiko eines Erkrankungsschubes erhöht. Bei Personen, die mit Grad-III-Immunsuppressiva behandelt werden, ist während der Therapie keine Gelbfieberimpfung möglich; hier muss, wie bei allen anderen Lebendimpfungen, ein entsprechendes Intervall bis zur Immunrekonstitution (Immunoprofiling!) eingehalten werden. Die Therapie kann ein Monat danach wieder fortgesetzt werden. Bei fulminanter MS-Reaktivierung ist eine Gelbfieberimpfung jedenfalls kontraindiziert [191].

*Rheumatoide Arthritis:* Bei Immunsupprimierten unter niedrig dosierter MTX-Therapie (< 25 mg/Woche) gibt es Daten zur Gelbfieberimpfung unter der Therapie, wobei die erstmalige Gelbfieberimpfung sicher und immunogen war [192]. Bei laufender Behandlung mit Grad-III-Immunsuppressiva ist die Gelbfieberimpfung bei einem klinisch stabilen Krankheitsverlauf während einer Therapiepause möglich (entsprechende Pausen – siehe Kapitel 6).

*HIV:* Die Gelbfiebererstimpfung ist bei Personen mit HIV mit CD4-T-Zellen > 200 Zellen/µL sicher und immunogen, allerdings wurde bei den Betroffenen eine postvakzinale, impfstoffinduzierte (17DD) Virämie zweimal häufiger beobachtet als bei Gesunden. Es kam auch zu kurzfristiger Reduktion von Lymphozyten, Neutrophilen und Thrombozyten, die sich aber innerhalb kurzer Zeit wieder regenerierten. Aufgrund einer möglicherweise reduzierten Langzeitwirkung wird eine routinemäßige Auffrischungsimpfung nach 10 Jahren vor Einreise in ein Endemiegebiet empfohlen [193, 194].

*Asplenie:* Bei Asplenie stellt das Fehlen einer Milz prinzipiell keine Kontraindikation gegenüber einer Gelbfieberimpfung dar, allerdings kann die zugrunde liegende Erkrankung eine Kontraindikation darstellen, z. B. hämatoonkologische Erkrankungen oder immunvermittelte Erkrankungen mit immunsuppressiver Therapie. Daher ist eine sorgfältige Anamnese zu erstellen [134].

*Thymuserkrankungen: *Bei Thymuserkrankungen, die mit einer abnormalen Immunzellfunktion assoziiert sind, z. B. Myasthenia gravis oder Thymome, ist die Gelbfieberimpfung kontraindiziert. Es gibt keine Evidenz für Immundysfunktion oder ein erhöhtes Risiko für schwere Nebenwirkungen nach Gelbfieberimpfung bei Personen, die eine Thymektomie im Zuge einer Herzoperation durchlaufen haben [194].

### 8.2 Tollwut

Tollwut, ausgelöst durch neurotrope Rhabdoviren aus der Familie der Lyssaviren, ist eine Zoonose, die häufig durch Biss- oder Kratzverletzungen durch infizierte Tiere übertragen wird. Man unterscheidet die terrestrische Tollwut, unterteilt in sylvatische (Wildtiere) und urbane Form (Haustiere wie Hund, Katze), von der Fledermaus-Tollwut. Weltweit vorkommend (besonders Afrika und Asien) konnte die terrestrische Tollwut in Europa eingedämmt werden. Mittel- und Westeuropa gelten als frei von terrestrischer Tollwut. Allerdings kommt die durch die Fledermaus übertragene Tollwut auch in Europa ganz vereinzelt vor [195]. Für einen ungeschützten Menschen kommt es nach Infektion in jedem Fall zu einer letalen Erkrankung.

Derzeit stehen zwei inaktivierte Impfstoffe zur Verfügung, die zur prä(PrEP)- und postexpositionellen Prophylaxe (PEP) eingesetzt werden können: Rabipur, eine Ganzvirusvakzine, Herstellung auf Hühnerembryonalzellen, sowie Verorab, ein auf Vero-Zellen hergestellter Impfstoff. Es sind verschiedene Impfschemata zur präexpositionellen Prophylaxe zugelassen.

Alle Personen unter Immunsuppression sollen ausschließlich das konventionelle Grundimmunisierungsschema bestehend aus 3 Teilimpfungen erhalten: Tag 0, 7, 21 bis 28. Bei Immundefizienz, ab ISP-Grad II, soll nach 4 bis 8 Wochen das Impfansprechen bevorzugt mit dem Nachweis von neutralisierenden Antikörpern überprüft und ggf. eine weitere Dosis verabreicht werden.

Zur PEP werden bei einem Tierkontakt ab Kategorie II – das entspricht nicht blutenden Verletzungen oder Belecken von verletzter Haut – **ab ISP-Grad II, auch wenn eine Grundimmunisierung durchgeführt wurde, Impfungen (Tag 0–3–7–14–28) und spezifisches Immunglobulin verabreicht**. Bei ISP-Grad 0–I sind bei vollständiger Grundimmunisierung zwei Impfungen im Abstand von 3 Tagen ausreichend [6, 196].

ACHTUNG: In vielen Ländern ist der Zugang zu Immunglobulinen für eine PEP eingeschränkt.

Eine kleine, retrospektive Studie aus Frankreich konnte zeigen, dass eine PEP ohne vorherige Grundimmunisierung bei Personen unter TNF-α-Inhibitor-Therapie oder SOT ein adäquates serologisches Ansprechen induzierte. Bei 5 von 6 Nonrespondern konnten durch eine zusätzliche Impfdosis adäquate Antikörpertiter erzielt werden. Immunoseneszenz schien hier ein Grund für ein inadäquates Impfansprechen zu sein [197].

Eine prospektive Studie aus den Niederlanden mit 52 Personen zeigte eine gute Immunogenität der Tollwutimpfung unter csDMARD oder TNF-α-Inhibitor-Therapie. Darüber hinaus war auch die Boosterfähigkeit nach einem Jahr (gemessen 7 Tagen nach dem ersten Booster) in 90 % gegeben [198].

### 8.3 Dengue

Die durch Gelbfiebermücken (Aedes spp.), aber auch asiatische Tigermücken übertragene Virusinfektion führte 2024 zu weltweit 13 Mio. gemeldeten Infektionen, davon 8500 Todesfälle. Die vier zirkulierenden Serotypen (DENV 1–4) hinterlassen für kurze Zeit eine Kreuzimmunität. Eine erneute Infektion, aber auch die Erstinfektion, birgt aber ein 2‑ bis 4 %iges Risiko eines Dengue-Schock-Syndroms („antibody dependent enhancement“, ADE).

Als Reiseimpfung wird der tetravalente, lebend-attenuierte Qdenga-Impfstoff angeboten. Als Basis dient hier das DENV2; die anderen Serotypen wurden verändert und als Hüllprotein hinzugefügt. Nach der Impfung kann es vorübergehend zu einer Virämie mit entsprechender Symptomatik einer leichten Dengue-Infektion (Impf-Dengue) kommen. Eine Übertragung des Impfvirus auf andere Menschen wurde bisher nicht dokumentiert.

Zugelassen ab 4 Jahren, werden die beiden Teilimpfungen im Abstand von 3 Monaten subkutan verabreicht. Das Risiko einer Anaphylaxie ist mit 147/Million Impfungen hoch und ist damit in der Aufklärung zu kommunizieren [199].

Die Impfempfehlung bezieht sich derzeit vorwiegend auf Personen, die bereits einmal eine Dengue-Infektion durchgemacht haben. Der Einsatz des Dengue-Impfstoffs bei bislang Dengue-unexponierten Personen wurde bis dato sehr restriktiv gehalten. Die Befürchtung hinsichtlich möglicher Nebenwirkungen in Sinne eines ADE ohne vorangegangene Dengue-Infektion wurde aber bislang nicht bestätigt. Bei Reisen während Ausbrüchen, Langzeitaufenthalten oder wiederholten Reisen in Endemiegebiete kann die zweimalige Impfung unter Nutzen-Risiko-Abwägung jedoch sinnvoll sein. Der Impfschutz gegenüber DENV-Subtypen 3 und 4 ist derzeit noch unklar.

Die Impfindikation für gesunde Personen ist dem österreichischen Impfplan zu entnehmen. Da es sich um eine Lebendimpfung handelt, ist sie bei immunsupprimierten Personen (ISP-Grad III) jedenfalls kontraindiziert. Eine sorgfältige reisemedizinische Aufklärung ist daher besonders wichtig [6, 200].

### 8.4 Chikungunya

Chikungunya, ein Virus aus der Familie der Togaviren, wird ebenfalls durch die Gelbfiebermücke und die asiatische Tigermücke übertragen und tritt immer wieder ausbruchsartig auf. Im Jahr 2024 wurden 620.000 Fälle gemeldet, darunter etwa 200 Todesfälle. Die Hauptlast der Erkrankung lag in Brasilien [201]. Es gibt 4 Genotypen und eine Infektion mit einem der Genotypen hinterlässt eine Immunität gegenüber allen anderen. Das Hauptmerkmal dieser Infektion sind Gelenkschmerzen, die bei ca. einem Drittel der Erkrankten > 12 Monate andauern können. Eine Impfung wird empfohlen für Reisen in Gebiete mit Ausbruchssituation oder bei Reisen in Endemiegebiete mit Langzeitaufenthalt und gesundheitlichen Risikofaktoren. Besonders gefährdet sind Personen ab 60 Jahren und Personen mit Komorbiditäten.

Aktuell stehen zwei Impfstoffe zur Verfügung. Der inaktivierte Impfstoff, Vimkunya, auf Basis von nichtreplizierenden Virus-like Particles, ist ab 12 Jahren zugelassen und wird einmalig intramuskulär appliziert. Über die klinische Wirksamkeit und Wirkdauer bei Immunsupprimierten liegen derzeit keine Daten vor. Ein Lebendimpfstoff, Ixchiq, wurde bereits 2024 in der EU zugelassen. Es wird eine einmalige intramuskuläre Impfung verabreicht, welche eine langanhaltende Immunantwort erzeugt. Eine gentechnisch veränderte Virusinformation und die dadurch bedingte Verlangsamung der Replikationsfähigkeit führen zu einer starken Immunantwort. Die Anwendung des Lebendimpfstoffs ist für Personen mit jeglicher Immunsuppression nicht empfohlen, da es mit dem inaktivierten Impfstoff, Vimkunya, eine sichere Alternative gibt.

### 8.5 Mpox

Mpox ist eine ursprünglich zoonotische Virusinfektion, verursacht durch das Mpox-Virus, welches zu den Orthopockenviren zählt. Es ist verwandt mit den humanen Pockenviren. Ursprünglich endemisch in Zentralafrika mit dem Tierreservoir in Nagern kam es wohl durch Konsum infizierter Tiere zu humanen Fällen. Die Übertragung kann im familiären Kontext über Oberflächen und Textilien, besonders aber über erregerhaltige Effloreszenzen, Körperflüssigkeiten, engen Hautkontakt und auch fetomaternal erfolgen.

In den letzten Jahren kam es zu weltweiten Ausbrüchen mit Meldung aus 110 Ländern. 2022 (Clade II) und 2024 (Clade Ib) wurde durch die WHO eine gesundheitliche Notlage ausgerufen. Hauptsächlich kam es in MSM-Communities zu Mensch-zu-Mensch-Übertragung. Zusätzlich dürfte auch die schwindende Immunität gegen Pocken (Pockenimpfung bis 1981) eine zusätzliche Rolle für die pandemische Verbreitung spielen.

Die aktuell zugelassene Impfung beruht auf dem modifizierten Vakziniavirus Ankara (MVA) und ist ein Drittgenerations-, nichtreplizierender Impfstoff. In Europa ist er unter dem Namen Imvanex, in den USA als Jynneos und in Kanada als Imvamune zur prä- und postexpositionellen Prophylaxe ab 12 Jahren zugelassen (EMA-CHMP mit 29.6.2026: ab 2 Jahren [202]). Es werden 2 Dosen subkutan im Abstand von 28 Tagen verabreicht. Falls in der Vergangenheit eine Pockenimpfung verabreicht wurde, ist bei Immunkompetenten eine einmalige subkutane Impfung ausreichend. Bei Immunsuppression sollen immer 2 Impfungen erfolgen. Dieser Impfstoff kann auch bei höhergradiger Immunsuppression und HIV angewendet werden, wenn auch mit einem verringerten Impfansprechen zu rechnen ist. Die Impfwirksamkeit (VE) liegt bei 85 % bei Immunkompetenten und ist reduziert bei Personen mit HIV-Infektion [203].

Als PEP soll die Impfung frühestmöglich verabreicht werden, längstens nach 14 Tagen, im selben Schema wie die PrEP. Als PEP wird die Impfung off-label altersunabhängig (< 12 Jahren) empfohlen.

Die Mpox-Impfung ist keine generelle Reiseimpfung. Sie ist empfohlen für Reisen in Endemiegebiete mit engem Kontakt zur Bevölkerung (Gesundheitswesen, Entwicklungshilfe) oder für Personen mit individuellem Risikoverhalten [6, 204].

### 8.6 Ebola

Das Ebolavirus gehört zur Familie der Filoviren und wird in 5 Spezies unterteilt (Zaire [EBOV], Sudan [SUDV], Tai Forest, Bundibugyo und Reston [letztes gilt als nicht humanpathogen]), die vor allem in Subsahara-Afrika verbreitet sind und immer wieder zu lokalen Ausbrüchen führen. Es wird angenommen, dass Flughunde und Fledermäuse das Virusreservoir bilden und deren roher bzw. ungenügend erhitzter Verzehr zur Ansteckung von Menschen führt. In weiterer Folge kann das Virus von Mensch zu Mensch durch direkten Kontakt mit Körperflüssigkeiten eines Erkrankten (Blut, Stuhl, Speichel, Samenflüssigkeit, Urin, Erbrochenes) übertragen werden. Vor allem medizinisches Personal, An- und Zugehörige sowie Bestattungspersonal haben das höchste Ansteckungsrisiko, wohingegen das Risiko für Reisende als sehr gering einzustufen ist. Die Infektion löst nach einer durchschnittlichen Inkubationszeit von 6 bis 10 Tagen eine schwerwiegende Erkrankung aus. Nach initialen Symptomen wie Fieber, Kopfschmerz, Muskel- und Gliederschmerzen sowie Halsschmerzen und Müdigkeit kommt es in weiterer Folge zu Erbrechen, Diarrhö und Hämorrhagien (blutige Stühle, Blutungen an mukosalen Oberflächen, Hautblutungen) sowie Leber- und Nierenversagen.

Die Therapie ist nach wie vor supportiv, wobei es mittlerweile erste Zulassungen spezifischer monoklonaler Antikörper gegen EBOV (Zaire) gibt: Ebanga (Ansuvimab-zykl) und Inmazeb (Atoltivimab, Maftivimab und Odesivimab-ebgn). Zu den Präventionsmaßnahmen zählen Vorsicht bei der Nahrungszubereitung und Kontaktvermeidung bzw. Tragen von Schutzausrüstung bei Kontakt zu Erkrankten und Verstorbenen und deren Ausscheidungen.

Ein Ebola-Impfstoff auf der Basis von Vektoren, Ervebo, ist aktuell von der EMA zugelassen, jedoch derzeit nicht in der EU erhältlich [205]. Die Zulassung von Mvabea und Zabdeno wurde am 1. Mai 2026 von der EMA auf Antrag der Zulassungsinhaber aus kommerziellen Gründen zurückgezogen [206, 207]. Hinsichtlich der Anwendung bei immunsupprimierten Personen wurde bei HIV sowohl Wirksamkeit als auch Sicherheit gezeigt [208, 209]. Die Anwendung der Impfstoffe ist für Reisende derzeit nicht vorgesehen.

## 9 Wichtige Quellen zu jeweils aktuellen Informationen

- *Aktueller österreichischer Impfplan:*
https://www.sozialministerium.gv.at/Themen/Gesundheit/Impfen/impfplan.html

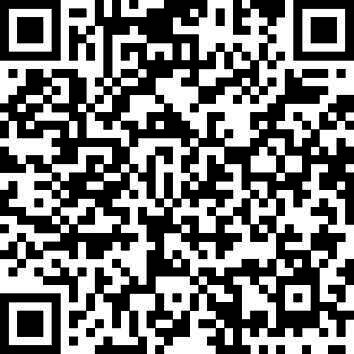

- *Arzneispezialitätenregister des österreichischen Bundesamts für Sicherheit im Gesundheitswesen (BASG):*
https://medikamente.basg.gv.at/de/medicinal-products

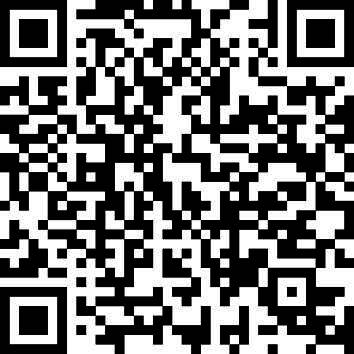

- *Website der europäischen Zulassungsbehörde EMA – Suchmaske für Arzneimittel beim Menschen:*
https://www.ema.europa.eu/en/medicines

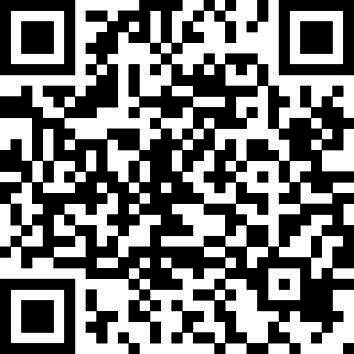

- *Übersicht der EMA über zugelassene COVID-19-Impfstoffe*
https://www.ema.europa.eu/en/human-regulatory-overview/public-health-threats/coronavirus-disease-covid-19/covid-19-medicines

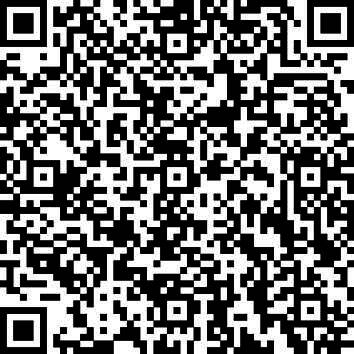

- *Australian Immunization Handbook:*
https://immunisationhandbook.health.gov.au

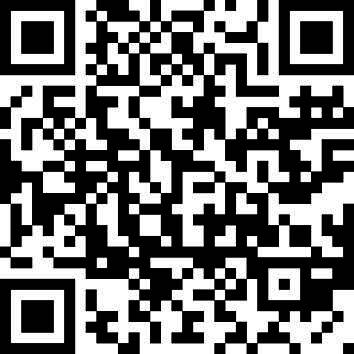

- *Liste der Biologika des Paul-Ehrlich-Instituts (PEI):*
https://www.pei.de/DE/arzneimittel/antikoerper/monoklonale-antikoerper/monoklonale-antikoerper-node.html

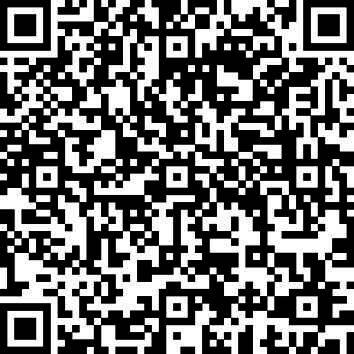

- *Liste der Impfstoffe des Paul-Ehrlich-Instituts (PEI):*
https://www.pei.de/DE/arzneimittel/impfstoffe/impfstoffe-node.html

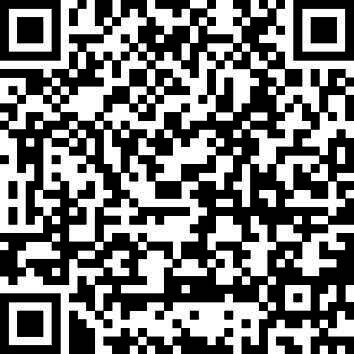

## Data Availability

Alle dieser Arbeit zugrunde liegenden Daten sind in diesem Artikel enthalten.

## Anhang

### 10 Appendix 1 Abkürzungen

- 5‑ASA, 5‑Aminosalicylsäure
- ACE2, Angiotensin-Converting-Enzyme 2
- ADA, Adenosindesaminasemangel
- AHI, Australian Handbook of Immunization
- aHUS, atypisches hämolytisch-urämisches Syndrom
- AIDS, acquired immune deficiency syndrome = erworbenes Immunschwächesyndrom
- ALL, akute lymphatische Leukämie
- ALK, anaplastic lymphoma kinase
- ALPS, autoimmunes lymphoproliferatives Syndrom
- AML, akute myeloische Leukämie
- ANCA, anti-Neutrophile zytoplasmatische Antikörper
- AOIDSM, Adult-onset-Immundefizienzsyndrom mit Anfälligkeit für Mykobakterien durch Anti-IFN-γ-Autoantikörper
- AK, Antikörper
- aP, azellulärer Pertussis-Impfstoff
- APC, antigenpräsentierende Zellen
- APECED, autoimmune polyendocrinopathy with candidiasis and ectodermal dystrophy = autoimmunes polyglanduläres Syndrom Typ 1 (APS Typ 1)
- aRZV, adjuvierter rekombinanter Zoster-Impfstoff
- AS, Adjuvanssystem
- AT, Ataxia teleangiectasia
- ATG, Antithymozytenglobulin
- AxSpA, axiale Spondylarthritis
- AZA, Azathioprin
- BAFF, B-Zell-aktivierender Faktor
- BASG, Bundesamt für Sicherheit im Gesundheitswesen
- BCG, Bacillus Calmette-Guérin
- BCL2, B cell lymphoma 2 = BCL2-Protein
- BCMA, B‑cell maturation antigen = B-Zell-Reifungsantigen
- BCR, B cell receptor = B-Zell-Rezeptor
- BCR-ABL, breakpoint cluster region-Abelson murine leukemia viral oncogene
- bDMARDs, biologische DMARDs
- BiTE, bispecific T cell engager = bispezifischer Antikörper
- BLyS, B‑Lymphozyten-Stimulator
- BRAF, B‑Raf proto-oncogene
- bsDMARDs, Biosimilar biologische DMARDs
- BTKi, Bruton-Tyrosinkinase-Inhibitor
- BTK, Bruton-Tyrosinkinase
- CA, Karzinom
- CAPS, cryopyrinassoziierte periodische Syndrome
- CAR, chimeric antigen receptor = chimärer Antigenrezeptor
- CDC, Centers for Disease Control and Prevention
- CDK4/6, cyclinabhängige Kinasen 4 und 6
- CF, zystische Fibrose
- CED, chronisch-entzündliche Darmerkrankung
- CGD, chronische granulomatöse Erkrankung
- CHMP, Committee for Medicinal Products for Human Use
- CKD, chronic kidney disease = chronische Nierenerkrankung
- CLL, chronisch-lymphatische Leukämie
- CMC, chronische mukokutane Candidiasis durch Anti-IL-17-Autoantikörper
- CML, chronisch myeloische Leukämie
- CMML, chronische myelomonozytäre Leukämie
- CMV, Cytomegalovirus
- COVID-19, Coronavirus disease 2019
- CpG, Cytosin-Phosphat-Guanin
- CRC, colorectal cancer = Kolorektalkarzinom
- csDMARDs, conventional synthetic DMARDs = konventionelle, synthetische DMARDs
- CTLA‑4, cytotoxic T‑lymphocyte-associated protein 4
- CU, Colitis ulcerosa
- CVID, common variable immunodeficiency
- d, Tag
- DC, Dyskeratosis congenita
- Def., Defizienz
- Di/Tet, Diphtherie/Tetanus
- DMARDs, disease modifying anti-rheumatic drugs = krankheitsmodifizierende Antirheumatika
- DMF, Dimethylfumarat
- DNA, desoxyribonucleic acid = Desoxyribonukleinsäure
- DTaP, Diphtherie‑, Tetanus- und azellulärer Pertussis-Impfstoff
- DTPP, Diphtherie, Tetanus, Pertussis, Polio
- dzt., derzeit
- EAA, enthesitisassoziierte Arthritis
- EBV, Epstein-Barr-Virus
- EGFR, epidermal growth factor = epidermaler Wachstumsfaktor
- EGPA, eosinophile Granulomatose mit Polyangiitis
- ELISA, enzyme-linked immuno sorbent assay
- EMA, European Medicines Agency
- EULAR, European Alliance of Associations for Rheumatology
- FA, Fanconi-Anämie
- FcRn, neonataler Fc-Rezeptor
- FI, Fachinformation
- FL, follikuläres Lymphom
- FLT3, Fms-related receptor tyrosine kinase 3
- FMF, familiäres Mittelmeerfieber
- frgl., fraglich
- FSME, Frühsommer-Meningoenzephalitis
- GA, Glatirameracetat
- γc, common-gamma-chain deficiency
- ggf., gegebenenfalls
- GPA, Granulomatose mit Polyangiitis
- GPP, generalisierte pustulöse Psoriasis
- GvHD, graft versus host disease
- HAV, Hepatitis-A-Virus
- HBc, Hepatitis B-Core-Antigen
- HBs, Hepatitis B surface antigen
- HBV, Hepatitis-B-Virus
- HCV, Hepatitis-C-Virus
- HES, Hypereosinophiliesyndrom
- HER2, human epidermal growth factor receptor 2
- HiB, *Haemophilus influenzae B*
- HIGM, Hyper-IgM Syndrome
- HIV, humanes Immundefizienzvirus
- HPV, humanes Papillomavirus
- HS, Hidradenitis suppurativa
- hSBA, human serum bactericidal assay = bakterizider Antikörpertest mit Humanserum
- HSCT, hämatopoetische Stammzelltransplantation
- HSV, Herpes-simplex-Virus
- i. d. R., in der Regel
- ID, Immundefizienz
- IE, internationale Einheiten
- IEI, inborn errors of immunity = angeborene Fehler des Immunsystems
- IFN, Interferon
- IFNAR1 Def, Interferon-α-Rezeptor-1-Defizienz
- IG, Immunglobulin
- IgA, Immunglobulin A
- IgG, Immunglobulin G
- IgM, Immunglobulin M
- IL, Interleukin
- IL7Ra, IL-7α receptor deficiency
- IMiD, immunomodulatory drug = immunmodulatorischer Wirkstoff
- IPV, inaktivierter Polio-Impfstoff
- IRF7 & IRF9, Interferon regulierender Faktor 7 und 9
- ISP, Immunsuppression
- IUIS, International Union of Immunological Societies
- IVDU, intravenous drug use = intravenöser Drogenkonsum
- IVIg, intravenöses Immunglobulin
- IVMP, intravenös verabreichtes Methylprednisolon
- J, Jahr
- JAK, Januskinase
- JC-Virus, John Cunningham Virus
- JCVI, Joint Committee on Vaccination and Immunisation
- JIA, juvenile idiopathische Arthritis
- KG, Körpergewicht
- KHK, koronare Herzkrankheit
- KI, Kontraindikation
- KM, Knochenmark
- KOF, Körperoberfläche
- LAD, Leukozytenadhäsionsdefekt
- LAIV, live attenuated influenza vaccine = lebend-attenuierter Influenzaimpfstoff
- LE, Lupus erythematodes
- Lj, Lebensjahr
- Lm, Lebensmonat
- LNP, lipidbasierte Nanopartikel
- LRTD, lower respiratory tract disease = Erkrankungen der unteren Atemwege
- Lw, Lebenswoche
- MACE, major adverse cardiovascular event = schweres unerwünschtes kardiovaskuläres Ereignis
- MBL, mannosebindendes Lektin
- mcMMAF, Maleimidocaproyl-Monomethylauristatin F
- MDS, Myelodysplastisches Syndrom
- M.E., Meningokokkenenzephalitis
- MEK, mitogen-activated protein kinase
- MET, MET proto-oncogene/hepatocyte growth factor receptor
- MF, Mikrofluidisierung
- MHC, major histocompatibility complex = Haupthistokompatibilitätskomplex
- MKD, Mevalonatkinasedefizienz
- MM, Multiples Myelom
- MMF, Mycophenolat mofetil
- MMR, Masern-Mumps-Röteln-Dreifachimpfung
- MMRV, Masern-Mumps-Röteln-Varizellen-Vierfachimpfung
- MOGAD, Myelin-Oligodendrozyten-Glykoprotein-Antikörper-assoziierte Erkrankung
- mRNA, messenger ribonucleic acid = Messenger-Ribonukleinsäure
- MS, Multiple Sklerose
- MSM, men having sex with men = Männer, die Sex mit Männern haben
- MSMD, Mendelian susceptibility to mycobacterial disease
- mTOR, mechanistic target of rapamycin
- MTX, Methotrexat
- n. d., nicht definiert
- NHL, Non-Hodgkin-Lymphom
- NK-Zellen, natürliche Killerzellen
- NMOSD, neuromyelitis optica spectrum disorder = Neuromyelitis-optica-Spektrum-Erkrankungen
- NSAR, nichtsteroidale Antirheumatika
- NSCLC, non-small cell lung cancer = nichtkleinzelliges Lungenkarzinom
- NT, Neutralisationstest
- NTRK, neurotrophic tyrosine receptor kinase
- NTX, Nierentransplantation/Empfänger einer Nierentransplantation
- OPA, Opsonophagozytose-Antikörper
- OPV, orale Poliovirusvakzine
- PAPA, pyogene Arthritis, Pyoderma gangraenosum und Akne
- PARP, poly(ADP-ribose) polymerase
- PCV oder PNC, pneumococcal conjugate vaccine = Pneumokokken-Konjugat-Impfstoff
- PCP, Pneumocystis jirovecii
- PD‑1, programmed cell death protein 1
- PD-L1, programmed cell death-ligand 1
- PEI, Paul-Ehrlich-Institut
- PEP, Postexpositionsprophylaxe
- PID, primäre Immundefekte
- PLEX, Plasmapherese
- PMR, Polymyalgia rheumatica
- PNH, paroxysmale nächtliche Hämoglobinurie
- PNS, peripheres Nervensystem
- PPSV23, 23-valenter Pneumokokken-Polysaccharid-Impfstoff
- PPV, pneumococcal polysaccharide vaccine = Pneumokokken-Polysaccharid-Impfstoff
- PrEP, Präexpositionsprophylaxe
- PReS, Paediatric Rheumatology European Society
- PRP, Polyribosylribitolphosphat
- PRR, pattern recognition receptors = Mustererkennungsrezeptoren
- PsA, Psoriasis-Arthritis
- PsO, Psoriasis
- q2w, Einmalgabe alle 2 Wochen
- QS, Quillaja saponaria
- RA, rheumatoide Arthritis
- RANKL, receptor activator of nuclear factor κB ligand
- Rel., relativ
- RET, rearranged during transfection
- RFFIT, rapid fluorescent focus inhibition test
- RIC, reduced intensity conditioning
- RKI, Robert-Koch-Institut
- ROS1, ROS proto-oncogene 1
- RSV, humanes respiratorisches Synzytialvirus
- RTX, Rituximab
- rVSV, rekombinantes vesikuläres Stomatitis-Virus
- RZA, Riesenzellarteriitis
- SARS-CoV‑2, severe acute respiratory syndrome Coronavirus 2
- SASP, Sulfasalazin
- sIgA-Mangel, selektive IgA-Defizienz
- S1P, Sphingosin-1-Phosphat
- S1PR, Sphingosin-1-Phosphat-Rezeptor
- s.c., subkutan
- SCID, severe, combined immunodeficiencies = schwere kombinierte Immundefekte
- SIOP, Société Internationale d’Oncologie Pédiatrique
- SLE, systemischer Lupus erythematodes
- SMI, small molecule inhibitor
- SOT, solide Organtransplantation
- SpA, Spondyloarthritis
- SQBA, Squalen-basiertes Adjuvans
- STAT1, signal transducer and activator of transcription 1
- St.p., Status post
- STIKO, Ständige Impfkommission
- SZT, Stammzelltransplantation
- T‑DM1, Trastuzumab-Emtansin
- Tbc, Tuberkulose
- TCR, T‑Zell-Rezeptor
- TIGIT, T cell immunoreceptor with Ig and ITIM domains
- TKI, Tyrosinkinaseinhibitor
- TLR, Toll-like-Rezeptor
- TNF, Tumornekrosefaktor
- TNFR, Tumornekrosefaktorrezeptor
- TRAPS, TNFR-assoziierte periodische Fiebersyndrome
- tsDMARDs, targeted synthetic DMARDs = zielgerichtete, synthetische DMARDs
- TX, Transplantation
- Wo, Woche
- URT, upper respiratory tract = oberer Respirationstrakt
- u. v. a. m., und vieles andere mehr
- V, Varizellen
- v. a., vor allem
- VE, Vakzineffizienz
- VEGF, vascular endothelial growth factor
- VEGFR, vascular endothelial growth factor receptor
- VLP, virus-like particle = virusartiger Partikel
- VPD, vaccine preventable diseases = impfpräventable Krankheiten
- VTE, venöse Thromboembolie
- VZV, Varicella-Zoster-Virus
- WAS, Wiscott-Aldrich Syndrom
- WHO, World Health Organization = Weltgesundheitsorganisation
- XLA, X‑linked agammaglobulinemia
- YEL-AND, yellow fever vaccine associated neurological disease
- YEL-AVD, yellow fever vaccine associated viscerotropic disease
- ZNS, Zentralnervensystem

### 11 Appendix 2

**Tab. 26:** Tabellenverzeichnis

**Tabelle**
Tab. 1 Schweregrade der Immunsuppression mit Beispielen
Tab. 2 Impffähigkeit in Abhängigkeit des Schweregrads der Immunsuppression
Tab. 3 Impfungen vor Therapiebeginn
Tab. 4 Inaktivierte Impfungen während einer immunsuppressiven Therapie und Impferfolgskontrolle
Tab. 5 Antikörperkontrollen zur Überprüfung des Impferfolgs
Tab. 6 Adjuvanzien (weltweit in Verwendung)
Tab. 7 Qualitative Beschreibung der Infektionsrisikokategorien
Tab. 8 Infektionsrisiko durch/mit Therapien in der Rheumatologie
Tab. 9 Infektionsrisiko durch/mit Therapien in der Dermatologie
Tab. 10 HIV – Einteilung nach Grad der Immunsuppression
Tab. 11 Infektionsrisiko durch/mit Therapien in der Gastroenterologie
Tab. 12 Einteilung immunmediierter Erkrankungen und Therapiebeispiele in der Neurologie
Tab. 13 Infektionsrisiko durch/mit Therapien in der Neurologie
Tab. 14 Infektionsrisiko durch/mit (neo)adjuvanten Therapien und palliativen Erstlinientherapien bei soliden Tumoren: beispielgebend bei Lungen‑, Brust- und Kolonkarzinom
Tab. 15 Infektionsrisiko durch/mit Therapien bei hämatologischen Systemerkrankungen
Tab. 16 Beispiele für Hauptursachen für Asplenie und Hyposplenismus
Tab. 17 Typische Beispiele für angeborene Immundefekte, geordnet nach den IUIS-2024-Gruppen
Tab. 18 Immunsuppression im Kindes- und Jugendalter und impfrelevante Konsequenzen
Tab. 19 Verabreichung von inaktivierten und Lebendimpfstoffen an Säuglinge, die biologischen immunsuppressiven Therapien in utero ausgesetzt waren
Tab. 20 Lebend-attenuierte Impfstoffe
Tab. 21 Impfen nach Stammzelltransplantation
Tab. 22 Impfen von Erwachsenen vor und nach Behandlung mit CAR-T- bzw. Zell-basierter Therapie
Tab. 23 Impfen vor und nach SOT
Tab. 24 Impfen bei HIV
Tab. 25 Reiseimpfungen bei Personen mit Immunsuppression

**Tab. 27:** Abbildungsverzeichnis

**Abbildung**
Abb. 1 Allgemeines Vorgehen zu Impfungen bei Personen mit Immunsuppression
Abb. 2 Überblick: zeitliches Impfvorgehen in Abhängigkeit von Impfstoffart und immunsuppressiver Therapie
Abb. 3 Intervalle zwischen Grad-III-immunsuppressiven Therapien und Lebendimpfstoffen
Abb. 4 Fahrplan für Impfprogramme für Risikopatienten
Abb. 5 Ausbildung einer Immunantwort auf einen Impfstoff und deren Hemmung durch immunsuppressive/-modulierende Therapien
Abb. 6 Impfstofftypen und Beispiele
Abb. 7 Wirkweise der mRNA-Impfstoffe
Abb. 8 Einteilung von Autoimmunerkrankungen des rheumatischen Formenkreises
Abb. 9 Impfschema A – Routineimpfungen laut österreichischem Impfplan (Erwachsene ≥ 18 Jahren)
Abb. 10 Impfschema B – saisonale Impfungen
Abb. 11 Impfschema C – indizierte Impfungen ab dem Alter von ≥ 18 Jahren
Abb. 12 Impfschema D – indizierte Impfungen gegen bekapselte Bakterien (alle Altersgruppen)
Abb. 13 Impfen bei angeborenen/primären Immundefekten bei Erwachsenen ≥ 18 Jahren
Abb. 14 Impfen bei Gabe von Glukokortikoiden
Abb. 15 Impfen bei Gabe von Methotrexat und Azathioprin
Abb. 16 Impfen bei Gabe anderer konventioneller Therapeutika bzw. Immunsuppressiva
Abb. 17 Impfen bei Gabe von TNF-α-Inhibitoren
Abb. 18 Impfen bei Gabe von Interleukininhibitoren
Abb. 19 Impfen bei Gabe *derzeit* gängiger JAK-Inhibitoren
Abb. 20 Impfen bei Gabe von Komplementinhibitoren
Abb. 21 Impfen bei Gabe von T‑Zell-Inhibitoren
Abb. 22 Impfen vor/bei Gabe von B‑Zell-Inhibitoren/Depletoren
Abb. 23 Impfen bei Gabe von Checkpoint-Inhibitoren
Abb. 24 Impfen bei Gabe zielgerichteter Therapien in der Onkologie/Hämatoonkologie
Abb. 25 Bispezifische Antikörper und bispezifische T‑Zell-Engager
Abb. 26 Beispielgebende Antikörper-Wirkstoff-Konjugate (ADC)
Abb. 27 Ergänzung: Impfen bei Gabe von Immunsuppressiva mit ISP-Grad III
Abb. 28 Ergänzung: Impfen bei Gabe von Immunmodulatoren mit ISP-Grad II
Abb. 29 Ergänzung: Impfen bei Gabe von Immunmodulatoren mit ISP-Grad I
